# Hydrogel Thermostat Inspired by Photoprotective Foliage Using Latent and Radiative Heat Control

**DOI:** 10.1002/adma.202516537

**Published:** 2025-11-04

**Authors:** Se‐Yeon Heo, Hyung Rae Kim, Yoonsoo Shin, Hyun Su Lee, Hyunkyu Kwak, Do Hyeon Kim, Dong Hyun Seo, Joo Hwan Ko, Hyo Eun Jeong, Sehui Chang, Min Seok Kim, Longnan Li, Jyotirmoy Mandal, Wei Li, Dae‐Hyeong Kim, Young Min Song

**Affiliations:** ^1^ School of Electrical Engineering and Computer Science Gwangju Institute of Science and Technology Gwangju 61005 Republic of Korea; ^2^ School of Electrical Engineering Korea Advanced Institute of Science and Technology Daejeon 34141 Republic of Korea; ^3^ School of Chemical and Biological Engineering and Institute of Chemical Processes Seoul National University Seoul 08826 Republic of Korea; ^4^ Center for Nanoparticle Research Institute for Basic Science (IBS) Seoul 08826 Republic of Korea; ^5^ GIST InnoCORE AI‐Nano Convergence Institute for Early Detection of Neurodegenerative Diseases Gwangju Institute of Science and Technology Gwangju 61005 Republic of Korea; ^6^ GPL Photonics Laboratory State Key Laboratory of Luminescence Science and Technology Changchun Institute of Optics Fine Mechanics and Physics Chinese Academy of Sciences Changchun Jilin 130033 China; ^7^ University of Chinese Academy of Sciences Beijing 100049 China; ^8^ Department of Civil and Environmental Engineering Princeton University Princeton NJ 08544 USA; ^9^ AI Graduate School Gwangju Institute of Science and Technology Gwangju 61005 Republic of Korea; ^10^ Department of Semiconductor Engineering Gwangju Institute of Science and Technology Gwangju 61005 Republic of Korea; ^11^ Department of Mechanical Engineering Massachusetts Institute of Technology Cambridge MA 02139 USA

**Keywords:** hydrogel thermostat, hygroscopicity, passive radiative cooling, *Populus alba*–inspired thermal regulation, thermochromism

## Abstract

Plants such as *Populus alba* feature photoprotective foliage that dynamically modulates optical properties to dissipate excessive heat under high temperatures, while condensation‐induced latent heating preserves warmth under cold conditions—enabling tolerance to fluctuating thermal and hydric environments. Inspired by this natural strategy, a hydrogel‐based thermostat is presented that balances latent and radiative heat fluxes. The system integrates lithium ions and hydroxypropyl cellulose into a polyacrylamide matrix, providing dynamic solar reflectance, high infrared emissivity, and reversible water sorption–desorption capabilities. Thermochromic and hygroscopic responses are tunable via adjustments in hydroxypropyl cellulose and lithium ions concentrations, allowing environment‐specific adaptation. Mechanical robustness is enhanced by incorporating titanium dioxide nanoparticles and applying surface treatments. Experiments and simulations demonstrate both sub‐ambient cooling and above‐ambient heating across diverse conditions, establishing the system as an all‐season thermal regulation platform.

## Graphical abstract

Guided by *Populus alba* foliage, this hydrogel latent‐radiative thermostat (LRT) actively balances latent and radiative heat fluxes. An LRT switches solar reflectance, maintains high mid‐IR emissivity, and reversibly evaporates/regenerates water, while titanium oxide (TiO_2_) nanoparticles toughen the film. The material yields sub‐ambient cooling and above‐ambient heating, enabling robust, all‐season thermal regulation.

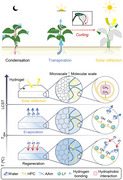

## 1 Introduction

**Figure 1:**
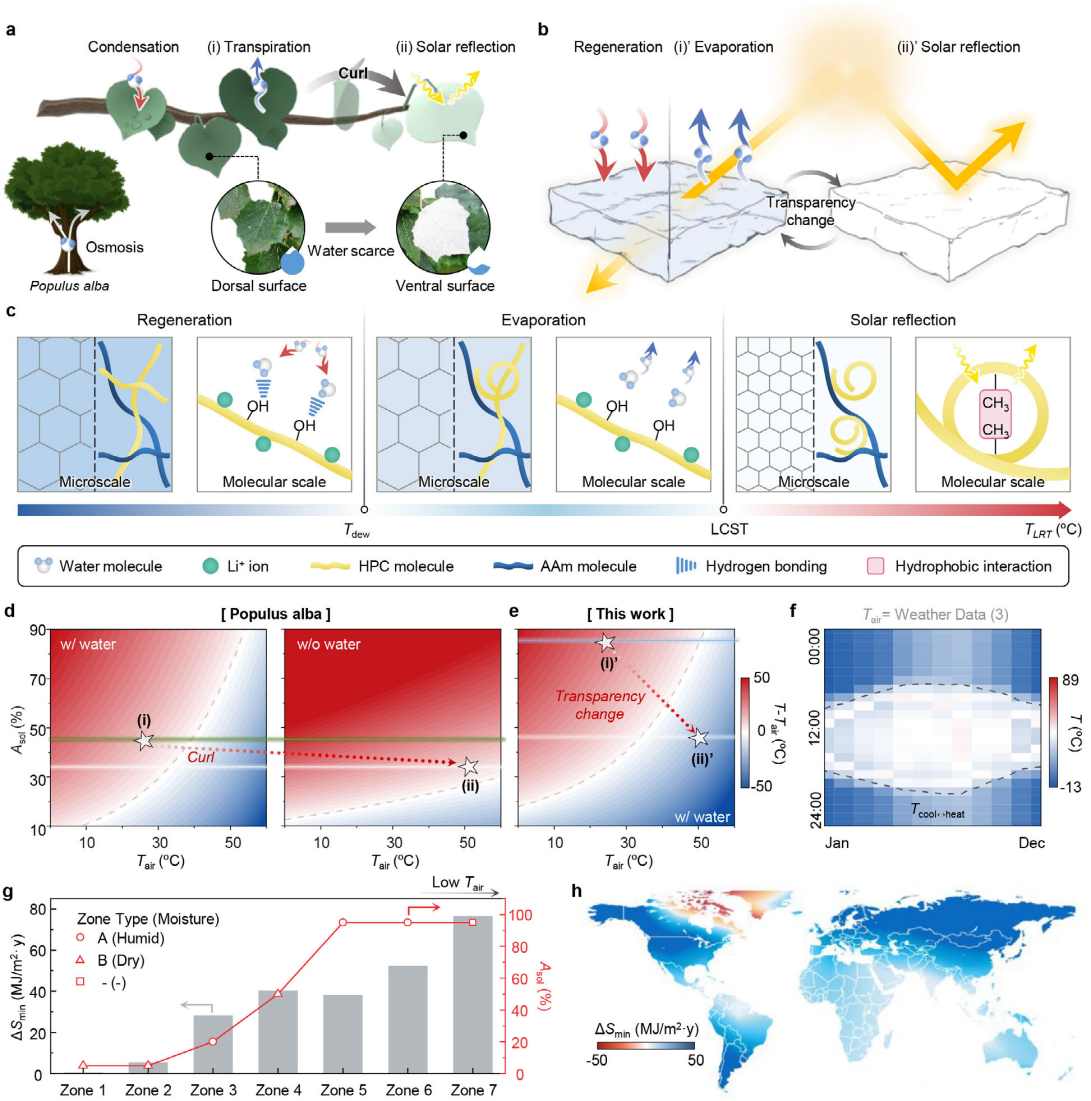
Concept and performance of the *Populus alba*‐inspired latent‐radiative thermostat (LRT). a) Schematic of *Populus alba* leaf thermoregulation: condensation‐induced latent heating; i) transpiration under water‐abundant conditions; and ii) solar reflection through leaf curling and ventral hair exposure under water‐scarce conditions. b) Working mechanism of the LRT mimicking the natural strategy: regeneration, i)’ evaporation, and ii)’ solar reflection. c) Microscale and molecular scale mechanisms underlying temperature‐dependent responses. At *T*_LRT_ < *T*_dew_, moisture adsorption induces latent heating. At *T*_dew_ < *T*_LRT_ < LCST, evaporation induces cooling. At *T*_LRT_ > LCST, transparency change enhances solar reflection. d,e) Simulated temperature maps for d) *Populus alba* leaves and e) LRT as functions of solar absorption (*A*_sol_) and ambient air temperature (*T*_air_), showing sub‐ambient cooling (blue region) and above‐ambient heating (red region), with and without water. (i), (ii), (i)’, and (ii)’ indicate thermal regulatory strategies of *Populus alba* and LRT in (a) and (b). f) Year‐round simulation of LRT's temperature under local weather data (Gwangju, Republic of Korea). g) Simulated space‐conditioning energy savings (Δ*S*_min_) of the LRT compared to RC and other roof coatings across seven climate zones with optimized *A*_sol_. h) Global map of Δ*S*_min_ of the LRT.

Plants continuously adapt to dynamic environmental conditions that vary across diurnal, seasonal, and climatic timescales. Light intensity, temperature, and water availability collectively govern the physiological and morphological responses of plant species. In most foliar plants, osmotic water uptake facilitates transpiration and enables daytime evaporative cooling, enhancing photosynthetic efficiency. Conversely, nocturnal condensation of atmospheric moisture on leaf surfaces passively releases latent heat, slightly elevating surface temperature and mitigating cold stress.^[^ ^1^, ^2^, ^3^ ^]^ This passive thermal management is particularly relevant to plant species adapted to variable climates. The genus *Populus*, widely distributed across temperate and boreal regions,^[^ ^4^ ^]^ exhibits exceptional tolerance to both water and thermal stress, making it a compelling model for bioinspired thermal regulation strategies. A well‐characterized example, *Populus alba* (white poplar), exhibits pronounced optical asymmetry between dorsal and ventral leaf surfaces. Under water‐limited and high‐temperature conditions—when transpiration is suppressed—the leaves curl inward, exposing their hair‐covered white ventral surfaces.^[^ ^5^ ^]^ This morphological adaptation enhances solar reflectance and mitigates overheating, serving as a photoprotective strategy to prevent photodamage under environmental stress (**Figure** **1**a; Figure S1, Supporting Information).^[^ ^5^, ^6^ ^]^ Despite the advantage of such photoprotective foliage, including spectral modulation and water responsiveness, this natural strategy has rarely been replicated in artificial systems.

Previous efforts in passive thermal regulation have focused on thermochromic or phase change materials with tunable optical properties. However, these approaches typically allow only independent, unidirectional thermal control—operating exclusively in either cooling or heating mode—and often exhibit diminished performance under humid or fluctuating weather conditions (Table S1, Supporting Information).^[^ ^7^, ^8^, ^9^, ^10^, ^11^, ^12^, ^13^, ^14^, ^15^, ^16^, ^17^, ^18^, ^19^, ^20^, ^21^, ^22^, ^23^ ^]^ A major limitation of these strategies is the absence of balanced coupling between radiative and latent heat regulation, a hallmark of thermal management in biological systems. To address this challenge, alternative platforms capable of multimodal and adaptive thermal regulation are needed. Functional hydrogels, comprising 3D hydrophilic polymer networks with functional additives, have emerged as versatile materials for applications in bioelectronics,^[^ ^24^, ^25^, ^26^, ^27^ ^]^ soft robotics,^[^ ^28^, ^29^, ^30^, ^31^ ^]^ and thermal management.^[^ ^32^, ^33^ ^]^ However, their potential for autonomous and truly bidirectional thermal regulation has remained largely unexplored.

Here, we present a photoprotective foliage‐inspired passive hydrogel thermostat—termed the latent‐radiative thermostat (LRT)—that synergistically regulates both latent and radiative heat transfer (Figures S2, S3, and Note S1, Supporting Information). By embedding lithium ions (Li⁺) and hydroxypropyl cellulose (HPC) into a polyacrylamide (PAAm) hydrogel matrix, the LRT dynamically responds to environmental stimuli—including ambient temperature, humidity, and solar irradiation—while enabling regeneration, evaporative cooling, and solar reflection (Figure 1b). Unlike conventional radiative coolers (RCs), the LRT achieves reliable temperature regulation even under humid conditions by balancing latent and radiative heat fluxes (Figure S4, Supporting Information).^[^ ^23^ ^]^ Simulations and experiments demonstrate that the LRT enables adaptive thermal control with improved energy efficiency across both diurnal and seasonal climate variations. The hydrogel composition—particularly HPC and Li⁺ concentrations—can be tailored to accommodate various environmental conditions, from arid to humid and from hot to cold climates. To enhance mechanical robustness and substrate adhesion, titanium dioxide (TiO_2_) nanoparticles and surface treatments are incorporated. Long‐term outdoor testing confirms the durability and functional versatility of the LRT for both surface‐level and enclosed‐space applications. As the first hydrogel‐based thermostat integrating latent and radiative heat control, this system offers a sustainable and climate‐adaptive solution for energy‐efficient thermal management—drawing direct inspiration from the integrated adaptability of natural foliage.

## 2 Results and Discussion

### 2.1 *Populus alba*‐Inspired LRT

Figure 1c illustrates the micro‐ and molecular‐scale mechanisms of the LRT, emulating the thermoregulatory adaptation of *Populus alba*. The LRT comprises a Li‐HPC‐PAAm hydrogel, where HPC serves as a thermochromic component while Li⁺ functions as a hygroscopic agent. These components enable autonomous thermal regulation in response to temperature, humidity, and solar radiation. The LRT operates via three distinct mechanisms—regeneration, evaporation, and solar reflection—depending on two critical temperature thresholds: dew point temperature (*T*_dew_) and lower critical solution temperature (LCST). When the temperature of the LRT (*T*_LRT_) is below *T*_dew_, it adsorbs atmospheric moisture, releasing latent heat.^[^ ^11^, ^33^, ^34^ ^]^ As *T*_LRT_ rises between *T*_dew_ and LCST, water evaporates from the LRT, leading to evaporative cooling. When *T*_LRT_ exceeds LCST, HPC molecules aggregate through hydrophobic interactions between methyl groups, increasing solar reflectance, and thereby improving cooling efficiency.^[^ ^7^, ^8^, ^14^, ^35^ ^]^

To evaluate the thermal performance of the LRT, we developed a coupled radiative and latent heat transfer model (Note S2, Supporting Information).^[^ ^23^, ^36^, ^37^, ^38^, ^39^, ^40^, ^41^ ^]^ To prevent overcooling or overheating, the RC and LRT were calibrated to achieve optimal solar reflectance (*R*_sol_), ensuring the temperatures of the systems stay within the human thermal comfort range of 25.5–30.9 °C (Notes S3 and S4, Supporting Information).^[^ ^42^ ^]^ Daytime simulations revealed that, while temperature fluctuations of the RC range from 14 to 51.8 °C, the LRT maintains a significantly narrower operational window of 25.6–33.6 °C (Figure S4a, Supporting Information). At night, the LRT maintains higher temperatures than the RC due to latent heating, enabling consistent thermal moderation (Figure S4j, Supporting Information).

Water responsiveness was evaluated by comparing surface temperatures of *Populus alba* and the LRT under both wet and dry conditions (Figure 1d,e). Solar absorption (*A*_sol_) primarily determines whether the system undergoes cooling or heating, regardless of water accessibility. Under wet conditions, the transition curve adopts an exponential shape, indicating thermal stability against changes in *A*_sol_ (Figure 1d, left). In contrast, under dry conditions, the transition becomes more linear, leading to thermally unstable behavior in response to fluctuating *A*_sol_ (Figure 1d, right). For example, when ambient air temperature (*T*_air_) is 50 °C, the leaf temperature reaches 61.6 °C, threatening biological viability. However, the LRT maintains a significantly lower temperature around 38.4 °C (Figure 1e). Moreover, the LRT autonomously modulates its *A*_sol_ from 85% to 44% when *T*_LRT_ exceeds 40 °C, reinforcing self‐regulation ability under thermal stress. In contrast to *Populus alba*, the LRT maintains its target temperature via two mechanisms: i) LiBr‐mediated water encapsulation suppresses unnecessary evaporation across ≈10–40 °C, delaying large *P*_evp_ and preventing overcooling when ambient heating is limited (Figure S2 and Note S1, Supporting Information). ii) Keeping *A*_sol_ within an appropriate, non‐excessive range provides a modest, water‐independent solar heating, keeping the LRT temperature within 25.6–33.6 °C for *T*_air_ = 0–60 °C—whereas *A*_sol_ = 0% (or *R*_sol_ = 0%) settles between −17 and 5.4 °C (Figure S4a, Supporting Information). Together, water encapsulation and *A*_sol_ moderation stabilize performance even above the *T*_dew_, where condensation is absent.

Annual simulations using historical weather data from Gwangju, Republic of Korea (35.17°N, 126.88°E), confirmed the system's adaptability (Figure 1f; Note S5 and Figure S6, Supporting Information).^[^ ^43^, ^44^ ^]^ Using a thermal comfort threshold (*T*_cool↔heat_ = 23 °C), the LRT achieved daytime cooling from March to October and consistent nighttime heating throughout the entire season, outperforming conventional RCs and the other roofing materials (Figure S6, Supporting Information). Additionally, the energy‐saving potential was evaluated across climate zones using a standard building model and historical weather data (Figures S7–S9, Supporting Information).^[^ ^43^, ^44^ ^]^ The LRT yielded the highest energy savings (Δ*S*_min_) compared to the four reference materials across seven global climate zones defined by the American Society of Heating, Refrigerating, and Air‐Conditioning Engineers (ASHRAE), with notable performance in colder zones such as Zone 7, where heating energy savings reached up to 76.5 MJ (m^2^·y)^−1^ (Figure 1g). This emphasizes the importance of controlling evaporation under low *T*_air_ environments.^[^ ^45^ ^]^ Global‐scale simulations confirmed broad applicability (Figure 1h). The LRT outperformed RCs and the other roofing materials in most major cities worldwide, except for extremely cold regions where limited solar irradiance reduces heating potential. Nonetheless, Δ*S*_min_ of the LRT reaches up to 153.94 MJ (m^2^·y)^−1^ (Tables S2 and S3, Supporting Information). These results highlight the LRT's capacity for passive, climate‐adaptive temperature regulation across diverse geographic regions.

### 2.2 Characterizations of LRT

**Figure 2:**
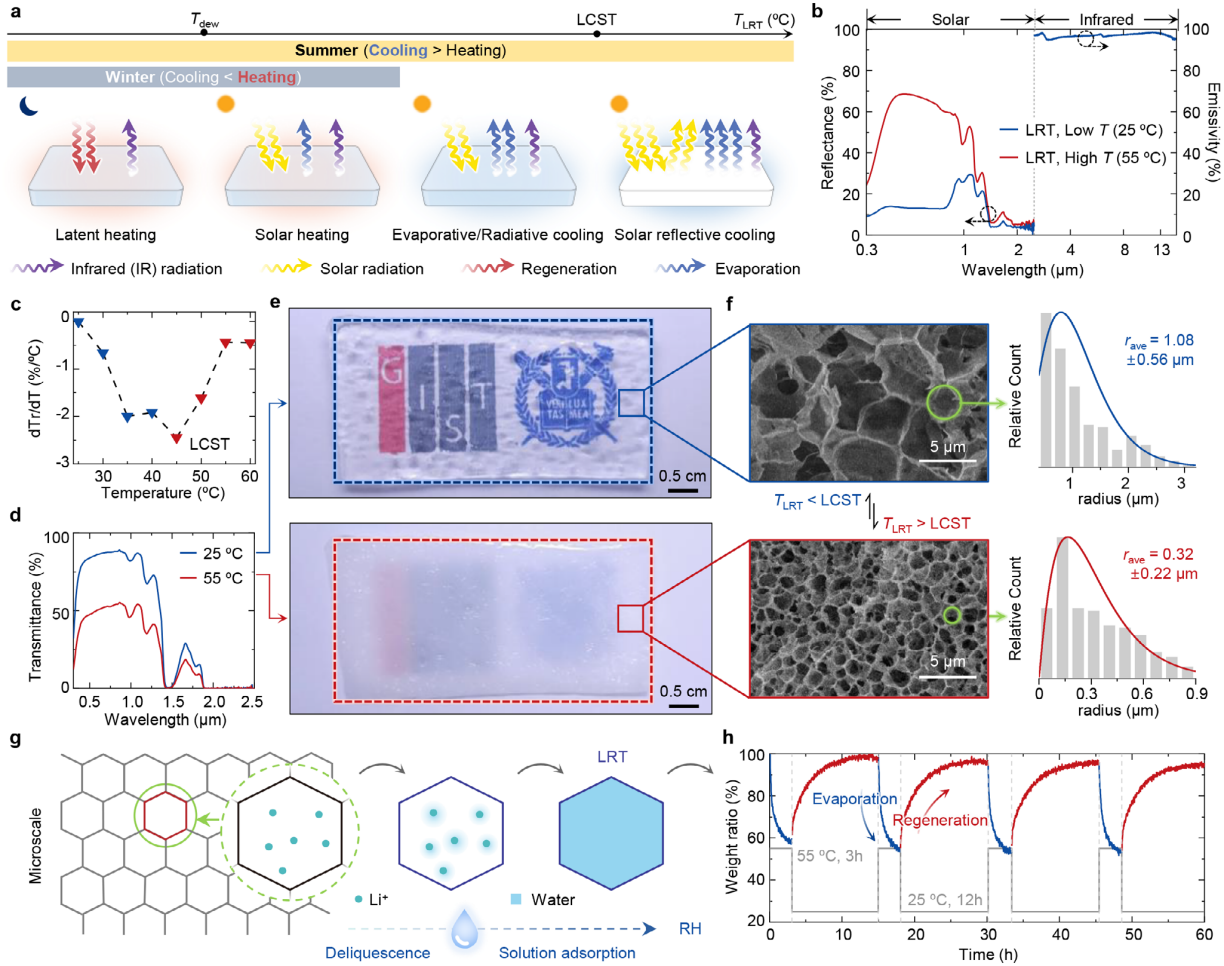
Optical modulation and moisture‐regulating properties of the LRT hydrogel. a) Schematic illustration of seasonal and diurnal thermal management of the LRT. The LRT autonomously switches between latent heating, solar heating, evaporative/radiative cooling, solar reflective cooling, enabling passive thermal management across winter/summer and day/night conditions. b) Measured reflectance and emissivity spectra of LRT from 0.3 to 16 µm at 25 °C (blue) and 55 °C (red), showing high infrared (IR) emissivity and dynamic solar reflectance. c) First derivative of the transmittance–temperature (Tr–T) curve, revealing an LCST around 45 °C. d) Transmittance spectra in the visible to near‐infrared range before and after the LCST, demonstrating temperature‐dependent optical modulation. e) Photographs of LRT in its transparent (25 °C, top) and opaque (55 °C, bottom) states due to thermochromism. f) SEM images and corresponding pore size distributions of the HPC‐AAm hydrogel below (top) and above (bottom) LCST. Pore size changes of micro‐to‐nano scale enhances light scattering at elevated temperatures. g) Schematic of the LRT's moisture regeneration process via LiBr deliquescence and subsequent solution adsorption. h) Measured cyclic sorption–desorption performance under 70% RH, showing stable 3‐h evaporation at 55 °C and 12‐h regeneration at 25 °C across multiple cycles.

**Figure** **2**a illustrates seasonal and diurnal heat transfer mechanisms of the LRT. During nighttime, the LRT adsorbs atmospheric moisture regardless of the season, releasing latent heat. Under daytime conditions (typically *T*_LRT_ > *T*_dew_), solar heating occurs due to near‐infrared absorption by water within the LRT. In summer, the evaporation counteracts and exceeds solar absorption, leading to combined evaporative and radiative cooling. As *T*_LRT_ rises above LCST, a change in transparency enhances solar reflectance, maximizing cooling efficiency.

Figure 2b shows the *R*_sol_ and infrared emissivity (*ε*_IR_) of the LRT below and above LCST. The LRT exhibits a static *ε*_IR_ of 95.4% in the atmospheric window, ensuring effective radiative cooling. Notably, *R*_sol_ of the LRT varies dynamically from 55.7% (above LCST) to 14.8% (below LCST), respectively (detailed measurement setup in Figure S10 in the Supporting Information). LCST is identified at ≈45 °C from the derivative of its transmittance‐temperature (dTr/dT) curve (Figure 2c).^[^ ^46^ ^]^ This relatively high LCST compared to other thermoresponsive polymers, such as poly(N‐isopropylacrylamide) (PNIPAM) enables clear differentiation between evaporative and combined cooling performances in the LRT.^[^ ^47^ ^]^ The LRT exhibits 76.6% of solar transmittance (*T*_sol_) and 84.8% of visible transmittance (*T*_vis_) at 25 °C (Figure 2d). As the temperature increases to 55 °C, *T*_sol_ and *T*_vis_ significantly decrease to 44.4% and 49.1%, respectively. Additionally, such optical modulation behavior remains stable without noticeable degradation even after aging tests under high‐temperature/high‐humidity conditions and repeated freeze–thaw cycles (Figure S10f,g, Supporting Information). Visual confirmation of this transition is provided in Figure 2e and Figure S11 (Supporting Information), further confirming that the optical modulation behavior remains stable and humidity‐independent. Scanning electron microscopy (SEM) images reveal pore size change from ≈2.16 µm to ≈320 nm above LCST, enabling optical modulation (Figure 2f).

Beyond spectral tuning, the LRT leverages reversible latent heat exchange for thermal regulation. Among various hygroscopic salts, lithium bromide (LiBr) was selected as the adsorbent owing to its exceptionally low deliquescence relative humidity (DRH; ≈6–8% at 30 °C) and high moisture uptake capacity. These characteristics enable efficient moisture regeneration even under arid conditions, whereas other salts such as calcium chloride (CaCl_2_; DRH ≈28% at 30 °C) and magnesium chloride (MgCl_2_; DRH ≈33% at 30 °C) are considerably less suitable.^[^ ^48^ ^]^ Although zinc chloride (ZnCl_2_) exhibits a low DRH of ≈5–7% at 30 °C, its limited moisture sorption capacity with corrosive and toxic nature hinders practical applications.^[^ ^48^, ^49^ ^]^ By contrast, LiBr‐based hydrogel demonstrates stable and reversible sorption–desorption cycles, thereby broadening the effective humidity adaptability and ensuring reliable latent heat regulation.^[^ ^11^ ^]^ Below *T*_dew_, the LRT adsorbs atmospheric moisture through deliquescence and crystalline water formation, enabling latent heating (Figure 2g). Above *T*_dew_, the inverse process occurs, resulting in evaporative cooling.^[^ ^50^ ^]^ Repeatability and environmental stability were evaluated via cyclic regeneration‐evaporation tests in a relative humidity (RH)‐controlled chamber (Figure S12a–c, Supporting Information). The LRT exhibited an S‐shaped evaporation and regeneration isotherm with abrupt change around 60–80% RH, characteristic of capillary condensation in porous structures (Figure S12d, Supporting Information). Cyclic evaporation‐regeneration behavior highlights the stability of the LRT for repeated latent heating and evaporative cooling (Figure 2h). Furthermore, the material's hygroscopic nature enables more moisture retention under ambient conditions and allows regeneration even after long‐term exposure to low humidity (Figure S12e,f, Supporting Information). Also, the evaporable water inside LRT accounts for 62.9% of total weight, enabling latent heat‐driven regulation (Figure S12g, Supporting Information). The system exhibits temperature‐responsive optical modulation and spontaneous moisture evaporation–regeneration behavior, enabling dynamic and passive thermal regulation.

### 2.3 Potential for Practical Applications of LRT

**Figure 3:**
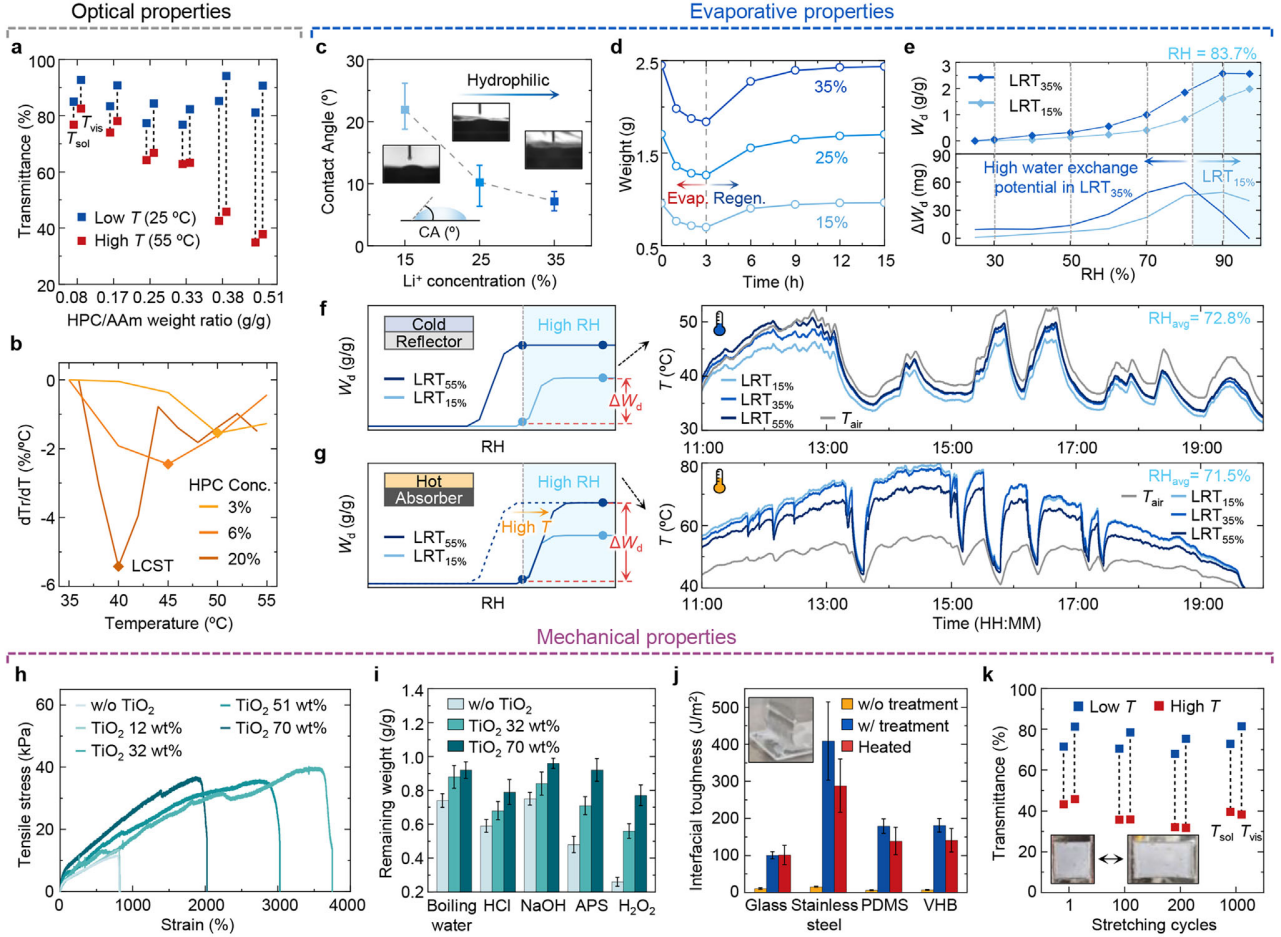
Tunability and mechanical robustness of LRT for practical application. a) Visible (*T*_vis_) and solar (*T*_sol_) transmittance of LRT at 25 °C (blue) and 55 °C (red) as a function of HPC/AAm weight ratio. b) LCST variation of LRT by HPC concentration. c) Water contact angle as a function of Li⁺ concentration. d) Mass variation of LRT with varying Li⁺ concentrations during heating (55 °C) and cooling (25 °C) cycles at 70% RH. e) Water vapor sorption isotherms (top) and derivatives (bottom) for 15% and 35% of Li^+^ concentration LRTs (LRT_15%_ and LRT_35%_, respectively). f,g) Schematic of evaporation behavior and measured surface temperature of the LRT with different Li⁺ concentration on (f) cold and (g) hot substrates under high‐humidity conditions. h) Tensile stress–strain curves of LRTs with different titanium dioxide (TiO_2_) concentrations. i) Weight retention after 3‐day exposure to boiling water, acid (HCl), base (NaOH), and oxidizers (H_2_O_2_, APS). j) Interfacial toughness of LRT on various substrates (glass, stainless steel, PDMS, VHB) under three conditions: without surface treatment (w/o treatment), after surface treatment (w/ treatment), and after thermal cycling (Heated). k) Stability of *T*_vis_ and *T*_sol_ after cyclic stretching.

Environmental adaptability and mechanical robustness are essential for the practical implementation of the LRT. A key requirement is the tunability of solar modulation and LCST. Increasing the HPC concentration enhances the transmittance difference between 25 and 55 °C (**Figure** **3**a; Figure S10f,g, Supporting Information). An HPC/AAm weight ratio of 0.33 was selected for outdoor demonstrations, yielding Δ*T*_sol_ of 14% and Δ*T*_vis_ of 19%. Higher HPC concentrations lower LCST to ≈40 °C (Figure 3b). Moreover, increasing the Li⁺ concentration allows additional tuning of the LCST (Figure S13, Supporting Information). Additionally, material‐level modification through methylcarbamate substitution expands the tunable range from 15 to 94 °C, allowing environment‐specific applications.^[^ ^51^ ^]^

Humidity‐responsive latent heat regulation was tuned by varying the Li⁺ concentration. Increasing the Li⁺ content from 15% to 35% reduces the contact angle from 22.0° to 7.2°, enhancing hydrophilicity and water exchange capacity (Figure 3c). Correspondingly, the weight loss during 3 h evaporation at 55 °C increased from 0.24 to 0.612 g, with complete regeneration within 12 h at 25 °C (Figure 3d; Figure S12h, Supporting Information). Water vapor sorption isotherms revealed that the LRT with 35% Li^+^ (LRT_35%_) exhibits superior moisture retention under 90% RH compared to the LRT with 15% Li^+^ (LRT_15%_) (Figure 3e). Notably, a crossover point between the LRT_15%_ and LRT_35%_ is observed at 83.7% RH, highlighting the importance of Li^+^ concentration tuning for humidity‐specific applications.

In addition to RH, target object temperature plays a crucial role in latent heat regulation. Differential scanning calorimetry shows that LRT_15%_ and LRT_35%_ exhibit peak evaporation at ≈90 and ≈125 °C, respectively, indicating the suitability of LRT_15%_ for cooler objects and LRT_35%_ for hotter ones (Figure S12i, Supporting Information). To emphasize performance difference under high‐humidity conditions, an LRT with 55% Li⁺ (LRT_55%_) was additionally evaluated (Figure 3f,g). On cold substrates with high solar reflectance (enhanced specular reflector, ESR), LRT_15%_ showed greater weight loss under high‐humidity conditions, while LRT_55%_ remained near equilibrium. In contrast, on hot substrates with high solar absorptance (black emitter), LRT_55%_ exhibited enhanced evaporative cooling even under high humidity conditions, owing to higher evaporation capacity. Field tests confirmed these trends: LRT_15%_ cooled by 3.6 °C under cold conditions (Figure 3f), whereas LRT_55%_ achieved a cooling temperature of 4.3 °C on hot substrates (Figure 3g). These results demonstrate that evaporative cooling is tunable with respect to both RH and substrate temperature (Figure S14 and Note S6, Supporting Information).

Mechanical resilience is vital for outdoor stability. Incorporating TiO_2_ nanoparticles into the LRT significantly enhances its mechanical strength (Figure 3h; Figure S15a, Supporting Information). At 32 wt.%, the elongation at break increases from 785% to 3428% and the tensile strength from 12 to 40 kPa due to hydrogen bonding between TiO_2_ and polymer chains (Figure S15b, Supporting Information).^[^ ^52^ ^]^ However, mechanical performance deteriorates beyond 32 wt.% TiO_2_ due to nanoparticle aggregation. TiO_2_‐rich LRTs also exhibit superior chemical resistance to boiling water, acid, base, and oxidizing environments (Figure 3i). The LRT with 70 wt.% TiO_2_ showed the highest weight retention, suggesting excellent environmental stability, and based on the well‐known UV shielding and self‐cleaning properties of TiO_2_, additional resistance to UV exposure and pollution is also expected. In addition to the improvements in mechanical and chemical resistance, increasing the TiO_2_ concentration also enhances the solar reflectance of LRTs, which exhibits effective cooling performance in regions with high cooling demand (Figure S15c,d, Supporting Information).

Adhesion to substrates was improved through surface modification: adhesion was improved through silane functionalization for glass and stainless steel and UV‐grafting for polydimethylsiloxane (PDMS) and very high bond tape (VHB) by 9‐ to 29‐fold and maintained over 100 J m^−^^2^ even after thermal cycling at 70 °C (Figure 3j; Figure S15e–h, Supporting Information).^[^ ^53^, ^54^ ^]^ Improved adhesion enhances thermal performance: surface‐treated LRTs showed a 0.3 °C lower cooling temperature than untreated ones, due to reduced delamination (Figures S16 and S17, Supporting Information). Optical stability under deformation was confirmed through 1000 stretching cycles at 50% strain (Figure 3k; Figure S18, Supporting Information). These results demonstrate that the LRT combines tunable optical and latent heat regulation with strong mechanical and environmental robustness, positioning it as a promising candidate for diverse applications across varying climates and operational conditions.

### 2.4 Thermostat Performance of LRT

**Figure 4:**
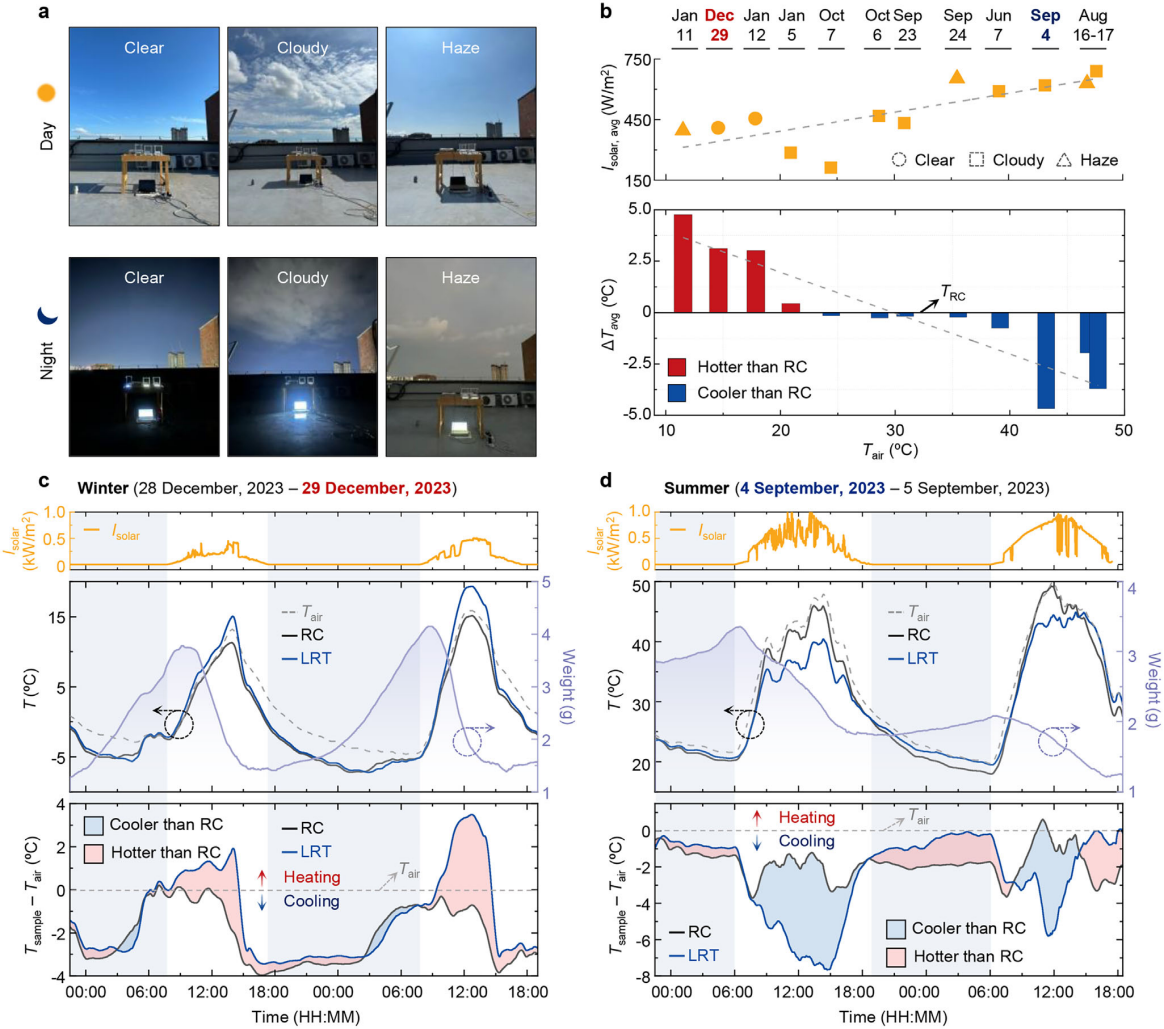
Adaptive thermostat performance of LRT under dynamic outdoor conditions. a) Photographs of clear, cloudy, and hazy sky conditions during day and night at the outdoor test setup in Gwangju, Republic of Korea. b) Average daytime surface temperature difference between LRT and RC (Δ*T*_avg_) as a function of ambient temperature (*T*_air_). LRT exhibits higher temperatures under cold conditions (*T*_air_ < 25 °C) and lower temperatures under hot conditions (*T*_air_ > 25 °C), confirming bidirectional thermal regulation. c) 42‐h continuous measurement in winter (28–29 December, 2023), showing that LRT remains up to 3.5 °C warmer than ambient air due to solar heating during daytime, while RC cools below *T*_air_. d) 42‐h measurement in summer (4–5 September, 2023), where LRT maintains up to 3.7 °C sub‐ambient cooling via combined radiative and evaporative mechanisms, outperforming RC. Pink and blue shaded regions indicate heating and cooling advantage of LRT relative to RC, respectively. These results highlight the LRT's ability to autonomously maintain temperatures near the thermal comfort zone across varying seasons and weather conditions.

To evaluate the real‐world thermal regulation performance of the LRT, we conducted outdoor temperature measurements using the setup shown in **Figure** **4**a. The RC (100 µm‐thick PDMS) and LRT were placed on ESR films, simulating metallic substrates (Figure S19, Supporting Information). Figure 4b shows average solar irradiance (*I*_solar, avg_, top) and surface temperature difference between the RC and LRT (Δ*T*_avg_, bottom) under clear, cloudy, and haze sky conditions (details provided in Figure S20 in the Supporting Information). Across all test conditions, the LRT exhibited superior adaptability to dynamic thermal environments, including variations in temperature and solar irradiance. During the daytime requiring heating (*T*_air_ < *T*_cool↔heat_), the LRT maintained higher surface temperatures than the RC due to solar heating. Conversely, under high‐temperature conditions (*T*_air_ > *T*_cool↔heat_), the LRT achieved superior cooling performance via radiative and evaporative cooling with enhanced solar reflection. These results confirm that the LRT autonomously adjusts its thermal behavior in response to varying external conditions—hot and cold, dry and humid.

Figure 4c,d presents continuous 42‐h measurements during winter and summer, respectively. In the winter daytime (*I*_solar, avg_ of 188 W m^−^^2^), the RC shows an undesired cooling effect even in cold environments where heating is required (Figure 4c). In contrast, the LRT reaches a maximum above‐ambient heating temperature of 3.5 °C owing to solar heating. In the summer daytime (*I*_solar, avg_ of 443 W m^−^^2^), the RC and LRT exhibit sub‐ambient cooling performance of 2.0 and 3.7 °C, respectively (Figure 4d). Notably, the LRT achieves a 1.7 °C lower surface temperature than the RC due to radiative and evaporative cooling effects with enhanced solar reflection. Furthermore, during nighttime, the LRT maintained higher temperatures than the RC due to latent heating. Comparative measurements using the LRT without HPC confirmed that the LRT significantly enhances the cooling effect owing to enhanced solar reflection (Note S7 and Figures S19–S21, Supporting Information). Nighttime measurements demonstrated the latent heating effect (Figure S22, Supporting Information). These results confirm that the LRT provides consistent and climate‐responsive thermal regulation across diverse environmental conditions, exhibiting stable performance within the *T*_air_ range of −10.3–57.0 °C.

### 2.5 LRT Dome Practical Application Demonstration

**Figure 5:**
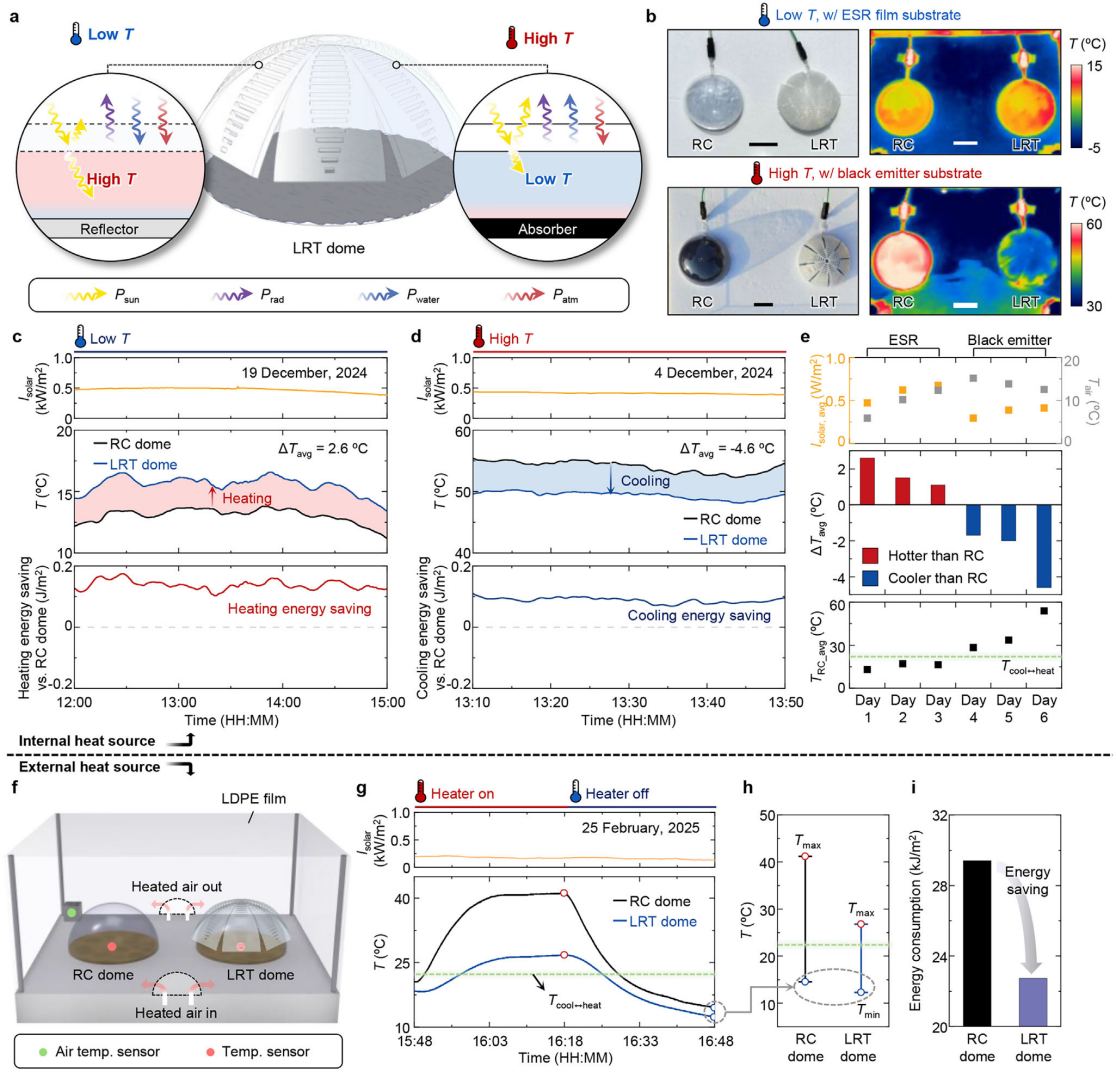
Thermal regulation performance of LRT dome structure under realistic and extreme conditions. a) Schematic of the LRT dome and corresponding heat transfer mechanisms, including absorbed solar power (*P*_sun_), emitted power (*P*_rad_), net evaporative power (*P*_water_), and atmospheric thermal radiation (*P*_atm_). Two substrate conditions are considered: reflector (cold) and absorber (hot). b) Optical (left) and infrared (right) images of LRT and RC domes at low and high substrate temperatures. The scale bar is 5 cm. c,d) Outdoor measurements of solar irradiance (top), internal dome temperature (middle), and estimated heating/cooling energy savings compared to RC dome (bottom) under (c) cold and (d) hot substrate conditions. e) Averaged temperature difference between LRT and RC domes over six days under varying substrates. f) Schematic of the experimental setup for testing response to cyclic hot air exposure, simulating an external heat source. g) Real‐time internal temperature profiles of RC and LRT domes under periodic heated air flow conditions. h) Comparison of maximum (*T*_max_) and minimum (*T*_min_) dome temperatures during the heating cycles. The green dashed line indicates the thermal comfort threshold (*T*_cool↔heat_ = 23 °C). i) Estimated energy required to maintain comfort range, with the LRT dome reducing energy demand by 6.7 kJ m^−^^2^ compared to RC.

To assess the real‐world applicability of the LRT in enclosed environments, we fabricated a dome‐shaped LRT structure (LRT dome) and evaluated its thermal regulation performance under both outdoor and controlled conditions (Figure S15i–l, Supporting Information). The LRT dome was placed on two types of substrates to evaluate responses under different internal heat sources: an ESR film simulating low‐temperature conditions and a black emitter representing high‐temperature scenarios. On cold substrates, the LRT's higher *A*_sol_ increased the roof surface temperature, inducing a moderate greenhouse effect and elevating the internal air temperature (**Figure** **5**a). On hot substrates, the LRT transitions to an opaque state and facilitates evaporative and radiative cooling with enhanced solar reflection, reducing both roof surface and internal air temperatures.^[^ ^55^ ^]^ Figure 5b shows visual and IR images of the RC and LRT domes under both substrate conditions. Both RC and LRT domes remain transparent in cold conditions, but the LRT dome shows a higher surface temperature due to solar absorption. In contrast, the LRT dome becomes opaque under hot conditions and exhibits a lower roof surface temperature than the RC dome. Corresponding temperature profiles are shown in Figure 5c,d. On cold substrates, the LRT dome maintains a higher average internal temperature of 2.6 °C than the RC dome, saving 1.5 kJ m^−^^2^ in heating energy to reach *T*_cool↔heat_. Conversely, on hot substrates, the LRT dome shows a lower internal temperature of 4.6 °C than the RC dome, resulting in a cooling energy saving of 0.2 kJ m^−^^2^. Repeated trials confirm the reproducibility of these results (Figure 5e; Figure S23, Supporting Information). These results highlight the LRT's potential as a self‐adjusting, geometry‐conformal envelope capable of regulating temperature across a range of internal thermal conditions.

To investigate the thermal regulatory performance under external heat input, we constructed a test chamber with cyclic hot airflow (Figure 5f). The LRT dome exhibited more stable internal temperature fluctuations, remaining closer to *T*_cool↔heat_, while the RC dome responded more sensitively to external heat sources (Figure 5g,h). During heated periods, the LRT dome maintained a maximum temperature (*T*_max_) that was 14.4 °C lower than that of the RC dome. Once heating was halted, the RC dome exhibited rapid cooling, eventually reaching a minimum temperature (*T*_min_) comparable to that of the LRT dome. Based on these profiles, the estimated energy consumption required for each dome to reach *T*_cool↔heat_ is presented in Figure 5i. The LRT dome achieved a total energy saving of 6.7 kJ m^−^^2^ compared to the RC dome, highlighting its potential for energy‐efficient temperature regulation and climate‐adaptive applications in enclosed environments.

## 3 Conclusion

In this study, we introduce a passive thermostat inspired by the photoprotective strategy of *Populus alba*, integrating latent and radiative heat flux modulation through a hydrogel‐based platform to maintain adaptive thermal balance. The LRT leverages three synergistic properties—high IR emissivity, dynamic solar modulation, and latent heat regulation—to ensure thermal stability under diverse and fluctuating environmental conditions.

Our results demonstrate that the LRT can dynamically adjust *A*_sol_, enabling smooth transitions between heating and cooling states—analogous to the thermal homeostasis observed in *Populus alba*. Extensive outdoor experiments and computational simulations confirm that the LRT effectively stabilizes temperature across various climates, outperforming conventional passive coolers with fixed spectral properties or limited hygroscopic response.

Material‐level tunability further enhances the versatility of the system. By adjusting the HPC and Li⁺ concentrations, the LRT's optical and hygroscopic properties can be tailored to specific climate zones—from arid to humid, hot to cold. Specifically, in hot‐humid and arid climates, its combined radiative and latent heat regulation ensures stable cooling performance. Furthermore, in cold or fluctuating environments, moisture adsorption and solar absorption provide an effective heating effect. In parallel, mechanical durability is enhanced through TiO_2_ incorporation and surface treatment strategies, ensuring long‐term operational robustness under real‐world conditions.

The LRT combines passive adaptability, bidirectional thermal control, and geometrical flexibility, distinguishing it from existing passive heat‐management technologies. Its ability to regulate temperature across both open and enclosed environments enables scalable applications, such as building‐integrated systems. Specifically, owing to its ability to autonomously switch between cooling and heating modes depending on environmental conditions, the LRT is highly suitable for practical applications such as building envelopes, roofing systems, and temporary shelters. By emulating nature's hierarchical and adaptive thermoregulatory mechanisms, it establishes a foundational platform for multimodal, self‐regulating systems that respond not only to temperature but also to broader environmental cues such as humidity and solar radiance—paving the way toward sustainable thermal comfort and energy‐efficient technologies.

## 4 Experimental Section

### Materials

Hydroxypropyl cellulose (HPC; 191892, Sigma–Aldrich, USA) was used as the thermoresponsive polymer for hydrogel fabrication. Acrylamide (AAm; A8887, Sigma–Aldrich) served as the main polymer matrix, and N,N′‐methylenbisacrylamide (BIS; 146072, Sigma–Aldrich) was used as the chemical cross‐linker. Ammonium persulfate (APS; A3678, Sigma–Aldrich) and N,N,N′,N′‐tetramethylethylenediamine (TMEDA; T9281, Sigma–Aldrich) were used as the free‐radical initiator and accelerator, respectively. Lithium bromide (LiBr; 213225, Sigma–Aldrich) was incorporated to provide hygroscopic functionality and improve moisture regeneration behavior.

### Fabrication of LRT

To prepare the LRT, a precursor solution was prepared by dissolving AAm (1.56 g), BIS (1 mg), and HPC (0.13–0.8 g) in 10 mL of deionized (DI) water, corresponding to an HPC‐to‐AAm weight ratio ranging from 0.08 to 0.51 (g/g) (Figure S24, Supporting Information). The mixture was stirred at 350 rpm for 4 h at room temperature to ensure complete dissolution. The solution was then degassed in a vacuum chamber overnight to eliminate residual air bubbles that could interfere with uniform polymerization. Polymerization was initiated by adding 100 µL of 10 wt.% APS aqueous solution and 100 µL of 10 wt.% TMEDA aqueous solution to the degassed solution. The mixture was stirred for 1 min at 250 rpm and immediately poured into customized molds before gelation. The resulting hydrogels were dried in a convection oven at 70 °C until completely dehydrated.

Dried samples were soaked in a 35 wt.% LiBr aqueous solution for at least 24 h until fully swollen and equilibrated. To enhance surface adhesion, glass and stainless steel substrates underwent silane functionalization. The surfaces were first treated with oxygen plasma (RIE; 100 sccm O_2_, 0.2 Torr, 30 W, 5 min), followed by immersion in an aqueous silane solution consisting of 2 mL of 3‐(trimethoxysilyl)propyl methacrylate (TMSPMA; M6514, Sigma–Aldrich) and 100 mL of DI water containing 10 µL of acetic acid. Surface treatment was performed for 2 h, followed by thorough rinsing with ethanol and DI water. Polydimethylsiloxane (PDMS) and very high bond tape (VHB) substrates were treated via UV‐activated grafting. A 10 wt.% benzophenone (B9300, Sigma–Aldrich) solution in ethanol was applied to the surface for 3 min, followed by rinsing with ethanol and DI water. Subsequently, the hydrogel precursor solution was applied and cured on the treated surface to enable robust bonding.

### Incorporation of TiO_2_ Nanoparticles and Environmental Resilience Test

To prepare TiO_2_‐containing hydrogels, AAm (1.56 g), HPC (0.51 g), and BIS (1 mg) were dissolved in 10 mL of DI water. TiO_2_ nanoparticles (AV‐3548, AVENTION; average diameter ≈500 nm) were then added at 0.2, 0.5, 0.8, or 1.1 g to achieve TiO_2_‐to‐AAm weight ratios of 12, 32, 51, and 70 wt.%, respectively. Polymerization was conducted using the same procedure as for the LRTs.

For environmental resilience testing, hydrogels without TiO_2_ and with 32 and 70 wt.% TiO_2_ were prepared in 1 cm × 1 cm × 2 mm dimensions. Samples were dried in a convection oven at 70 °C for 24 h, and their dry weights were recorded. The dried samples were then immersed in different chemical environments (boiling water, 1 m HCl, 1 m NaOH, 0.1 m APS, or 10 wt.% H_2_O_2_) for 3 days. After exposure, the samples were redried and weighed to assess weight retention.

### Microstructural Characterization

Scanning electron microscopy (SEM; S‐4700, Hitachi Hi‐Tech) was used to visualize the porous structure of the HPC‐PAAm hydrogel. Prior to imaging, the hydrogel was washed in DI water to remove residual Li⁺ and Br^−^ ions, minimizing contrast interference. Dried samples were sputter‐coated with Pt for 60 s. Pore size distributions were analyzed using Image J software under consistent auto‐thresholding settings.

### Optical and Mechanical Characterization

The LCST of LRT was determined by monitoring the derivative of the transmittance–temperature (Tr–T) curve at 550 nm, using a 1 °C min^−1^ heating rate and a 5‐min equilibration step at each interval. Spectral characterization in the 0.3–2.5 µm range was performed using a UV–vis–NIR spectrometer with integrating sphere (Lambda 750, PerkinElmer). Samples were mounted on a temperature‐controlled stage allowing both heating and cooling. IR emissivity (*ε*_IR_; 2.5–16 µm) was obtained using a Fourier‐transform infrared spectrometer (VERTEX 70v, Bruker) with an Au‐coated integrating sphere. Emissivity (*E*) was calculated from measured reflectance (*R*) and transmittance (*T*) using *E* = 100 – *R* – *T*.

Mechanical properties were characterized using a universal testing machine (34SC‐1, Instron). Hydrogel samples were cut into a dog‐bone shape (10 mm width, 10 mm gauge length, 2 mm thickness) and stretched at a constant rate of 10 mm min^−1^ until fracture. Tensile strength and elongation at break were extracted from stress–strain curves.

For adhesion measurement, slide glass substrates (2.5 cm × 7.5 cm) were surface‐treated as described above. A customized polyethylene terephthalate (PET) frame (2 mm thickness) was placed on top of the substrate, and the hydrogel precursor solution was dropped into the mold. Stainless‐steel gauze (type 316, Goodfellow), functionalized with TMSPMA, was laminated onto a PET film and used as the backing layer. The extended end of the gauze was clamped to the crosshead of a universal testing machine (ESM301, Mark‐10). The bottom of the glass was fixed to a 90° peel test fixture (G1045, Mark‐10) using double‐sided adhesive tape (VHB, 3 M). The peeling tests were performed at a constant rate of 50 mm min^−1^.

For the cyclic stretching test, VHB substrates were surface‐treated as described above. A customized PET frame (0.5 mm thick) was placed on the substrate, and the hydrogel precursor solution was poured into the mold to form a square‐shaped hydrogel (3 cm × 3 cm). The prepared sample was mounted onto a uniaxial tensile stage (Jaeil Optical System). Cyclic stretching was performed between 0% and 50% strain for 1, 100, 200, and 1000 cycles.

### Moisture Regeneration and Evaporation Characterization

The moisture regeneration and evaporation capabilities were evaluated by measuring real‐time weight changes under controlled humidity and temperature. A high‐precision electronic balance (ML‐T, Mettler Toledo) was coupled with a thin‐film resistive heater (KHLVA‐202/5‐P, Omega Engineering), controlled by a DC power supply (TPT20‐10, Toyotech). Weight data were recorded using EasyDirect software (Mettler Toledo).

Contact angles were measured using 5 µL water droplets on each sample with a video‐based optical system (Phoenix 300 Touch, SEO Inc.). For each sample, five contact angles were measured and averaged. Water vapor sorption isotherms were determined by measuring equilibrium mass under stepwise RH conditions at 25 °C.

### Fabrication of LRT Dome

A PDMS sheet (20 cm × 20 cm, 0.2 mm thick) was prepared and surface‐treated as described above. A customized PET frame (0.5 mm thick) was placed on the PDMS surface, and the hydrogel precursor solution was cast into the mold to form a square hydrogel (20 cm × 20 cm). A kirigami pattern was introduced via laser cutting, allowing the flat 2D sheet to be transformed into a 3D structure. The LRT was applied to a PET‐based dome (radius ≈5.6 cm) to fabricate the dome architecture.

### Outdoor Thermal Regulation Measurement

Thermal measurements were conducted in Gwangju, Republic of Korea. Samples were mounted on XPS foam blocks with thermally insulating properties. For roofing simulation, ESR film or black emitter film was placed under each sample. Temperature sensors (ST‐50, RKC Instrument Inc.) were embedded between the substrate and foam block and connected to a 6‐channel data logger (RDXL6SD, Omega Engineering).

The setup was enclosed in a transparent acrylic chamber covered with perforated low density polyethylene (LDPE) film to allow vapor exchange while minimizing wind effects. Ambient air temperature was monitored using a thermocouple placed inside an aluminum foil‐coated cardboard box to shield from direct sunlight. Solar irradiance was measured using a pyranometer (CMP6, Kipp & Zonen) positioned adjacent to the sample platform.

To evaluate thermal regulatory performance under an external heat source, a custom test chamber was constructed with cyclic hot airflow. The chamber was enclosed with LDPE film to minimize interference from ambient solar and infrared radiation. Heated air was periodically introduced through an inlet to simulate external thermal disturbances.

## Conflict of Interest

The authors declare no conflict of interest.

## Author Contributions

S.‐Y.H., H.R.K., and Y.S. contributed equally to this work. S.‐Y.H., H.R.K., Y.S., D.‐H.K., and Y.M.S. conceived the idea and designed the whole experiment. S.‐Y.H., H.R.K., and Y.S., developed the process and fabricated the samples. H.S.L. and H.K. helped with the fabrication. S.‐Y.H. performed the theoretical modeling. D.H.K. and D.H.S. assisted with the theoretical calculations. S.‐Y.H., H.R.K., Y.S., H.S.L., and H. K. conceived the experiments and analysed the data. S.‐Y.H., H.R.K., Y.S., H.E.J., and S.C. visualized the data. J.H.K., M.S.K., L.L., J.M., W.L., D.‐H.K., and Y.M.S. validated the simulation results with experimental data. S.‐Y.H., H.R.K., Y.S., D.‐H.K., and Y.M.S. wrote the manuscript. D.‐H.K. and Y.M.S. edited the manuscript. D.‐H.K. and Y.M.S. guided the entire project. All authors discussed experimental and numerical results and edited the paper.

## Supporting information



Supporting Information

## Data Availability

The data that support the findings of this study are available in the supplementary material of this article.
