# Supplementary material for: Hydrogel Thermostat Inspired by Photoprotective Foliage Using Latent and Radiative Heat Control
Source: Adv Mater. 2025 Nov 4;38(17):e16537. doi: 10.1002/adma.202516537 (PMC13003909; doi:10.1002/adma.202516537)
Supplement: Supplementary file 1 — Supporting Information [file ADMA-38-e16537-s001.pdf]

Supporting Information

Hydrogel Thermostat Inspired by Photoprotective Foliage Using Latent and Radiative Heat Control

Se-Yeon Heo, Hyung Rae Kim, Yoonsoo Shin, Hyun Su Lee, Hyunkyu Kwak, Do Hyeon Kim, Dong Hyun Seo, Joo Hwan Ko, Hyo Eun Jeong, Sehui Chang, Min Seok Kim, Longnan Li, Jyotirmoy Mandal, Wei Li, Dae-Hyeong Kim, Young Min Song**

The PDF file includes:

Nomenclature
Supplementary Notes 1 to 6
Supplementary Figures 1 to 26
Supplementary Tables 1 to 3

Nomenclature

Abbreviations

AAm	acrylamide
AgNW	silver nanowire
Al_2O_3	aluminum oxide
APS	ammonium persulfate
ASHRAE	American Society of Heating, Refrigerating and Air-Conditioning Engineers
BIS	N,N′-methylenbisacrylamide
CA	cellulose acetate
$CaCl_2$	calcium chloride
CM	chameleon microcapsules
DI	deionized
DRH	deliquescence relative humidity
ESR	enhanced specular reflector
H_2O_2	hydrogen peroxide
HCl	hydrochloric acid
HfO_2	hafnium dioxide
HPC	hydroxypropyl cellulose
LCST	lower critical solution temperature
LDPE	low density polyethylene
Le	Lewis number
LiBr	lithium bromide
Li^+	lithium ion
NaOH	sodium hydroxide
P(VDF-HFP)	poly(vinylidene fluoride-co-hexafluoropropylene)
PDMS	polydimethylsiloxane
PAN	polyacrylonitrile
PAAm	polyacrylamide
PET	polyethylene terephthalate
PNIPAM	poly(N-isopropylacrylamide)
RH	relative humidity
RC	radiative cooler
LRT	latent-radiative thermostat

$MgCl_2$	magnesium chloride
SEM	scanning electron microscope
SiO_2	silicon dioxide
TMEDA	N,N,N′,N′-tetramethylethylenediamine
TMSPMA	3-(trimethoxysilyl)propyl methacrylate
TiO_2	titanium dioxide
VHB	very high bond tape
VO_2	vanadium dioxide
$ZnCl_2$	zinc chloride

English symbols

A_{sol}	solar absorption at the solar spectral range (0.28-2.5 μm)
D_c	annual average cooling degrees
D_h	annual average heating degrees
ΔD_c	annual average cooling degrees reduction
ΔD_h	annual average heating degrees reduction
dTr/dT	derivate of transmission-temperature
H_{ev}	enthalpy of water
h_{conv}	convective heat transfer coefficient
h_{cond}	conductive heat transfer coefficient
I_{BB}	blackbody radiation
I_{solar}	solar intensity
$I_{solar, avg}$	average solar intensity
$I_{solar, max}$	maximum value of solar intensity
K_y	mass transfer coefficient at the air side
K_y^*	effective mass transfer coefficient in the air side
$\dot{m}_{hydrogel}$	evaporation from the hydrogel surface
$\dot{m}_{water}$	evaporation rate of water
m_d	total mass of available water
P_{atm}	absorbed power by atmospheric thermal radiation to the sample surface
P_{cond}	transferred power by conduction to the sample surface
P_{conv}	transferred power by convection to the sample surface
P_{emit}	P_{rad}-P_{atm}, net emitted power from the sample surface considering P_{atm}
P_{evp}	evaporative power by desorption process from the sample surface

$P_{evp, water}$	evaporative power by desorption process from water surface
$P_{lantent}$	absorbed power from latent heat by adsorption process to the sample surface
$P_{non-rad}$	parasitic heating power due to convection and conduction
P_{water}	P_{evp}-P_{latent}, net evaporative power from the sample surface considering P_{latent}
P_{rad}	emitted power from the sample surface
P_{sun}	absorbed power by incident solar radiation to the sample surface
R_{sol}	solar reflection at the solar spectral range
ΔS	annual space-conditioning source energy savings
ΔS_c	annual space heating source energy savings
ΔS_h	annual space heating source energy savings
SCSES	space-conditioning source energy savings
$SCSES_{min}$	minimum value of SCSES found over existing roof materials
ΔS_{LRT}	source energy savings of LRT for space conditioning
ΔS_{min}	minimum value of source energy consumption of LRT for space conditioning over all existing conventional roofing material
T_{air}	ambient air temperature
$T_{air, avg}$	average ambient air temperature
$T_{cool\leftrightarrow heat}$	temperature threshold for human thermal comfort
T_{dew}	dew point temperature
T_{max}	maximum temperature of enclosure
T_{min}	minimum temperature of enclosure
T_s	surface temperature
$T_{s, max}$	maximum surface temperature over computed T_{air} interval
$T_{s, min}$	mimum surface temperature over computed T_{air} interval
T_{RC}	surface temperature of RC
$T_{RC, avg}$	average internal temperature of RC dome
T_{LRT}	surface temperature of LRT
$T_{plateau}$	plateau temperature
T_{sol}	transmission at the solar spectral range
T_{vis}	transmission at visible spectrum (0.38-0.78 μm)
ΔT_{avg}	average temperature difference
ΔT_{sol}	reduction in transmission at the solar spectal range

ΔT_{vis}	reduction in transmission for visible spectrum
v	wind speed
ΔW_d	cyclic weight loss of LRT
Y_a	humidity ratios near in the ambient air
Y_d	humidity ratios near the water surface

Greek symbols

α_c	linear-fitting coefficient of ΔS_c by ΔD_c
α_h	linear-fitting coefficient of ΔS_h by ΔD_h
ε_{IR}	spectral emission at infrared wavelength (2.5-16 μm)
γ	psychrometer constant

Supplementary Note 1. Determination of the evaporative cooling power on the hydrogel surface

1. LiBr-based evaporation suppression for enhanced water encapsulation

To evaluate the evaporative cooling performance of our hydrogel, we conducted a comparative analysis between two representative cases: a DI water-based hydrogel (Enhanced case) and an LiBr aqueous solution-based hydrogel (Reduced case), as shown in Figure S2a, Supporting Information. Notably, the Reduced case shows significantly suppressed evaporation throughout the simulation hours (9:30–16:30), both at low T_{air} (17.8–18.2 °C) and at higher T_{air} (18.2–39.6 °C) (Figure S2b, Supporting Information). This suppression was not observed in the Enhanced case, which is characterized by a smaller sample owing to active water loss. Derivative analysis of the evaporative power (P_{evp}) further highlights distinct rate behavior: in the Enhanced case, P_{evp} rises steeply near T_{air}≈ 10 °C, whereas in the Reduced case this rise is delayed and remains small across T_{air}≈ 10–40 °C (Figure S2c). Taken together, these results indicate that LiBr hydrogels effectively suppress surface evaporation across 10–40 °C, a range critical for maintaining temperature stability under moderate and cool conditions.

2. Determination of the evaporative cooling power on the hydrogel surface

To model the evaporative cooling power from a water surface ($P_{evp,\ water}$), we assume it is proportional to the evaporation rate ($\dot{m}_{water}$), which depends on the enthalpy of water (H_{ev}), the total mass of available water (m_d), and the cyclic weight loss (ΔW_d). Mathematically, this can be expressed as:

$$P_{evp,water}=H_{ev}\cdot m_d\cdot\Delta W_d=H_{ev}\cdot \dot{m}_{water} \quad \text{(S1)}$$

where H_{ev} is assumed to be 2400 J/g. The evaporation rate $\dot{m}_{water}$ is further defined as a function of the humidity difference between the surface and ambient air:

$$\dot{m}_{water} = K_y \cdot (Y_d - Y_a) \quad \text{(S2)}$$

Here, K_y is the mass transfer coefficient at the air side (kg/m²·s), which can be estimated as 25 + 19v, where v is wind speed in m/s. Y_d and Y_a denote the humidity ratios (g/kg) near the water surface and in the ambient air, respectively. Under no wind conditions (v = 0) and RH = 40%, the evaporation rate can be empirically described as:[1]

$$\dot{m}_{water} = -0.17264 + 0.10943 \cdot \exp\left(T_s/16.81535\right) \quad \text{(S3)}$$

where T_s is the surface temperature in degrees Celsius.

For hydrogels, which exhibit complex moisture transport due to their porous and hygroscopic nature, the evaporation process is further governed by internal diffusion and adsorption dynamics. Water exists in three primary forms: external vapour in the surrounding air (Y_a), free water in the pores (Y_d), and bound water adsorbed to the polymer matrix (W_d). When a moisture gradient exists between the hydrogel and its environment, diffusion processes—including intra- and inter-crystalline diffusion and interfacial mass transfer—enable water movement through the material (Figure S3a, Supporting Information). At equilibrium, Y_d and W_d are related through the desorption isotherm, and typically, $Y_d \approx Y_a$ when equilibrium is reached (Figure S3b, Supporting Information). The evaporation rate from the hydrogel surface ($m_{hy\dot{d}rogel}$) can therefore be expressed as:

$$m_{hy\dot{d}rogel} = m_d \cdot \frac{dW_d}{dt} = K_y{}^* \cdot (Y_d - Y_a) \tag{S4}$$

where W_d denotes the instantaneous water content of the hydrogel, and $K_y{}^*$ represents the effective mass transfer coefficient in the air side (kg/m^2·s). The humidity ratio Y_d within the hydrogel's pores can be deduced from the isotherm curves at assigned T_{air} and the RH. It is important to recognize that $K_y{}^*$ is influenced by the surface structure and convective conditions around the hydrogel. The $K_y{}^*$ can be established from experimental data. Specifically, the s-curved desorption behavior of Li-HPC-PAAm hydrogel within a narrow RH range of 60-80% contributes to a stable mass change rate and temperature plateau. During the plateau, the RH of air inside pores in the hydrogel is anchored around the inflection point of the isotherm, RH ~70% (Figure S3b, Supporting Information). Eventually, the calculation of the modified mass transfer coefficient $K_y{}^*$ settles on the determination of the mass change rate of the hydrogel:

$$K_y{}^* = \frac{m_{hy\dot{d}rogel}}{Y_d(T_{plateau}, 70\%) - Y_a(T_{air}, \mathrm{RH})} \tag{S5}$$

The moisture concentration inside the hydrogel can be ascertained by combining it with the plateau temperatures ($T_{plateau}$), as shown in Figure S3d, Supporting Information. In this setup shown is Figure S3c, Supporting Information, heater temperatures of 35 and 65 ºC represent our assumptions for moderate and hot ambient air conditions, respectively. Subsequently, the calculation of the modified mass transfer coefficient relies on determining the mass change rate of the hydrogel. As illustrated in Figure S3e, Supporting Information, the slopes of the two

dashed lines in the diagram are -6.30×10^{-4} and -1.06×10^{-3}, which correspond to the absolute desorption rates of 1.055×10^{-5} g/s and 1.767×10^{-5} g/s, respectively.

For the experiments at a heating power of 0.15 W, the temperature plateau is reached 29.7 °C (Figure S3d, Supporting Information). Then, the moisture concentration of air inside pores at 29.7 °C and RH of 70%, Y_d, can be determined to be 18.4 g/kg.[2] At the same time, the moisture concentration of ambient air at 25 °C and RH of 70% is 13.9 g/kg, according to the T_{air} and RH of the chamber. Then, the adjusted mass transfer coefficient under this circumstance can be determined as:

$$K_{y1}^{*}=\frac{\mathrm{d}m/\mathrm{d}t}{A\cdot(Y_d\text{-}Y_a)}=\frac{1.055\times10^{-5}\ \mathrm{g/s}}{0.0009\mathrm{m}^2\times(18.4\text{-}13.9)\mathrm{g/kg}}=0.0026\ \mathrm{kg/m^2\cdot s} \quad \text{(S6)}$$

Similarly, for the condition of 0.57 W of heating power, Y_d can be determined to be 116.3 g/kg ($T_{plateau}$ at 62.9 °C). Then,

$$K_{y2}^{*}=\frac{\mathrm{d}m/\mathrm{d}t}{A\cdot(Y_d\text{-}Y_a)}=\frac{1.767\times10^{-5}\ \mathrm{g/s}}{0.0009\mathrm{m}^2\times(116.3\text{-}13.9)\mathrm{g/kg}}=0.0019\ \mathrm{kg/m^2\cdot s} \quad \text{(S7)}$$

The mass transfer coefficients calculated from two series of experimental data are very close, indicating that the mass transfer is insensitive to temperature in the concerned range. Hence, the average value, 1.40×10^{-3} kg/m²·s, will be used as K_y^* in further simulations. Hence, the evaporative cooling power that arises on our hydrogel surface, denoted as $P_{evp,hydrogel}$, can be derived from values fitted to the temperature (T) of the hydrogel, ranging from 0 °C≤ T ≤ 50 °C (Figure S3f, Supporting Information):

$$P_{evp,hydrogel}\ (\mathrm{RH}=70\%)=H_{ev}\cdot[\dot{m}_{hydrogel}]=H_{ev}\cdot[K_y^*\cdot(Y_d\text{-}Y_a)]$$
$$=H_{ev}\cdot[-12.1791+2.7441\cdot\exp(T/17.2651)] \quad \text{(S8)}$$

To obtain the $P_{evp,hydrogel}$ value for outside the T range of 0 °C≤ T≤ 50 °C, the $P_{evp,hydrogel}$ is re-evaluated (blue dashed curve, marked as Evaluated $P_{evp,hydrogel}$ (RH=70%)) using the equation (S8) (Figure S3f, Supporting Information). Although it is stated that the evaporation behavior of Li-HPC-PAAm hydrogel occurs within a narrow RH range of 60-80% according to isotherm curve in Figure S3b, Supporting Information, the figure actually shows water desorption occurring within a wider RH range of 30% to 80%. Therefore, considering the dependency on RH,[3] the calculation of $P_{evp,hydrogel}$ with consideration for this RH dependence, denoted as $P_{evp,hydrogel}$ can be expressed:

$$P_{evp,hydrogel} = H_{ev} \cdot [-12.1791 + 2.7441 \cdot \exp(T/17.2651)] \cdot 0.7 \cdot (1/RH) \qquad (S9)$$

The calculated results of the evaporative cooling power and temperature occurring at the water surface ($P_{evp,water}$) and our Li-HPC-PAAm hydrogel surface ($P_{evp,hydrogel}$), where RH is 40%, using equation (S9) (Figure S3g,h, Supporting Information).

Supplementary Note 2. Energy balance model for calculating temperature of LRT

Here we suggest a simple solution for the energy balance model of latent and radiative thermostat (LRT) (Figure S5a–c). Simulations were performed using Mauna Kea sky transmittance and a convective heat transfer coefficient (h_{conv}) of 2 W/m²·K, under 1000 W/m² of solar irradiance. If an object is exposed to the sky, it exchanges heat with surroundings. The temperature of an object is determined by energy balance:

$$P_{net} = P_{rad} - P_{atm} - P_{sun} + P_{evp} - P_{latent} - (P_{conv} + P_{cond}) \quad \text{(S10)}$$

In equation (S10), P_{sun} represents absorbed solar power on the object given by:

$$P_{sun} = \int_0^{\infty} I_{AM1.5G}(\lambda)\epsilon(\lambda,\theta)\mathrm{d}\lambda \quad \text{(S11)}$$

where λ is wavelength, $I_{AM1.5G}(\lambda)$ represents a global horizontal irradiance spectrum, and $\epsilon(\lambda,\theta)$ indicates spectral and angular emissivity of an object.

P_{rad} is the power radiated out from the object, which is expressed by:

$$P_{rad} = \int_0^{2\pi}\int_0^{\pi/2}\int_0^{\infty}\{I_{BB}(T,\lambda)\epsilon(\lambda,\theta)\cos(\theta)\sin(\theta)\}\,\mathrm{d}\lambda\mathrm{d}\theta\mathrm{d}\phi \quad \text{(S12)}$$

where $I_{BB}(T,\lambda) = (2hc^2/\lambda^5)/[e^{hc/\lambda k_B T} - 1]$ is the spectral radiance of a blackbody at an object's temperature (T), with h as the Plack's constant, c as the velocity of light, and k_B as the Boltzmann constant.

P_{atm} denotes the power absorbed from atmosphere by the object and expressed by:

$$P_{atm} = \int_0^{2\pi}\int_0^{\pi/2}\int_0^{\infty}\{I_{BB}(T_{air},\lambda)\epsilon(\lambda,\theta)\cos(\theta)\sin(\theta)\cdot\{\epsilon_{atm}(\lambda,\theta)\}\,\mathrm{d}\lambda\mathrm{d}\theta\mathrm{d}\phi \quad \text{(S13)}$$

P_{evp} represents the evaporative cooling power of the hydrogel, which is described as:[4]

$$P_{evp} = H_{ev}\cdot m_d\cdot\Delta W_d \quad \text{(S14)}$$

in which H_{ev} is the enthalpy of water and m_d indicates the mass of the total water in the hydrogel (in grams), and ΔW_d ($g_{water}/g_{hydrogel}$) is the consequent cyclic water uptake calculated as the final water content ($g_{water}/g_{hydrogel}$) minus the initial water content ($g_{water}/g_{hydrogel}$) of the hydrogel. P_{latent} denotes absorbed power from latent heat by adsorption process to the sample surface, which is given by:[5]

$$P_{\text{latent}} = (\text{Le})^{\text{n}} \cdot \frac{h_{\text{conv}}}{\gamma}\{\text{RH} \cdot P_{\text{H}_2\text{O}}(T_{\text{air}}) - P_{\text{H}_2\text{O}}(T)\} \quad \text{(S15)}$$

where Le is the Lewis number (equal to 0.87 at 300 K) and n is set to be -4/3 considering natural convection at horizontal plate. Here, h_{conv} and γ represent the convection coefficient and the psychrometer constant (0.067 kPa K^{-1} at 20 °C), respectively. In the equation (S15), the saturation vapor pressure, $P_{\text{H}_2\text{O}}$, with unit of [10^{-3} bar] can be expressed as:

$$P_{\text{H}_2\text{O}} = T^{\text{a}} 10^{\text{b}/T + \text{c}} \quad \text{(S16)}$$

where the parameters are defined as follows: a = -4.9283, b = -2937.4 K, c = 23.5518. These values are valid within the temperature range of -50 °C to 100 °C.

P_{conv}+P_{cond} in equation (S10) is the transferred power by convection and conduction, which is given by:

$$P_{\text{conv}} + P_{\text{cond}} = P_{\text{non-rad}} = (h_{\text{conv}} + h_{\text{cond}}) \cdot (T_{\text{air}} - T) \quad \text{(S17)}$$

where h_{conv} is convective heat transfer coefficient and h_{cond} is conductive heat transfer coefficient, with unit of [$\text{W/m}^2\,\text{K}^{-1}$].

As shown in Figure S5a, the equation (S10) can be divided into four parts: radiative cooling power term (P_{emit}), evaporative cooling power term (P_{water}), parasitic heating power term due to convection and conduction ($P_{\text{non-rad}}$), and solar heating term (P_{sun}).

P_{emit} denotes net cooling power from the surface of LRT by emission of infrared wavelength range, which can be expressed as:

$$P_{\text{emit}} = P_{\text{rad}} - P_{\text{atm}} \quad \text{(S18)}$$

P_{water} denotes net cooling power from the surface of LRT by water adsorption and desorption behavior, which can be expressed as:

$$P_{\text{water}} = P_{\text{evp}} - P_{\text{latent}} \quad \text{(S19)}$$

We supposed that there is no conduction heat transfer (namely, h_{cond} =0, P_{cond} = 0), thus, the lost power term due to convection and conduction is $P_{\text{non-rad}} = P_{\text{cond}}$.

Therefore, equation (S10) is simplified, and the theoretical net heat transfer power of LRT is obtained by:

$$P_{\text{net}} = P_{\text{emit}} + P_{\text{water}} - P_{\text{sun}} - P_{\text{conv}} \quad \text{(S20)}$$

Supplementary Note 3. Energy-Balance Analysis of Latent–Radiative Coupling: Climate/Diurnal Comparisons and Design Implications (R_{sol}, A_{sol})

We analyze the coupled latent–radiative heat that governs the LRT's surface temperature (T_s) under varying T_{air}, RH, and sky transmittance. Using $P_{emit} = P_{rad} - P_{atm}$, $P_{water} = P_{evp} - P_{latent}$, and P_{sun} with $h_{conv} = 2$ W/m²·K, we compare ideal (Mauna Kea) and non-ideal (Sub-Arctic, Tropical) skies over day/night. The output includes contribution ratios, T_s flatness over $T_{air} = 0 - 60$ °C, and practical R_{sol}/A_{sol} guidance (e.g., higher R_{sol} in humid skies), clarifying when thermochromic R_{sol} is advantageous.

1. Latent–radiative coupling (mechanistic summary)

- Self-regulated mass transfer (water channel): Daytime evaporation increases P_{evp} (stronger at high T_{air}/lower RH); nighttime regeneration releases P_{latent}. Define $P_{water} = P_{evp} - P_{latent}$ (positive = net cooling by evaporation; negative = net heating by regeneration).
- Spectral power balance: High IR emissivity ($\varepsilon_{IR} \approx 1$) maximizes P_{emit}, while R_{sol} modulates P_{sun} (less solar gain when hot, more when cool).

2. Negative feedback (stability)

   Evaporation cools the surface ($T_s\downarrow$), reducing the vapor-pressure driving force and thus reduce $P_{evp}\downarrow$(cooling weakens). Radiative loss scales as $P_{rad} \approx T_s^4$, so $T_s\downarrow \rightarrow P_{rad}\downarrow$ (cooling further weakens). With both cooling channels reduced, the remaining heating terms dominate (e.g., P_{sun}, P_{conv} toward T_{air}, and adsorption when $T_s < T_{dew}$), turning the net heat balance positive and driving $T_s\uparrow$. Conversely, $T_s\uparrow$ increases P_{evp} and P_{rad} (cooling strengthens), turning the net balance negative and driving $T_s\downarrow$. Hence, any deviation of T_s from equilibrium triggers opposing changes in P_{evp} and P_{rad} that restore the balance, confining T_s to a stable band.

3. Contribution ratios across climates and humidity

- Dry daytime (Mauna Kea sky; Figure S4 (g)-(i))
  - Water cooling fraction at $T_{air} =$ 0/ 30/ 60 °C: 0.14/ 0.37/ 0.83; Radiative cooling fraction: 0.86/ 0.63/ 0.17.
    → P_{emit} dominates at low-mid T_{air}, P_{water} dominates at high T_{air}.
- Humid–daytime (Tropical sky; Figure S4 (d)-(f))
  - Water cooling fraction at $T_{air} =$ 0/ 34/ 60 °C: 0.00/ 0.69/ 0.78; Radiative

cooling fraction: 1.00/ 0.31/ 0.22.

→ P_{water} share rises earlier (mid-T_{air}).

- Nighttime (Figure S4(j)):
  - Often $T_s < T_{dew}$ (*e.g.*, $T_{air} = 0 \rightarrow 10$ °C: $T_{RC} = -21.6 \rightarrow -13.6$ °C; $T_{LRT} = -19.8 \rightarrow -10.2$ °C; $T_{dew} = -4.82 \rightarrow 4.78$ °C).

    → Water regeneration yields $P_{water} < 0$ (or $P_{latent} > 0$), the dominant night-time heating; LRT stays warmer than RC.

4. Daytime flatness (temperature band) and design implications (R_{sol}, A_{sol})

- Temperature band over T_{air} = 0–60 °C
  - Mauna Kea: T_{LRT} 10.8 °C (range 22.8–33.6 °C), T_{RC} (range 14.0–51.8 °C) → RC/LRT flatness ratio ≈ 3.5×
  - Sub-Arctic: T_{LRT} 13.2 °C (range 22.0–35.2 °C), T_{RC} 44.6 °C (range 8.8–53.4 °C) → RC/LRT ≈ 3.4×
  - Tropical: T_{LRT} 35.0 °C (range 6.8–41.8 °C), T_{RC} 54.6 °C (range 0.4–55.0 °C) → RC/LRT ≈ 1.6×
  - LRT narrows the surface-temperature band most under dry/clear conditions (Mauna Kea), remains strongly effective in Sub-Arctic, and still improves stability in Tropical regions, although to a lesser extent. Dry/clear skies (small P_{atm}) strengthen P_{emit}, allowing modest P_{sun} (e.g., $R_{sol} \approx 65\%$ flattens $T_s - T_{air}$. In humid skies (large P_{atm}), P_{emit} weakens and latent share grows; P_{sun} must be suppressed (e.g., $R_{sol} \approx 89\%$) to avoid overheating.
- Power-balance summary (negative = heating)
  - Mauna Kea: $P_{sun} = -348$ W/m² ($A_{sol} = 35\%$, $R_{sol} = 65\%$), $P_{emit} = 253/\ 219/\ 72$, $P_{water} = 42/\ 127/\ 342$ ($T_{air} = 0/34/60$ °C).
  - Tropical: $P_{sun} = -109$ W/m² ($R_{sol} \approx 89\%$), $P_{emit} = 87/\ 35/\ -55$, $P_{water} = 0/\ 77/\ 200$.

    → Dry/clear allows larger $|P_{sun}|$ (heating) while high T_{air} shifts dominance to P_{water}; humid conditions require minimizing P_{sun} to avoid overheating.

5. Ratio computation

- At each equilibrium state, we solve the steady energy balance: $P_{water} + P_{emit} + P_{sun} + P_{conv} = 0$. Here, for visualization, each power component is plotted with its algebraic sign (e.g., $P_{sun} < 0$ = heating, $P_{emit} > 0$ = radiative cooling, $P_{water} < 0$ = latent heating).
- Contribution ratios
  - Contribution ratio (latent)= $|P_{water}|\ /\ (|P_{water}|\ +\ |P_{emit}|)$,

- Contribution ratio (radiative) = $|P_{emit}| / (|P_{water}| + |P_{emit}|)$.
- Flatness ratio (temperature stability): We define band $T_s \equiv T_{s,\,max} - T_{s,\,min}$ over $T_{air} =$ 0–60 °C. Then

  $\text{Flatness ratio} = \text{band } T_{RC} / \text{band } T_{LRT}$.

6. Practical implication

- Because LRT rebalances P_{water}/P_{emit} with T_{air}, RH, and sky, it maintains a flatter, comfort-oriented T_s by day and avoids over-cooling at night. Design tuning with R_{sol}/A_{sol} is detailed in Note S4, Supporting Information.

Supplementary Note 4. Optimization of solar reflectance for RC and LRT

This optimization builds on the energy-balance decomposition and climate/diurnal comparisons in Note S3, Supporting Information. Materials with appropriate solar absorption (A_{sol}) can passively switch between cooling and heating states depending on ambient air temperature (T_{air}), without requiring dynamic control of emissivity or external energy input. In the energy balance equations (S20), this behavior is governed by the relationship between the object's temperature (T) and the T_{air}. When $T < T_{air}$, the object is in a cooling state, and when $T > T_{air}$, it is in a heating state. This transition point can be understood by comparing the blackbody radiation intensity (I_{BB}) at each temperature; higher I_{BB} values correspond to higher equilibrium temperatures.

As shown in Figure S5e, a radiative cooler (RC) with high ε_{IR} of 100% for wavelength over 4 μm and low A_{sol} from 0 to 35% exhibits both heating and cooling states depending on T_{air}. For instance, an RC with A_{sol} = 22% (i.e., R_{sol} = 78%) achieves temperature of approximately heating temperature of 14.4 °C under T_{air} = 0 °C and cooling temperature of 7.8 °C under T_{air} = 60 °C. From Figure S5e and equation (S12), assuming a constant solar reflectance (R_{sol}) of 78% (or solar absorptance (A_{sol}) of 22%) results in P_{sun} of 220 W/m^2, the relationship between T and T_{air} is reversed when T_{air} equals 34 °C, according to the energy balance model described in equation (S10).

In comparison, LRT exhibits more complex behavior due to their combined evaporative and radiative cooling, solar and latent heating, and thermochromism. Figure S5d shows that the transition point between heating and cooling in LRT occurs at a similar $T_{air} \approx$ 34 °C, but requires a lower R_{sol} of approximately 65% to maintain thermal balance. This reduced reflectance accounts for the additional cooling contribution from water evaporation, which supplements the radiative loss and allows the LRT to operate at slightly lower solar reflectance while achieving comparable cooling performance.

All simulations were performed assuming an ideal sky transmission window based on the Mauna Kea transmittance, with a non-radiative heat exchange coefficient of h_c of 2 W/m^2·K.

Supplementary Note 5. Energy saving performance simulation

1. Estimation of energy savings

To assess the sensitivity of A_{sol} and ε_{IR} on building heating and cooling energy consumption, a geometrical model of an independent retail building prototype has been proposed (Figure S8, Supporting Information). Specifically, the building prototype has a dimension of 46.1 m (L) x 34.7 m (W) x 5.1 m (H). The heating and cooling energy demands are simulated using whole-building energy modeling with EnergyPlus. The representative seven global climate zones were selected based on ASHRAE climatic classifications for the simulations:[6] Honolulu, USA (Zone 1), Cairo, Egypt (Zone 2), Jeju, Republic of Korea (Zone 3), Albuquerque, USA (Zone 4), Manchester, England (Zone 5), Stockholm, Sweden (Zone 6), Whitehorse, Canada (Zone 7) (Figure 1g; Table S2, Supporting Information). Moreover, additional global cities across different zones are included for estimating world heating and cooling energy savings of LRT compared to existing roofing materials: Bangkok, Thailand; Singapore, Singapore; Manaus, Brazil; Abu Dhabi, U.A.E; Karachi, Pakistan; Port Hedland, Australia; Tegucigalpa, Honduras; Nairobi, Kenya; Tamanrasset, Algeria; Atlanta, USA; Auckland, New Zealand; Adelaide, Australia; Cape Town, South Africa; Gwangju, Republic of Korea; Lyon, France; Seoul, Republic of Korea; Beijing, China; Salt Lake City, USA; Denver, USA; Ottawa, Canada; Helsinki, Finland; Ushuaia, Argentina; Tampere, Finland; Calgary, Canada; International Falls, USA; Ekaterinburg, Russia; Qaqortoq, Greenland; Yakutsk, Russia; Resolute, Canada (Figure 1h; Table S3, Supporting Information).

As shown in Figure 1f, for the hour i of the year, heating degrees $D_{h,i}$ and cooling degrees $D_{c,i}$ are expressed by:

$$D_{h,i} = (23\ °C - T_{s,i})_i \tag{S21}$$

$$D_{c,i} = (T_{s,i} - 23\ °C)_i \tag{S22}$$

where $x_i = x$, if $x > 0$ otherwise it is 0, $T_{s,i}$ represents temperature of sample for the hour i of the year. The annual averaged heating degree (D_h) and cooling degrees (D_c) can be obtained by averaging $D_{h,i}$ and $D_{c,i}$, respectively. Here we set 23 °C as a heating and cooling set points and estimated that the building would need heating when the object's surface temperature (T_s) is 23 °C or lower, and cooling when it exceeds 23 °C.[7]

The annual space heating source energy savings (ΔS_h) and the annual space cooling source energy savings (ΔS_c) are given by:

$$\Delta S_{\mathrm{h}} = \alpha_{\mathrm{h}} \Delta D_{\mathrm{h}} \tag{S23}$$

$$\Delta S_{\mathrm{c}} = \alpha_{\mathrm{c}} \Delta D_{\mathrm{c}} \tag{S24}$$

where ΔD_{h} and ΔD_{c} denotes annual average heating degrees reduction and annual average cooling degrees reduction, respectively. As shown in equations (S23, S24), ΔS_{h} and ΔS_{c} are derived from ΔS_{h} and ΔS_{c} with the linear-fitting parameters of α_{h} and α_{c}. To extract the linear-fitting coefficients of α_{h} and α_{c}, energy simulations are conducted for static roofing materials with R_{sol} = 10%, 25%, 40%, and 60% (corresponding to A_{sol} = 90%, 75%, 60%, 40%) and $\varepsilon_{\mathrm{IR}}$ = 90%. The material with A_{sol} = 90% is used as the reference point (Figure S5, Supporting Information). By simply adding ΔS_{h} and ΔS_{c}, the total annual space-conditioning source energy savings ΔS is obtained by:

$$\Delta S = \Delta S_{\mathrm{h}} + \Delta S_{\mathrm{c}} = \alpha_{\mathrm{h}} \Delta D_{\mathrm{h}} + \alpha_{\mathrm{c}} \Delta D_{\mathrm{c}} \tag{S25}$$

Based on equations (S21–S25), the space-conditioning source energy savings (SCSES) per unit area of LRT are obtained by calculating the difference in values between existing roofing materials, which show static A_{sol} varying from 0% to 100%. The heating and cooling degree reductions of LRT A_{sol} = 56%, ΔA_{sol} = 14%, and $\varepsilon_{\mathrm{IR}}$ = 100%), assumed to be placed on a colored roof (A_{sol} = 56%, ΔA_{sol} = 0% for simplicity, and IR emissivity ($\varepsilon_{\mathrm{IR}}$) = 100%), in comparison to RC (A_{sol} = 56%, $\varepsilon_{\mathrm{IR}}$ = 100%) and other existing roof coating materials, such as RC (A_{sol} = 56%, $\varepsilon_{\mathrm{IR}}$ = 100%), white non-metal roof (A_{sol} = 5%, $\varepsilon_{\mathrm{IR}}$ = 95%), black non-metal roof (A_{sol} = 95%, $\varepsilon_{\mathrm{IR}}$ = 95%), and metal roof (A_{sol} = 35%, $\varepsilon_{\mathrm{IR}}$ = 25%), are given by:

$$\Delta D_{\mathrm{h,LRT}}(A_{\mathrm{sol}}, \varepsilon_{\mathrm{IR}}) = D_{\mathrm{h,LRT}} - D_{\mathrm{h}}(A_{\mathrm{sol}}, \varepsilon_{\mathrm{IR}}) \tag{S26}$$

$$\Delta D_{\mathrm{c,LRT}}(A_{\mathrm{sol}}, \varepsilon_{\mathrm{IR}}) = D_{\mathrm{c,E-RC}} - D_{\mathrm{c}}(A_{\mathrm{sol}}, \varepsilon_{\mathrm{IR}}) \tag{S27}$$

Using the equations (S23–S27), the SCSES of LRT (ΔS_{LRT}) can be determined as:

$$\Delta S_{\mathrm{LRT}}(A_{\mathrm{sol}}, \varepsilon_{\mathrm{IR}}) = \alpha_{\mathrm{h}} \Delta D_{\mathrm{h,LRT}}(A_{\mathrm{sol}}, \varepsilon_{\mathrm{IR}}) + \alpha_{\mathrm{c}} \Delta D_{\mathrm{c,LRT}}(A_{\mathrm{sol}}, \varepsilon_{\mathrm{IR}}) \tag{S28}$$

The minimum value of $\Delta S_{\mathrm{LRT}}(A_{\mathrm{sol}}, \varepsilon_{\mathrm{IR}})$ (namely, $\mathrm{SCSES_{min}}$) is derived from calculating the difference between SCSES of LRT and existing roofing materials. According to equations (S21–S28), $\mathrm{SCSES_{min}}$ is calculated for all types of existing roofing materials and compared using energy saving mapping, as shown in Figures S6–S9.

2. Energy saving of LRT compared to existing roofing materials

Refer to Figures S25 and S26 for the flowchart detailing the process of calculating the total $SCSES_{min}$. The SCSES is determined by summing ΔS_h and ΔS_c, which are derived from ΔD_h and ΔD_c (Figures S6I-IV and S9I-IV, Supporting Information). The energy saving map denotes the minimum space conditioning source energy saving of LRT ($SCSES_{min}$) compared to all other existing roofing materials. In other words, for comparison between energy saving of LRT and existing roofing materials, the most energy-saving material (static A_{sol} and ε_{IR}) is initially chosen from all existing roofing materials. Therefore, $SCSES_{min}$ indicates the extra source energy saving of LRT compared to the best performance for energy saving of existing roofing material, resulting in the figure of merit map shown in Figures S6V, S9V.

Supplementary Note 6. Optimization of HPC and Li+ concentration in LRT depending on target applications

Here we deal with adjusting Li^+ and HPC concentration in LRT, focusing on innovation to the evaluation of the hydrogel for thermostat performance in real-life scenarios. For effective thermostat performance, it is essential to analyze the thermostat performance influenced by dynamic optical characteristics, water evaporation and regeneration behaviors. Based on theoretical calculation of optical characteristics, the optimization of HPC concentrations within LRT, which shows thermochromic characteristics, is conducted for effective thermal regulation. Figures 3a and S10f,g in the Supporting Information show T_{vis} and T_{sol} variation of the LRT at 25 and 55 ºC, with various HPC weight/AAm weight ratios of 0.08, 0.17, 0.25, 0.33, 0.38, and 0.51. As the HPC concentration of LRT increases, the variations of T_{vis} and T_{sol} increase. The HPC weight/AAm weight ratio of LRT is optimized to 0.33 owing to its similar optical properties of *Populus alba* populated in Gwangju, as can be seen in Figure S1 in the Supporting Information.

Figure S14a presents the thermal regulation measurements under varying climate conditions, including I_{solar}, RH and T_{air}. Initially, based on the average solar intensity (I_{solar} ~ 0.5 kW/m^2), the measured temperature is categorized into two cooling regions: the RC cooler region (RC CR) and LRT cooler region (LRT CR). At night, RC exhibits an average temperature reduction of ~2°C, achieving lower temperatures (indicating higher cooling efficiency) than both $LRT_{15\%}$ and $LRT_{35\%}$ (Figure S14b, Supporting Information). This effect is attributed to the latent heat release of LRTs due to moisture adsorption, as indicated by positive derivative values of weight variation (dm/dt). Moreover, $LRT_{35\%}$ shows a slightly higher average temperature (indicating less cooling, more towards heating) of ~0.8°C compared to $LRT_{15\%}$, a discrepancy resulting from the differing hygroscopic properties of the hydrogels at these concentrations. During the day, however, LRTs exhibit enhanced cooling performance relative to conventional RC (Figure S13b, Supporting Information). Figure S14c,d further detail the observations from Figure S14a, focusing on concentrations of $LRT_{15\%}$ and $LRT_{35\%}$ within the CR. Under subhumid conditions (RH > 78%), $LRT_{15\%}$ exhibits better cooling performance compared to $LRT_{35\%}$. Conversely, in arid environments (RH < 78%), $LRT_{35\%}$ exhibits better cooling effectiveness than $LRT_{15\%}$. These results corroborate the discussions in Figure 3c–g, showcasing how the concentration of Li^+ ion within LRT influences its water evaporation regulation capability, thereby adjusting its thermal regulation properties to accommodate various humidity levels.

Supplementary Note 7. Comparison of thermal regulation performance among RC, LRT without HPC, and LRT

The outdoor temperature and weight variation are measured to evaluate the thermal regulation performance of LRT. The temperatures of RC and LRT without HPC are simultaneously measured for an intuitive assessment of the thermal regulation performance of LRT as well as the effect of the solar and latent heating, combining radiative and evaporative cooling, and thermochromism. Continuous field tests under various atmospheric conditions and on different substrates are conducted to ensure the reliability of the proposed structure.

Surface temperatures (T_s) of each sample and weight variation of LRT are measured on hot days ($T_{air, avg}$ > 20 ºC) (Figure S20a–e, Supporting Information) and on a cold day ($T_{air, avg}$ < 20 ºC) (Figure S20f, Supporting Information). On hot days—the situation requiring cooling—both LRT and LRT without HPC maintains lower average T_s compared to RC during daytime (I_{solar} > $0.5 \cdot I_{solar, max}$), due to their combined radiative and evaporative cooling effect. Moreover, hydrophobic interaction between HPC molecules and water molecules facilitates water evaporation, replenishing the lower radiative cooling power resulting from the reduced R_{sol} compared to the theoretical calculation.[8] Additionally, the average T_s of LRT exhibits a lower temperature than the T_s of LRT without HPC, attributed to a gradual change in transmittance as shown in Figure 2c.[9]

Conversely, on a cold day, the average T_s of LRT is higher than that of RC, originating from the solar and latent heating effect of LRT. The LRT exhibits a higher T_s than RC during daytime under cold conditions, primarily due to solar heating effect associated with its higher A_{sol}. Notably, during the nighttime—defined as the time range without temperature fluctuation—the LRT also maintains a higher T_s than RC, attributed to latent heat release from moisture adsorption. Interestingly, the T_s of LRT without HPC exhibits similar average T_s to LRT, because both are fabricated with the same amount of LiBr weight. Figure 4b summarizes the average T_s results for RC and LRT. The experimental results are well-matched with the estimated average T_s for the mid-latitude sky condition, highlighting the superior performance of the proposed structure in managing thermal conditions in environments with varying ambient temperatures.

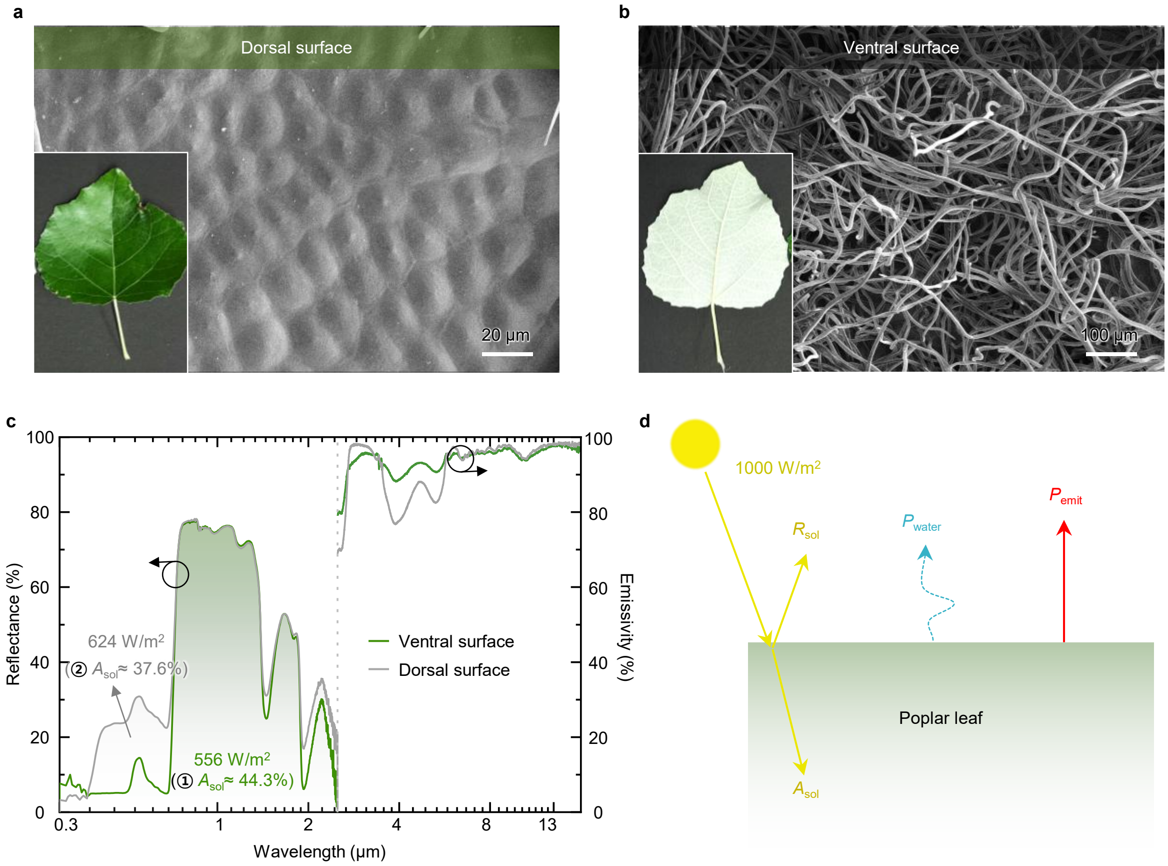

Figure S1. Optical and thermoregulatory characteristics of *Populus alba* leaves. a,b) SEM and photograph images (insets) of *Populus alba* of the (a) glabrous dorsal surface and the (b) hairy ventral surface of the poplar leaves. c) Reflectance and emissivity spectra of the two surfaces of the leaf. The gray and green solid lines indicate optical properties of the dorsal surface and ventral surface of a poplar leaf, respectively. d) Energy balance model of a poplar leaf. To calculate the solar reflectance (R_{sol}) and solar absorption (A_{sol}) of the dorsal and ventral surfaces of a leaf, the solar intensity (I_{solar}) is set at 1000 W/m². P_{water} represents the net evaporative power, while P_{emit} denotes the emitted power by the poplar leaf.

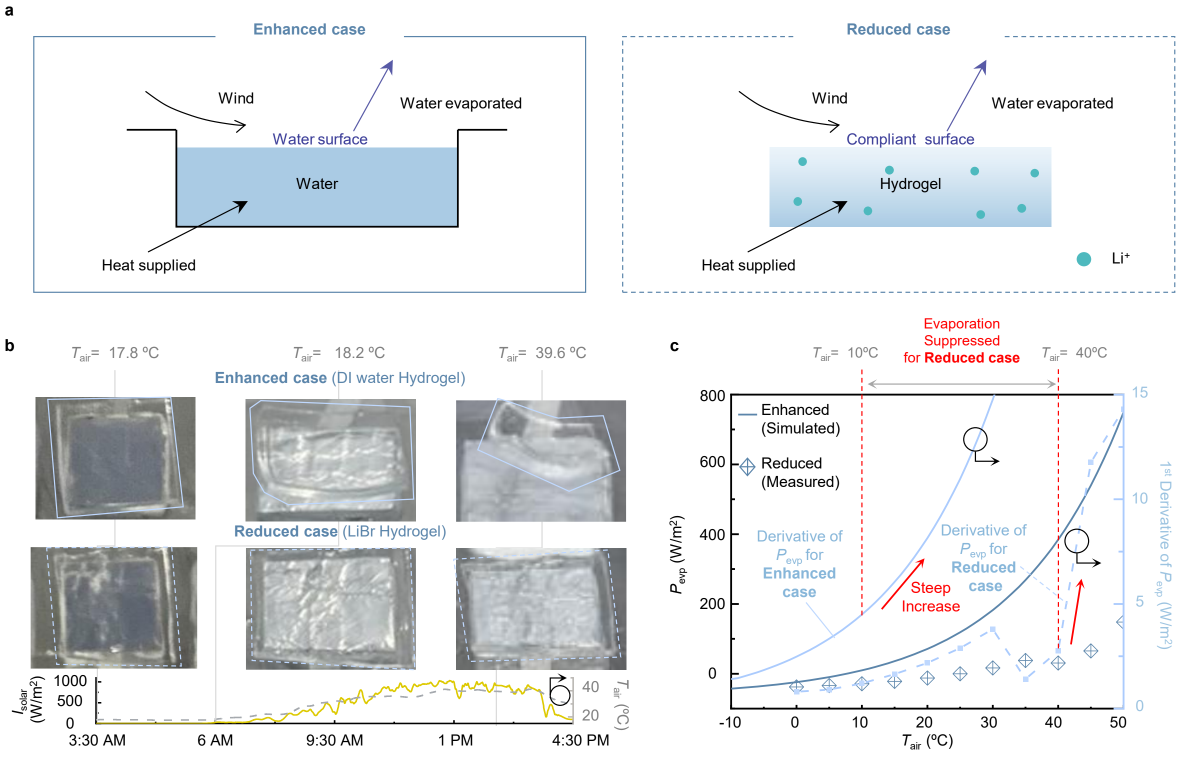

Figure S2. Experimental evaluation of evaporative cooling power (P_{evp}) in enhanced and reduced cases. a) Schematic diagrams illustrating evaporation behavior from (left) a free water surface and (right) a hydrogel surface containing Li^+ ions. In the LiBr-loaded sample, ionic binding suppresses evaporation by lowering vapor pressure. b) Time-dependent weight loss measurements under outdoor conditions (Gwangju, Republic of Korea; 35.17°N, 126.88°E; 9 June, 2023) comparing a DI water-based hydrogel (Enhanced case) and a LiBr aqueous solution-based hydrogel (Reduced case). Evaporation in the reduced case is significantly suppressed between 9:30 AM and 1:00 PM, when ambient temperature (T_{air}) is relatively low (17.8–18.2 °C) and when it is high (18.2–39.6 ºC). c) Estimated evaporative cooling power (P_{evp}) for both cases. The derivative of P_{evp}, which occurs at ~10 °C for the Enhanced case and is delayed to ≥ 40 °C for the Reduced case (sky blue line and dashed sky blue line, respectively).

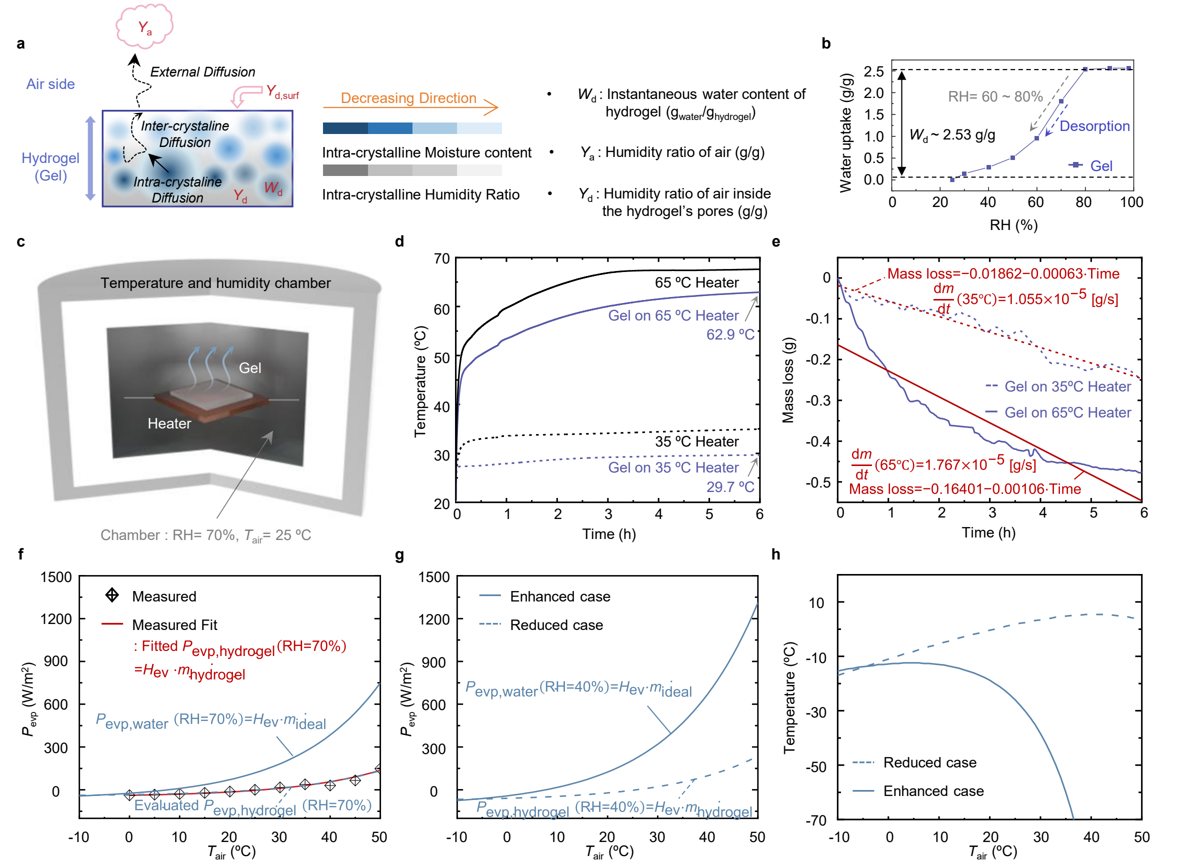

Figure S3. Experimental characterization and modeling of evaporative cooling power (P_{evp}) for LRT. a) Conceptual mass transfer model describing water desorption from hydrogel pores to ambient air, driven by the humidity gradient across the hydrogel–air interface. b) Water desorption isotherm of LRT showing S-shaped behavior, with a transition region around RH ≈ 70%. c) Schematic of the temperature–humidity-controlled test chamber used for evaporation experiments. A thin-film resistance heater beneath an aluminum (Al) foil layer minimizes thermal loss. d,e) Experimental results of (d) surface temperature increase and (e) corresponding water mass loss rate under heating powers of 0.15 W ($T \approx 35$ °C) and 0.57 W ($T \approx 65$ °C). f) Fitted expression for P_{evp} of the hydrogel based on experimental data, assuming latent heat of evaporation H_{ev} = 2400 J/g. The effective evaporation rate $\dot{m}_{hydrogel}$ is modeled as $\dot{m}_{hydrogel}$= -12.1791+2.7441·exp(T/17.2651), and extended to estimate evaluated P_{evp}, hydrogel over a broader temperature range. g,h) Comparison of modeled (g) evaporative cooling power and resulting surface temperature (h) for pure water (Enhanced case) and LRT (Reduced case) at RH = 40%. The Reduced case shows flatter temperature curves over T_{air}, suggesting more stable thermostat behavior.

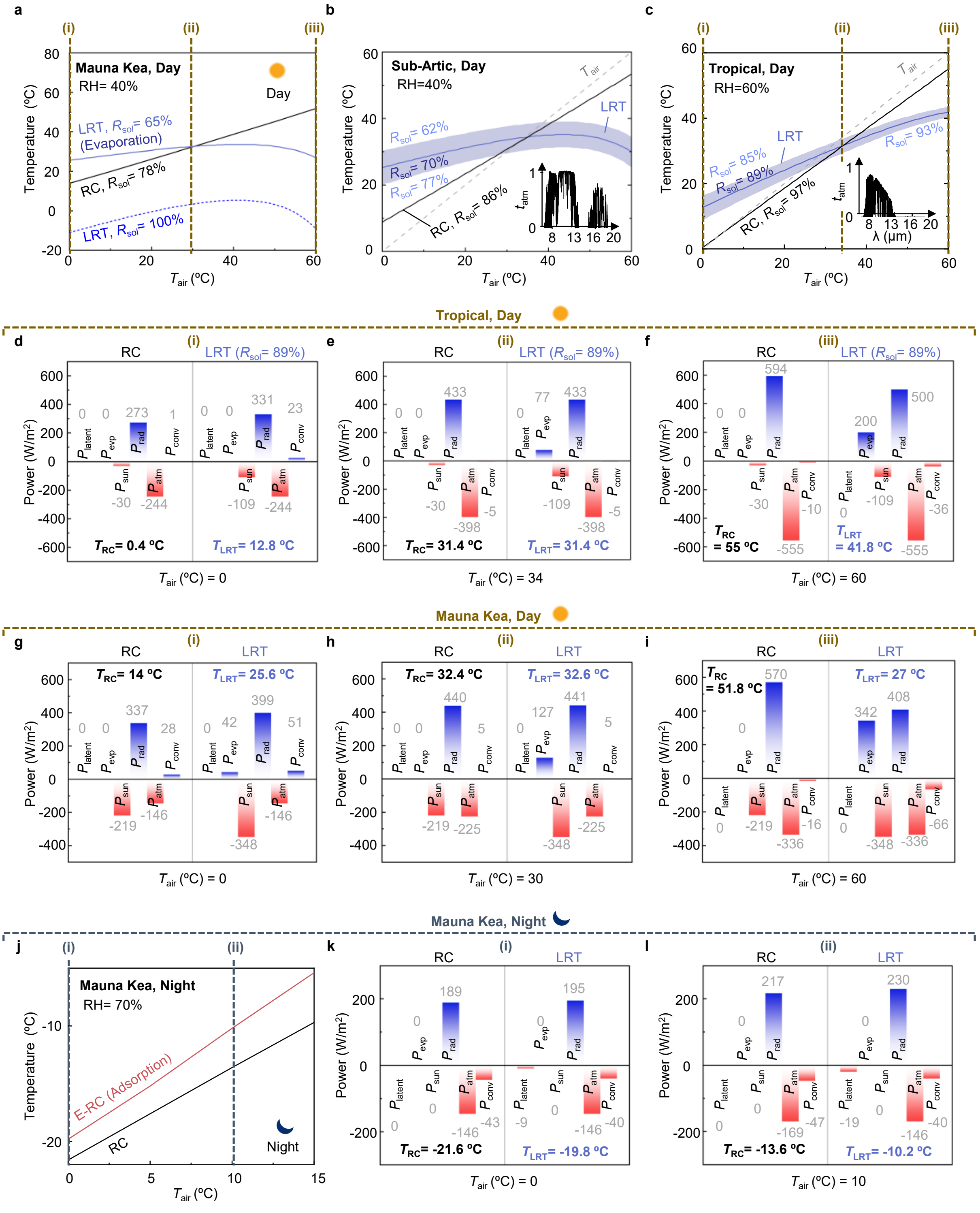

Figure S4. Simulated surface temperatures and power of RC and LRT across climatic and diurnal conditions. All simulations assume a convective heat transfer coefficient (h_{conv}) of 2 W/m²·K. a) Daytime simulations of RC (black line; R_{sol} = 78%, ε_{IR} = 100%) and LRT (blue line; R_{sol} = 65%, ε_{IR} = 100%) under ideal sky transmission condition (Mauna Kea sky) as a function of ambient air temperature (T_{air}). The LRT shows a flatter temperature profile due to evaporative cooling and dynamic solar modulation, enabling it to maintain temperatures closer to the thermal comfort range. In (a), the blue line shows LRT temperature for R_{sol} = 65% (A_{sol}= 35%), yielding a flatter temperature–T_{air} curve and maintaining ~25.6–33.6 °C for T_{air} = 0–60 °C, whereas the fully reflective condition

(R_{sol} = 100%, A_{sol} = 0%, dashed blue line) would remain at -17–5.4 °C. b,c) Estimated temperatures for RC and LRT under (b) Sub-Arctic and (c) Tropical sky transmission conditions (non-ideal, lower transmittance than in (a) Mauna Kea). For LRT, the temperature band shown reflects R_{sol} variation to keep the target range when sky transmittance degrades—highlighting the need for thermochromic (variable R_{sol}) behavior. d-f) Quantitative analysis of the power density components within the RC and LRT systems at the equilibrium states in (c),Tropical daytime. g–i) Quantitative analysis of the power density components within the RC and LRT systems at the equilibrium states in (a), Mauna Kea daytime. j) Nighttime simulations under zero solar radiation. LRT maintains a higher surface temperature than RC due to latent heating from atmospheric moisture adsorption. k,l) Quantitative analysis of the power density components within the RC and LRT system at the equilibrium states in (j).

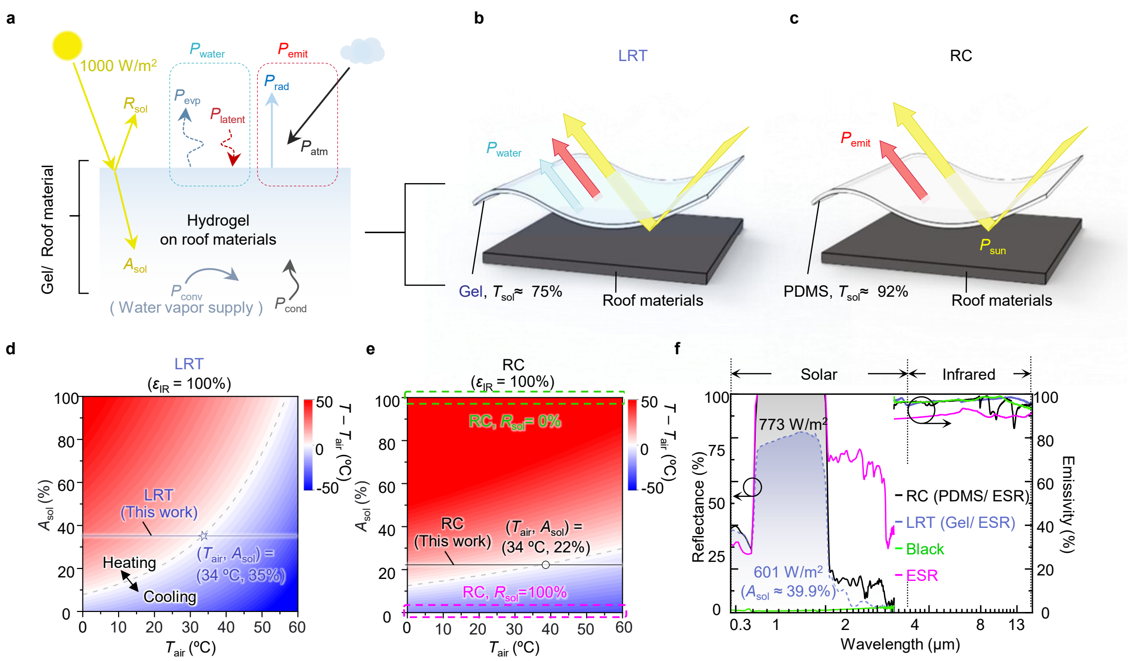

Figure S5. Energy balance modeling and optical properties of RC and LRT on different roofing materials. a) Comprehensive energy balance model for LRT applied to roof coatings. This model includes parasitic heat gain terms from conduction (P_{cond}) and convection (P_{conv}), as well as net evaporative power (P_{water}), which is absent in RC. b,c) Simplified energy balance diagrams for (b) LRT and (c) RC. The temperature of each system is influenced by the optical and thermal properties of both top and substrate layers. For LRT, net evaporative power (P_{water}) is included; for RC, only radiative terms are considered. Typical solar transmittance (T_{sol}) is ~75% for LRT and ~92% for a 100 µm-thick PDMS-based RC. d,e) Simulated net temperature shift (cooling: negative, heating: positive) relative to ambient air as a function of solar absorptance (A_{sol}) and ambient temperature (T_{air}) for (d) LRT and (e) RC. Both models assume ideal mid-IR emissivity (ε_{IR} = 100%). Optimal solar reflectance (R_{sol}) for LRT and RC is 65% and 78%, respectively. f) Measured solar reflectance (R_{sol}) of various samples: black emitter (light green dashed line; R_{sol} = 1.1%), enhanced specular reflector (ESR) film (pink dashed line; R_{sol} = 89.7%), LRT on ESR (blue solid line; R_{sol} = 60.1%), and RC on ESR (dark blue solid line; R_{sol} = 77.3%). Since ESR shows 0% of transmission, A_{sol} to be calculated as 100% – R_{sol}. RC sample consists of 2 mm PDMS, while LRT is a fabricated Li-HPC-PAAm hydrogel.

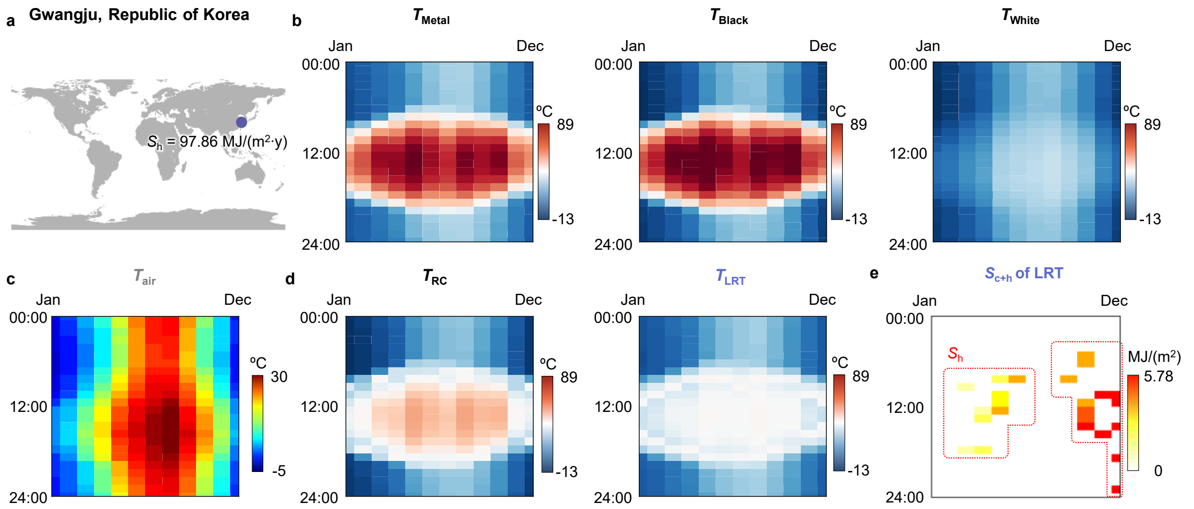

Figure S6. Hourly temperature profiles and space-conditioning energy savings for a representative climate zone. a) Annual total energy savings from heating (S_h) for LRT compared to conventional roof materials. b) Simulated surface temperatures of conventional roofs: metal (A_{sol} = 35%, ε_{IR} = 25%), black (A_{sol} = 95%, ε_{IR} = 95%), and white (A_{sol} = 5%, ε_{IR} = 95%). c) Hourly ambient air temperature (T_{air}) for the selected location. d) Simulated surface temperatures of a building prototype using RC (A_{sol} = 56%, ε_{IR} = 100%) and LRT (A_{sol} = 56%, ΔA_{sol} = 14%, ε_{IR} = 100%). e) Comparative figure of merit showing the source energy savings (S_c+S_h) of LRT relative to the best-performing conventional roof type across all conditions.

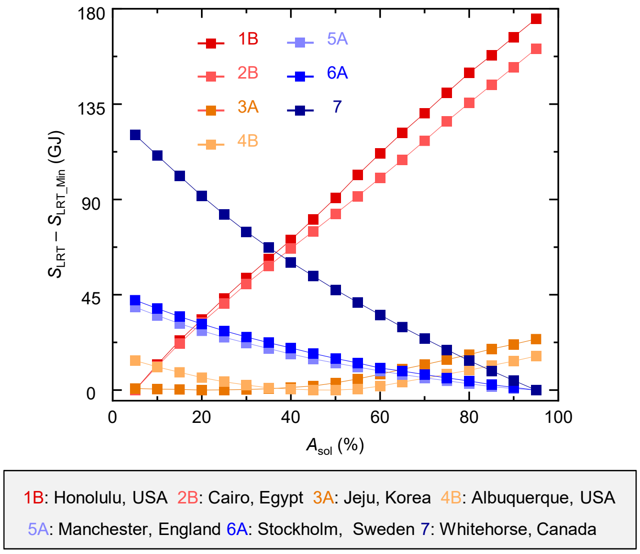

Figure S7. Theoretical analysis of space-conditioning energy consumption (S_{LRT}) of LRT across climatic zones as a function of A_{sol}. Space-conditioning energy consumption (S_{LRT}) of LRT was estimated across international climatic zones classified by American Society of Heating, Refrigerating and Air-conditioning Energies (ASHRAE) (e.g., 1B, 2B, 3A, etc.), using representative weather data. Each curve is offset by its minimum value (S_{LRT_Min}) to emphasize relative energy consumption trends. As the climate becomes colder, the slope of the S_{LRT} – S_{LRT_Min} curve transitions from negative to positive, indicating a stronger preference for higher A_{sol}. This suggests that an intermediate A_{sol} value yields optimal year-round performance, avoiding excessive cooling in cold zones and overheating in hot zones. The experimentally determined A_{sol} of the LRT (~40%) aligns well with the optimal range for mid-latitude climates such as Gwangju, Republic of Korea (35.17°N, 126.88°E).

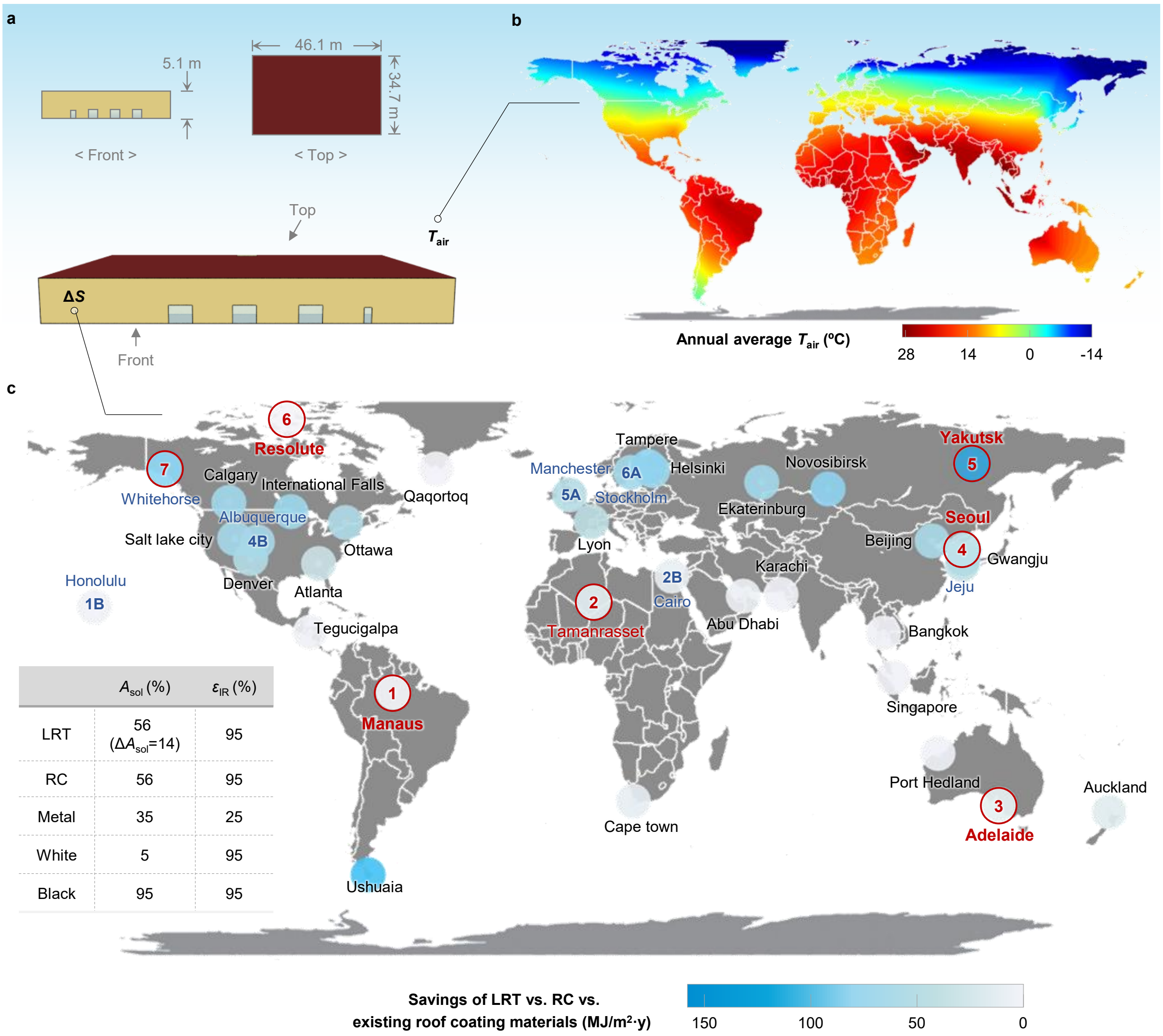

Figure S8. Simulated thermal regulation and energy-saving performance of LRT across global climates. a) Geometric configuration of the independent retail building model used for annual energy simulations, following standard building prototype guidelines. b) Global map showing annual average T_{air} across major cities used in the simulation. c) Estimated annual space-conditioning energy savings (ΔS_{min}) of LRT compared to RC and other commercial roof coatings for representative cities worldwide. Seven cities are marked with numerical labels, corresponding to Figure S7 in the Supporting Information, and arranged from highest to lowest average T_{air}.

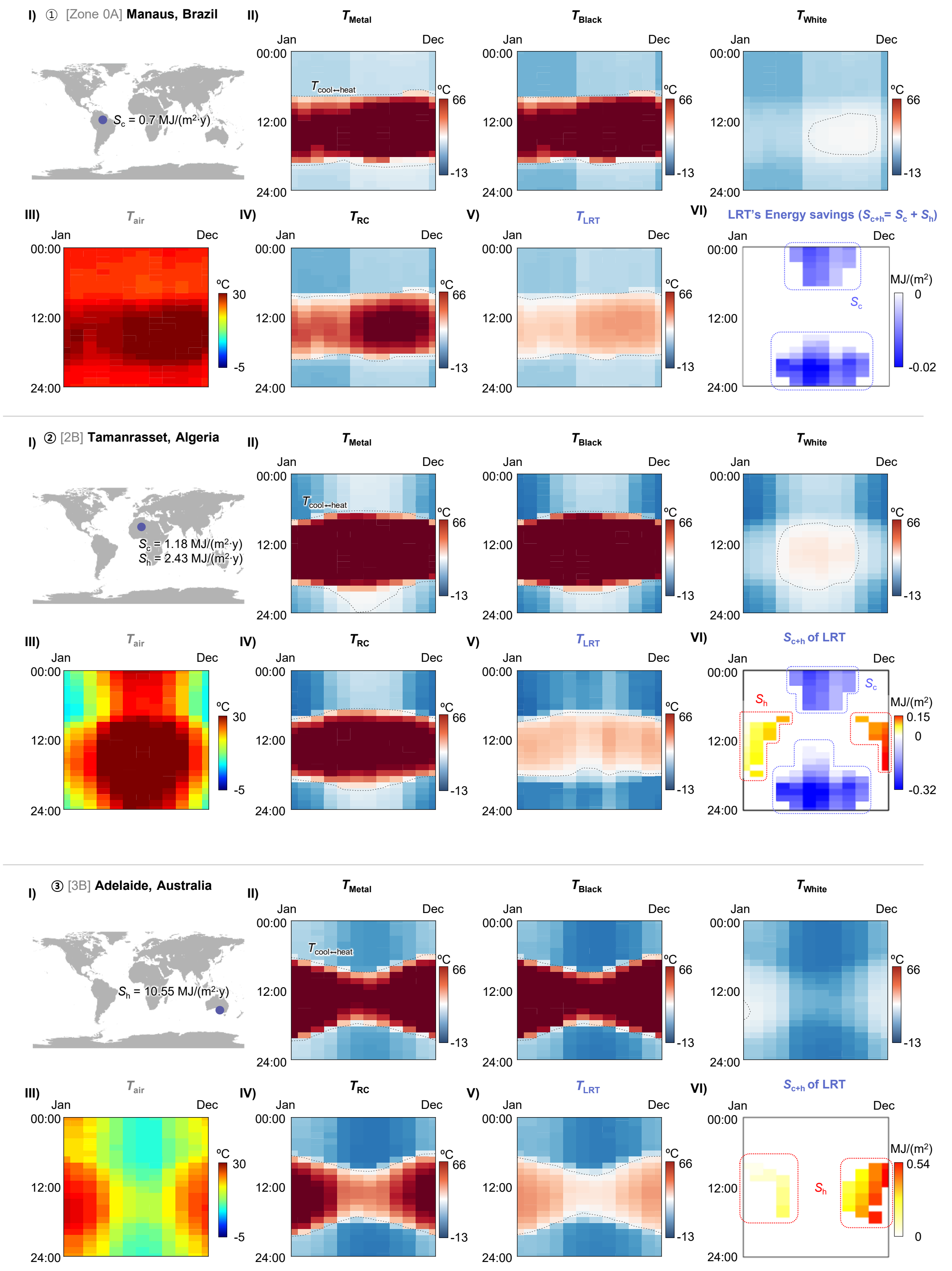

Figure S9. Hourly temperature and energy savings in cooling (S_c) and heating (S_h) for a representative region (S_{c+h}). I) Annual total energy savings from cooling (S_c) and heating (S_h) for LRT compared to conventional roof materials. II) Simulated surface temperatures of conventional roofs: metal ($A_{sol} = 35\%$, $\varepsilon_{IR} = 25\%$), black ($A_{sol} = 95\%$, $\varepsilon_{IR} = 95\%$), and white ($A_{sol} = 5\%$, $\varepsilon_{IR} = 95\%$). III) Hourly ambient air temperature (T_{air}) for the selected location. IV) Simulated surface temperatures of a building prototype using RC ($A_{sol} = 56\%$, $\varepsilon_{IR} = 100\%$) and LRT

(A_{sol} = 56%, ΔA_{sol} = 14%, ε_{IR} = 100%). V) Comparative figure of merit showing the source energy savings (S_c+S_h) of LRT relative to the best-performing conventional roof type across all conditions. (continued)

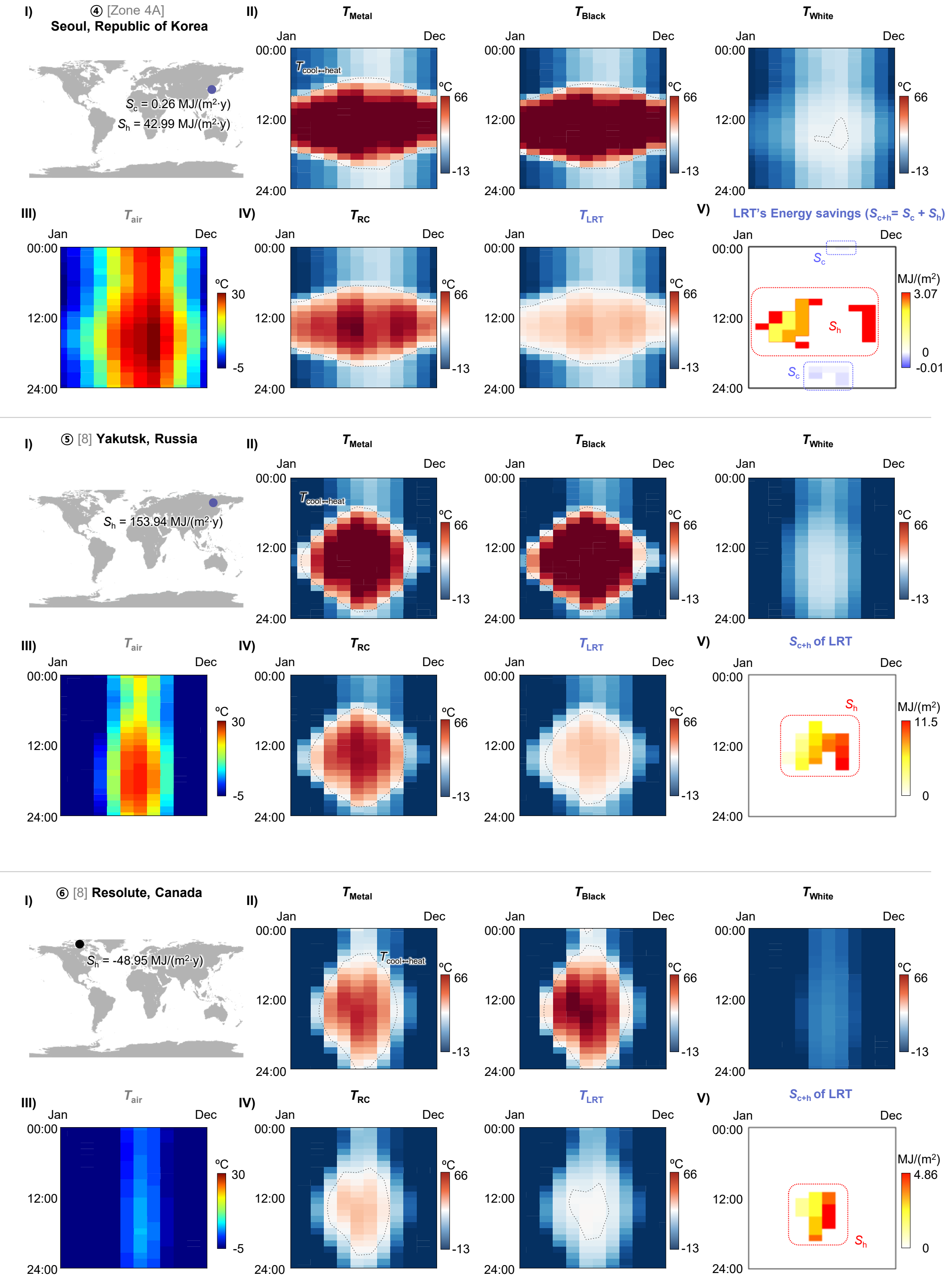

Figure S9. Hourly temperature and energy savings in cooling (S_c) and heating (S_h) for a representative region (S_{c+h}). I) Annual total energy savings from cooling (S_c) and heating (S_h) for LRT compared to conventional roof materials. II) Simulated surface temperatures of conventional roofs: metal (A_{sol} = 35%, ε_{IR} = 25%), black (A_{sol} = 95%, ε_{IR} = 95%), and white (A_{sol} = 5%, ε_{IR} = 95%). III) Hourly ambient air temperature (T_{air}) for the selected location. IV) Simulated surface temperatures of a building prototype using RC (A_{sol} = 56%, ε_{IR} = 100%) and LRT

(A_{sol} = 56%, ΔA_{sol} = 14%, ε_{IR} = 100%). V) Comparative figure of merit showing the source energy savings (S_c+S_h) of LRT relative to the best-performing conventional roof type across all conditions.

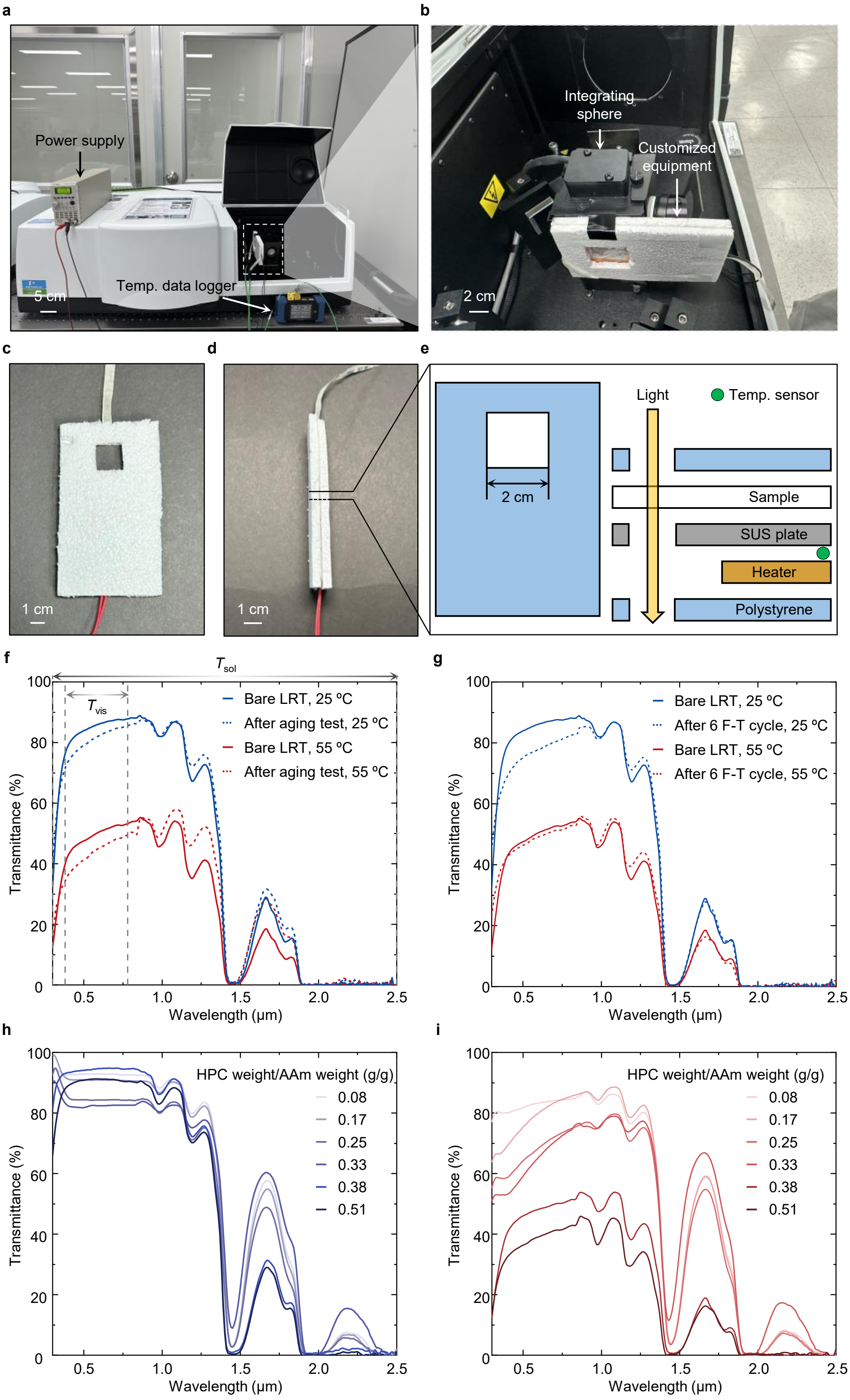

Figure S10. Experimental setup for optical properties of LRT. a) Photograph of the measurement setup used for optical characterization of LRT under controlled temperatures. b) Close-up image of the custom-made measurement setup, showing integration with temperature control and spectrometer. c,d) (c) Top view and (d) cross-sectional view of the optical setup, highlighting sample alignment and light path. e) Schematic illustrations of the measurement setup: top view (left) and exploded diagram (right) showing key components, including

heating stage, sample holder, and spectrometer coupling. f,g) Optical stability of LRT after accelerated aging tests as a function of temperature. f) Transmittance spectra of LRT before (solid line) and after the aging test (dashed line), in which the sample was exposed to 70 °C and 90% RH for 15 h. g) Transmittance spectra of LRT before (solid line) and after six freeze–thaw (F–T) cycles (dashed line), where one cycle is set as -25 °C for 2 h followed by 25 °C for 2 h. Slight variations in visible (T_{vis}; 0.38–0.78 μm) and solar-range (T_{sol}; 0.28–2.5 μm) transmittance were observed, indicating stable optical performance after accelerated aging tests. h,i) Transmittance spectra of LRT (h) below and (i) above the LCST for various HPC/AAm weight ratios. Increased HPC content leads to larger modulation in T_{vis} and T_{sol}.

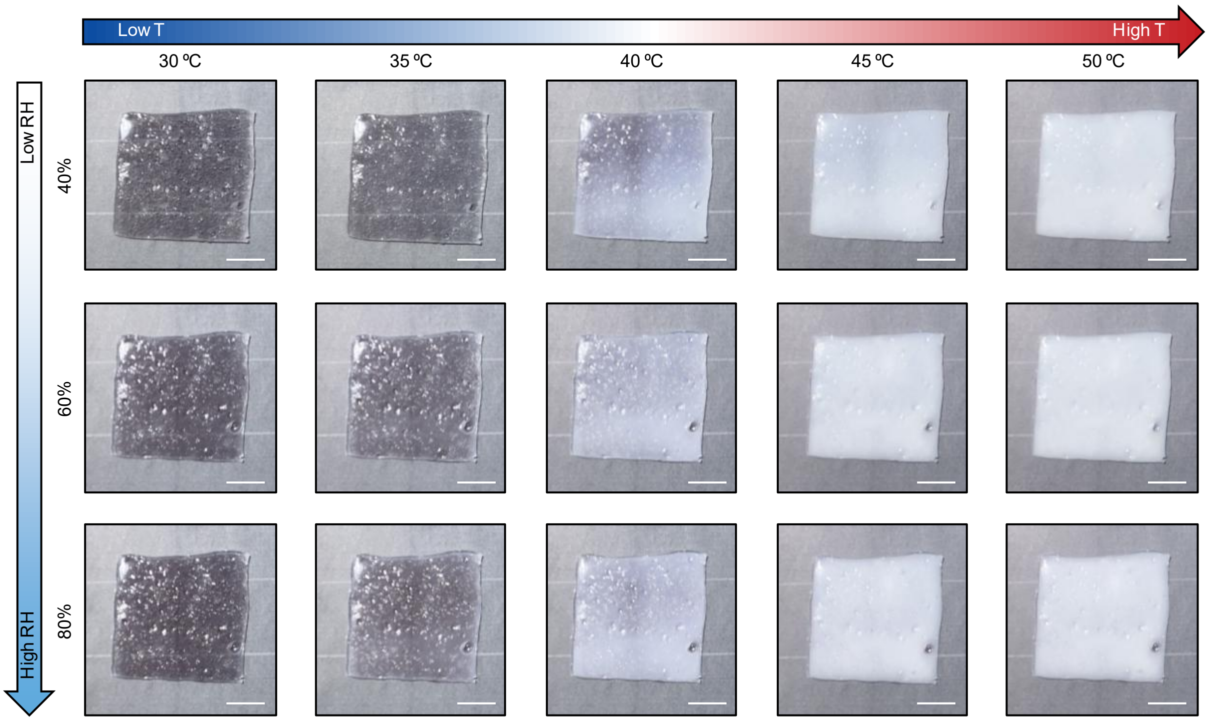

Figure S11. Photographs of the LRT under different temperatures and RH conditions. The LRT exhibits distinct thermochromic behavior with temperature, while showing negligible optical variation across varying RH. Scale bar: 1 cm.

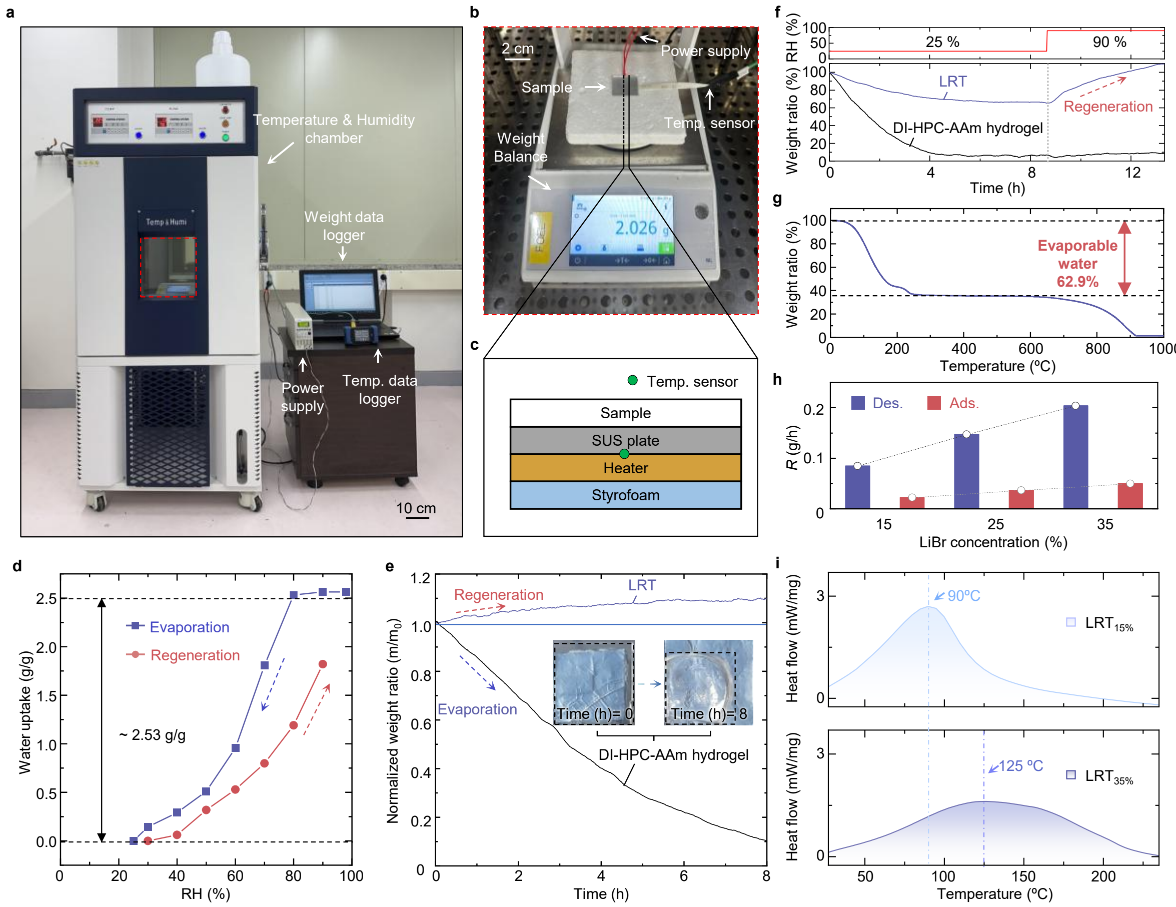

Figure S12. Mass transfer behavior and water management characteristics of the LRT. a) Photograph of the measurement setup used to evaluate water vapor sorption isotherms and weight variation. b) Close-up view of the customized chamber used for humidity-controlled experiments. c) Cross-sectional schematic of the setup showing layer configuration: sample, stainless steel (SUS) plate, heater, and polystyrene insulation. Environmental conditions were maintained at 25 °C and 70% RH. d) Water sorption isotherms of LRT at 25 °C, indicating hygroscopic behavior as a function of RH. e) Normalized weight variation of DI-HPC-AAm hydrogel (black) and LRT (blue) under identical conditions. LRT slightly gains weight due to moisture uptake, while DI-HPC-AAm hydrogel exhibits continuous water loss over 8 hours. f) Weight variations of DI-HPC-AAm hydrogel (black) and LRT (blue) under controlled humidity conditions (red). g) Thermogravimetric analysis (TGA) curve of LRT, showing that evaporable water constitutes 62.9% of total weight, representing the free water available for latent heat regulation. h) Water desorption and adsorption rates of LRTs with varying LiBr contents, corresponding to the dynamic cycling behavior shown in Figure 3d. i) Differential scanning calorimetry (DSC) curves of LRTs with 15% and 35% Li^+ concentrations. The lower-Li^+ sample exhibits an earlier evaporation peak (90 °C), while the higher-Li^+ sample evaporates at a higher temperature (125 °C), indicating delayed water release.

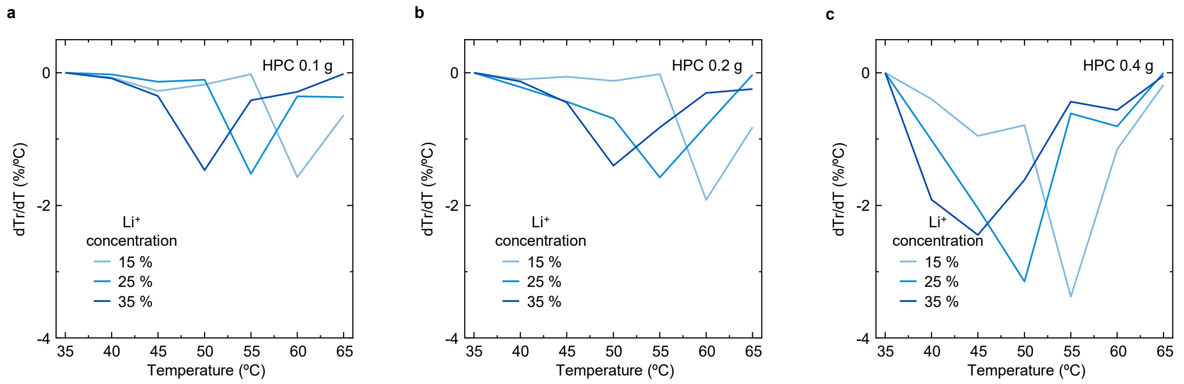

Figure S13. First derivative of the Tr-T curve of LRT measured at a wavelength of 500 nm with varying Li^+ and HPC concentrations: a) HPC = 0,1 g, b) HPC = 0.2 g, and c) HPC = 0.4 g. The LCST decreases as the Li^+ concentration increases regardless of HPC concentrations.

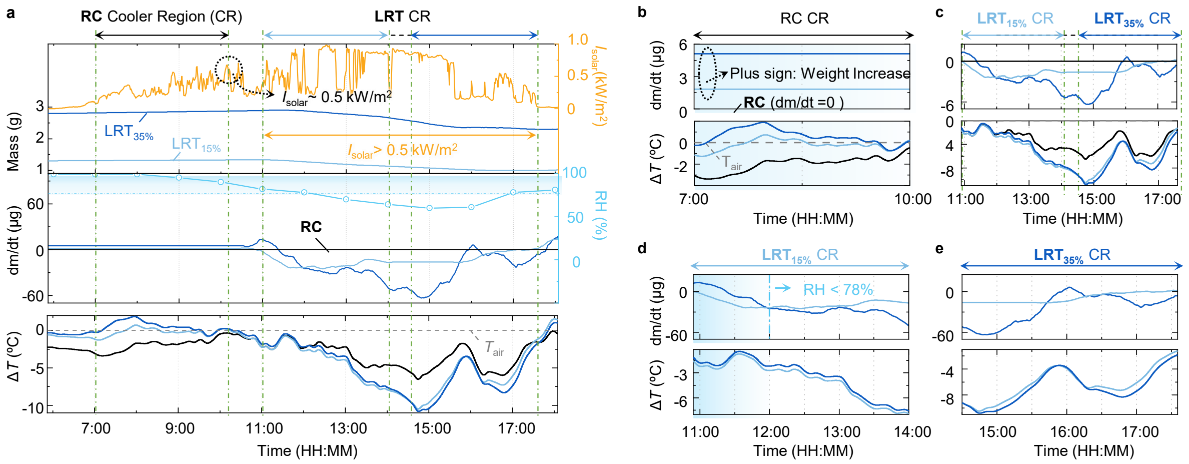

Figure S14. Optimization of Li$^+$ ion concentration in LRT for different humidity conditions. a) Daily surface temperature profiles of RC (gray dashed line), LRT$_{15\%}$ (sky blue), and LRT$_{35\%}$ (blue) under outdoor conditions. Regions where RC is cooler are marked as RC cooling region (RC CR), and regions where LRT is cooler are marked as LRT cooling region (LRT CR). b) Temperature and weight profiles during nighttime dew harvesting. RC shows the lowest temperature due to strong radiative cooling, while LRT gains mass (positive dm/dt) from atmospheric moisture. c) As solar irradiance (I_{solar}) increases during the day, LRT begins evaporating water (negative dm/dt), resulting in superior cooling performance over RC. d) Under subhumid conditions (RH > 78%), LRT$_{15\%}$ provides stronger cooling due to earlier evaporation onset. e) In contrast, under arid conditions (RH < 78%), LRT$_{35\%}$ exhibits better cooling performance due to greater water retention and higher latent heat release.

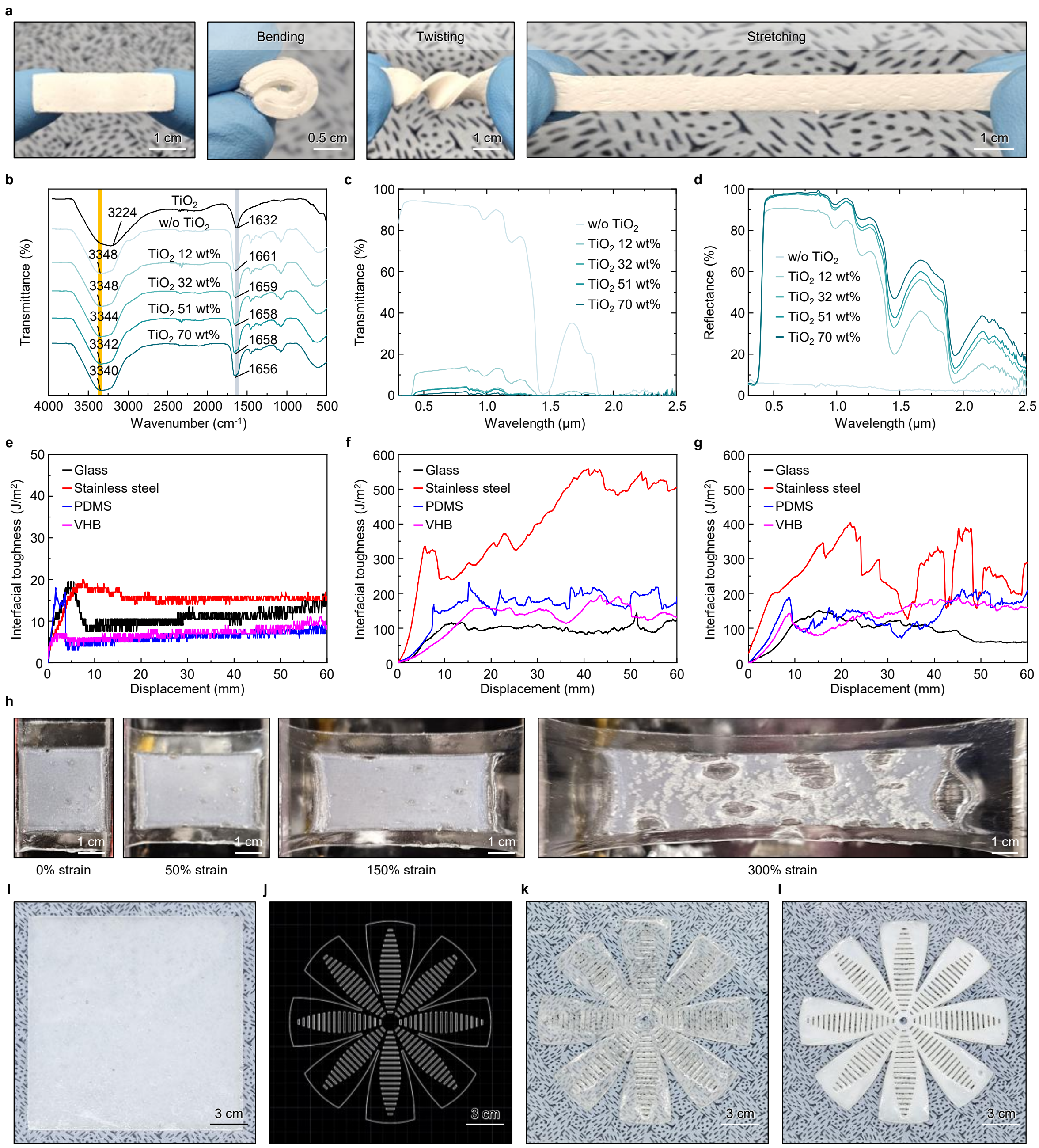

Figure S15. Mechanical flexibility and application-specific adaptatability of LRT. a) Photographs of TiO_2-reinforced LRT (32 wt% TiO_2 relative to AAm) under various mechanical deformations: undeformed, bent, twisted, and stretched, demonstrating high flexibility. b) FTIR spectra of pure TiO_2, pure PAAm, and LRTs with different TiO_2 contents (12–70 wt%). With increasing TiO_2 content, the N–H/O–H stretching band shifts from 3348 to 3340 cm^{-1}, while the C=O band shifts from 1661 to 1656 cm^{-1}. c,d) (c) Transmittance and (d) reflectance spectra of LRTs with different TiO_2 concentrations. e)Interfacial toughness of LRT adhered to untreated substrates: glass, stainless steel, PDMS, and VHB. f) Interfacial toughness of LRT on the same substrates after surface modification, showing significant improvement. g) Interfacial toughness of LRT after heating to 70 °C (above LCST), confirming adhesion retention under thermal stress. h) Stretching test of LRT on surface-treated VHB; delamination initiates at ~300% strain, indicating strong interfacial bonding. i) Photograph of a 20×20 cm LRT sheet prepared for integration onto curved surfaces. j) Laser-cut kirigami pattern enabling out-of-plane deformation.

k,l) Photographs of kirigami-patterned LRT below (k) and above (l) LCST, showing reversible transparency and flexibility during thermal actuation.

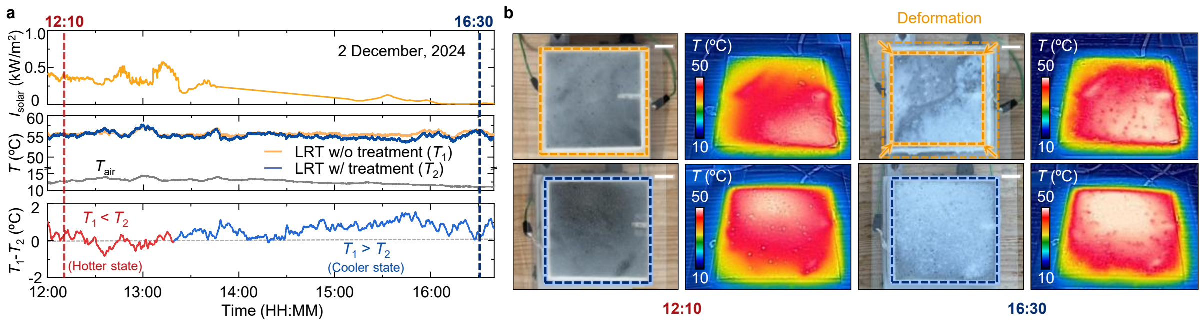

Figure S16. Outdoor area retention and cooling performance of surface-treated LRT. a) Outdoor measurements comparing LRT samples without surface treatment (orange line) and with surface treatment (blue line), tested on a hot plate under sunlight. Top: solar irradiance (I_{solar}); Middle: surface temperature profiles of LRT with and without surface treatment; Bottom: temperature difference between treated and untreated LRT. Improved adhesion from surface treatment minimizes deformation, leading to enhanced cooling stability. b) Photographic and infrared (IR) images of untreated (top) and surface-treated (bottom) LRT during field testing. The treated sample retains better contact area with the substrate, contributing to more uniform cooling. Scale bar: 2 cm.

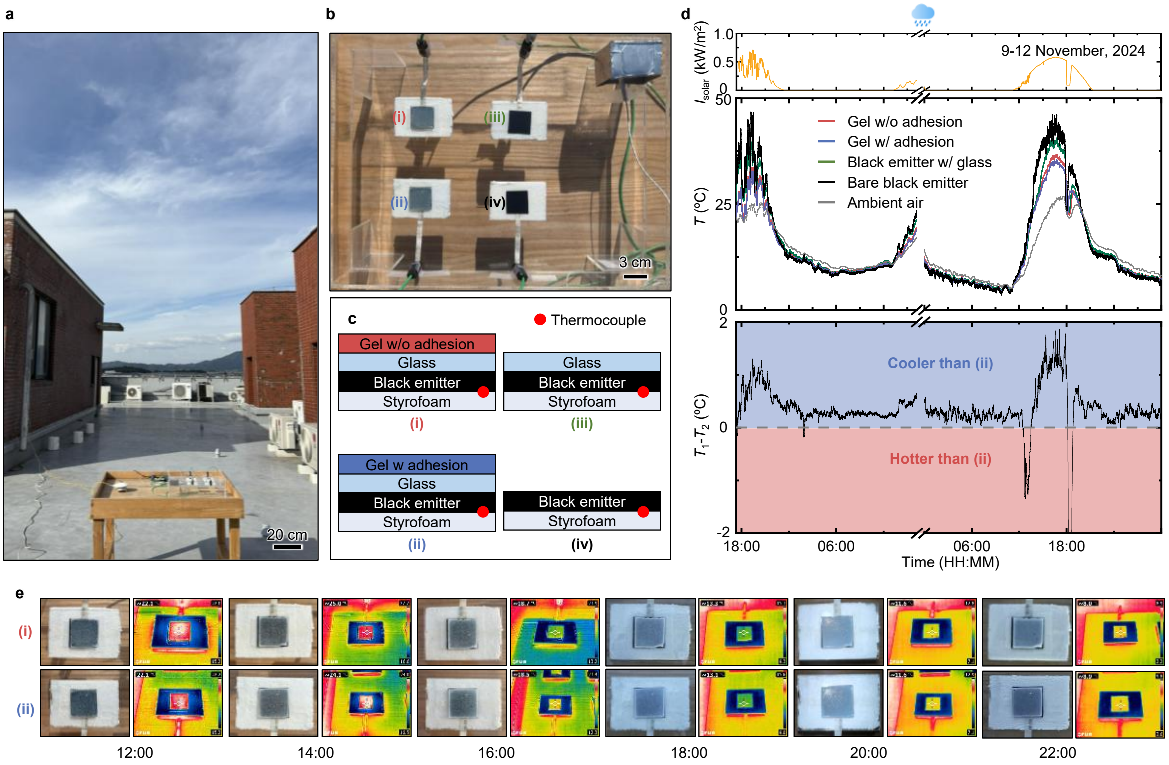

Figure S17. Long-term thermal regulation performance and adhesion durability of surface-treated LRT. a) Photograph of the full outdoor measurement setup used for the area retention and durability test. b,c) (b) Photograph and (c) schematic diagram of the test configuration. A black emitter substrate is used to simulate high-absorption roofing conditions. d) Two-day continuous measurements. Top: I_{solar}. Middle: surface temperatures of (i) surface-treated LRT (red), (ii) untreated LRT (blue), (iii) glass (green), (iv) bare black emitter (black), and ambient air (gray). Bottom: temperature difference between treated and untreated LRT (T_1–T_2). The untreated LRT gradually loses thermal contact, resulting in reduced cooling performance over time. e) Photographic and infrared (IR) images showing physical and thermal differences between (i) treated and (ii) untreated LRT samples during the test period. Treated LRT retains contact and area more effectively, leading to more stable and efficient thermal regulation.

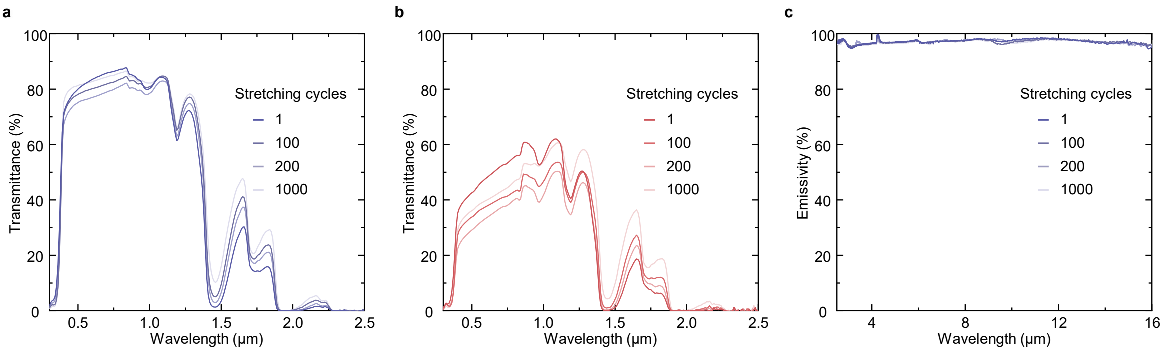

Figure S18. Optical stability of LRT under mechanical deformation. a,b) Transmittance spectra of LRT measured after various stretching cycles (1, 100, 200, 1000), (a) below LCST (25 °C) and (b) above LCST (55 °C). After 1000 cycles, changes in T_{sol} and T_{vis} remained moderate, with ΔT_{sol} = 33.4% and ΔT_{vis} = 43.2%, confirming sustained optical modulation performance. c) Emissivity spectra of LRT below LCST after different stretching cycles, showing consistently high ε_{IR} (>96%) despite repeated deformation.

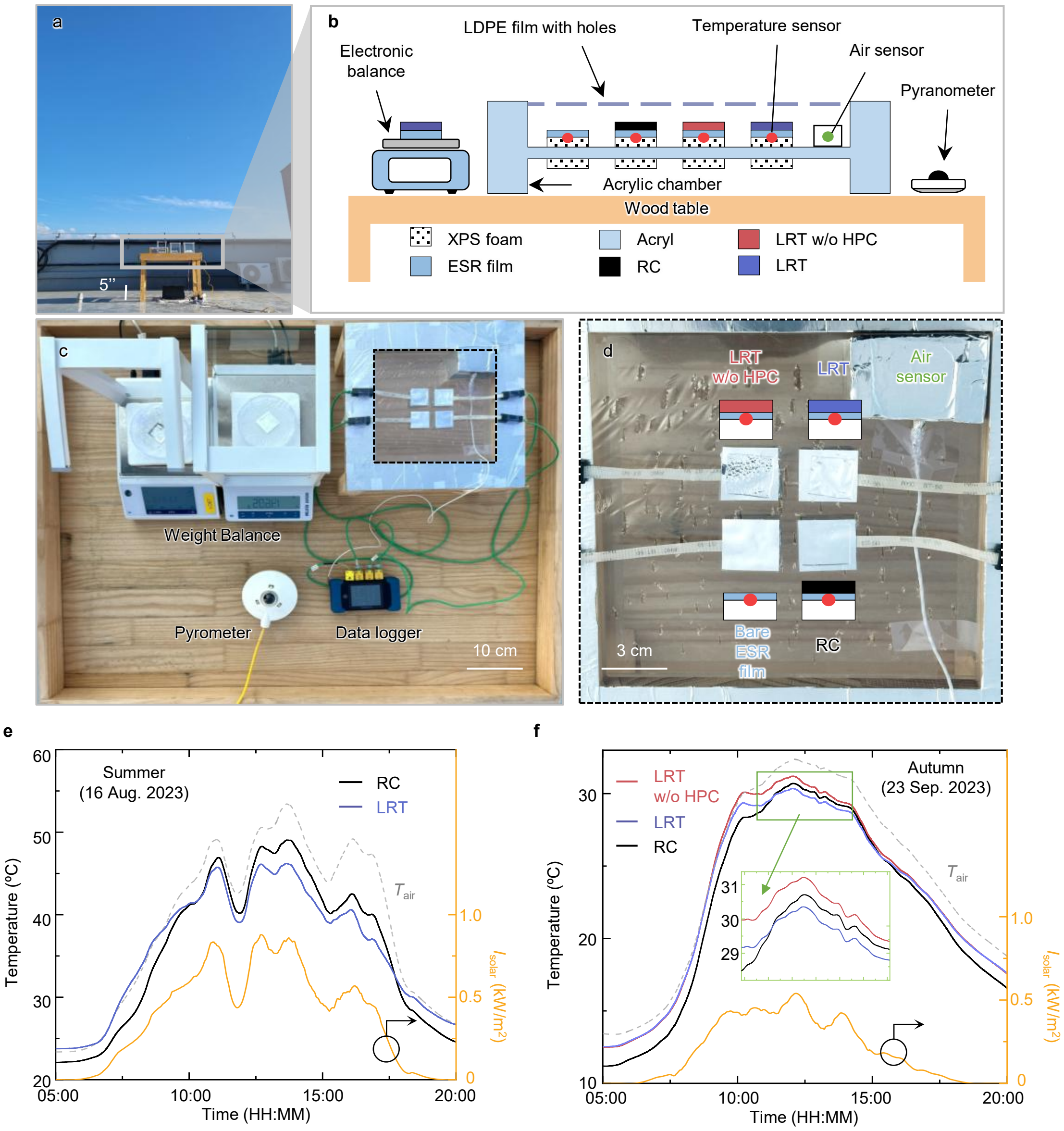

Figure S19. Outdoor thermal regulation performance of LRT on a cold substrate (ESR film). a) Photograph of the full outdoor measurement setup configured for cold substrate testing. b,c) (b) Schematic illustration and (c) corresponding photograph of the setup, where ESR film is used as the underlying substrate to minimize solar heat gain. d) Temperature measurement setup for surface and ambient air under cold substrate conditions. e,f) Real-time surface temperature measurements of RC, non-thermochromic LRT (without HPC), full LRT, and ambient air during outdoor tests in Gwangju, Republic of Korea (35.17°N, 126.88°E) in (e) summer and (f) autumn. In summer ($T_{air,\ avg}$ > 20 ºC), full LRT shows superior cooling compared to both RC and non-thermochromic LRT. Similar trends persist in autumn, indicating that the thermochromic component enhances passive temperature regulation across seasons.

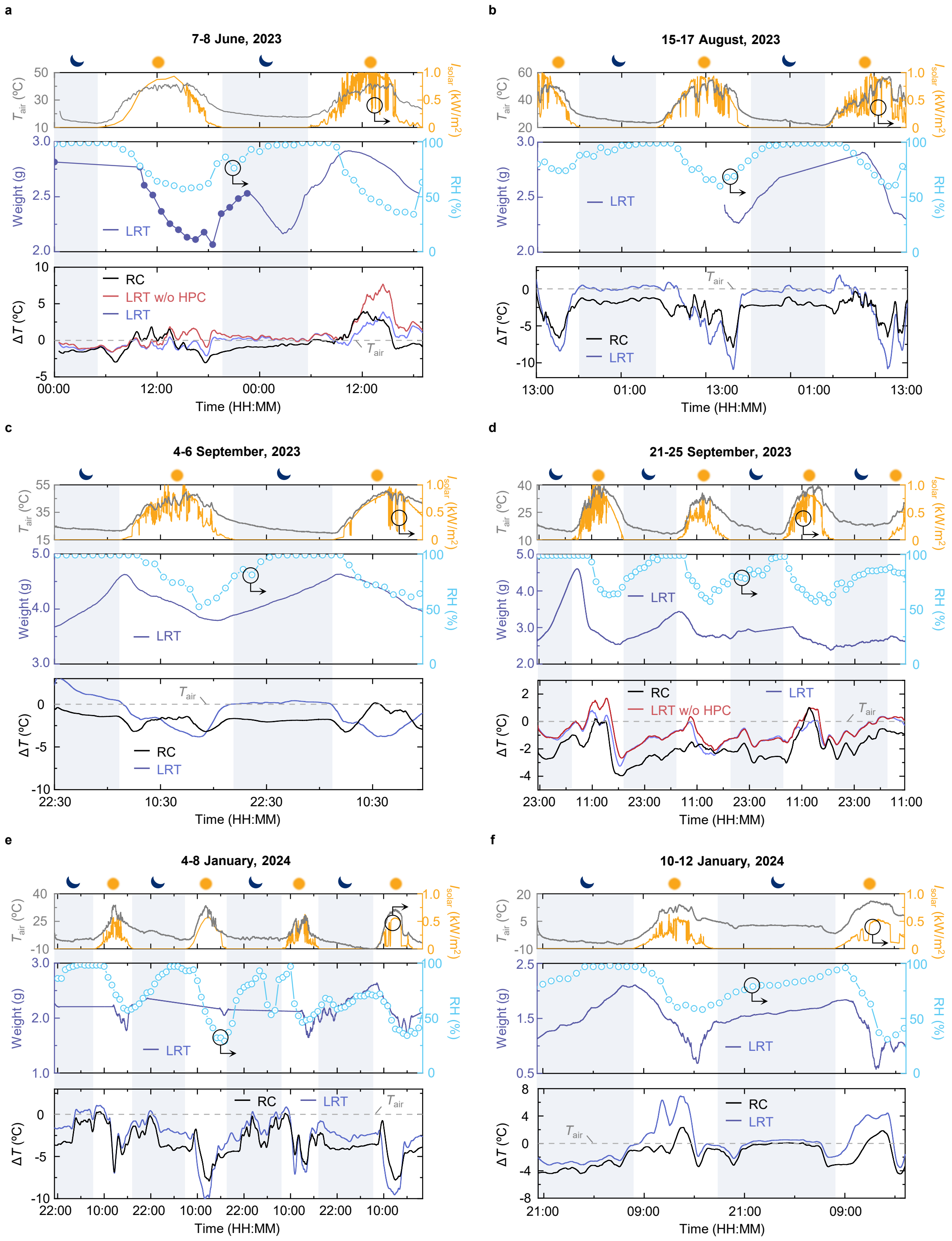

Figure S20. Outdoor thermal regulation performance of LRT under varying weather conditions in Gwangju, Republic of Korea (35.17°N, 126.88°E). a-f) Time-series measurements conducted under natural weather fluctuations include: Top, solar irradiance (I_{solar}) and ambient air temperature (T_{air}, gray dashed line); Middle, mass variation of LRT; Bottom, continuous temperature differences between T_{air} and surface temperatures of RC, LRT without HPC, and LRT. The results highlight the distinct contributions of evaporative cooling and thermochromic switching under dynamic solar and atmospheric conditions.

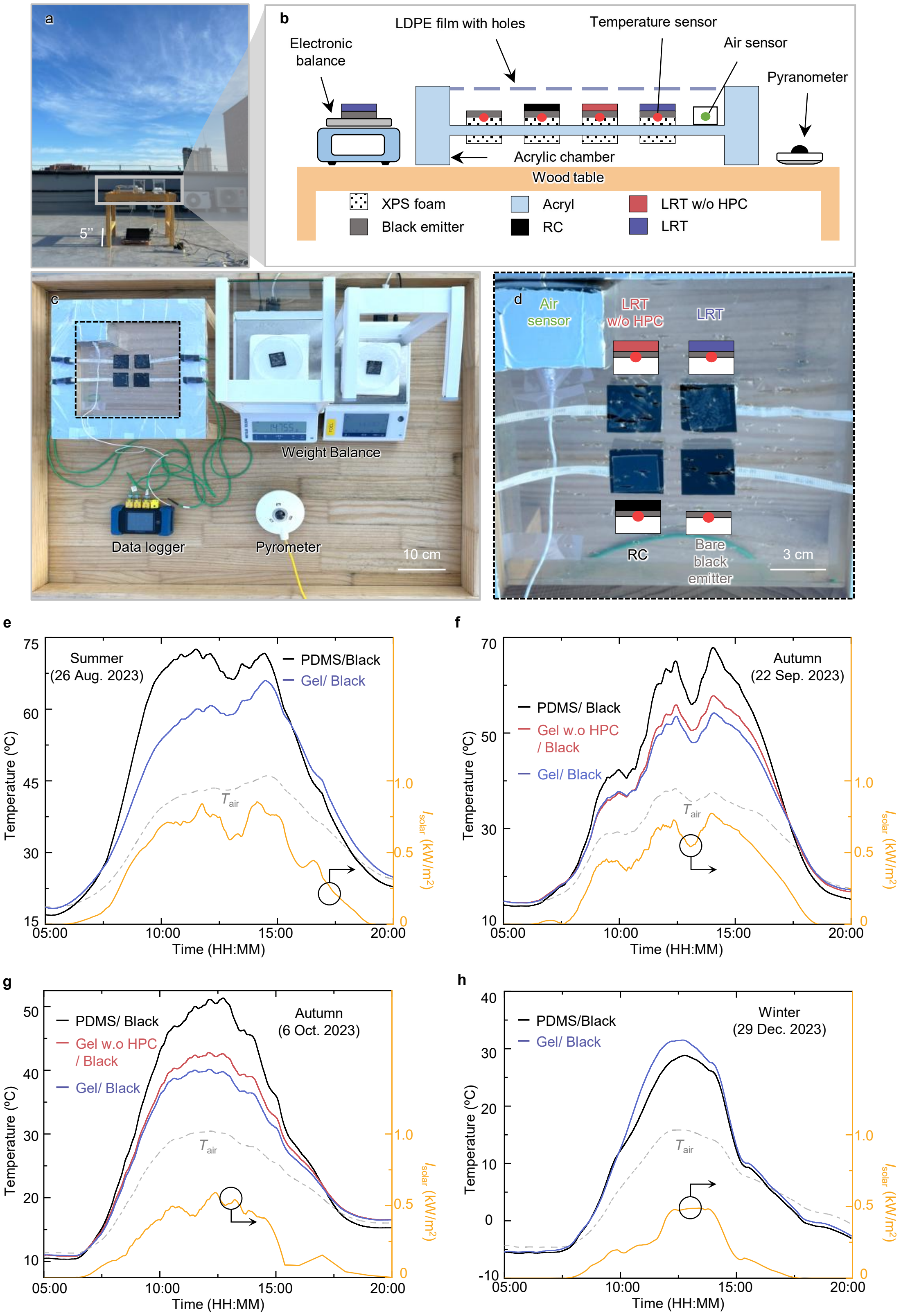

Figure S21. Outdoor thermal regulation performance of LRT on a hot substrate (black emitter). a) Photograph of the complete outdoor test setup configured with a black emitter as the substrate to simulate high heat input. b,c) (b) Schematic and (c) photograph of the experimental setup. Weight variation of LRT was monitored using a precision balance. d) Temperature measurement configuration. Samples were mounted on XPS foam blocks to minimize thermal conduction from surroundings. The chamber was enclosed with LDPE film containing holes to

allow water vapor escape. e-h) Real-time surface temperature measurements of black roof (RC), non-thermochromic LRT, full LRT, and ambient air under hot substrate conditions in Gwangju, Republic of Korea (35.17°N, 126.88°E) during (e) summer, (f,g) autumn, and (h) winter. In hot conditions ($T_{air, avg}$ > 20 ºC), LRT shows superior cooling compared to RC and non-thermochromic LRT, owing to combined radiative, evaporative, and thermochromic effects. During colder conditions ($T_{air, avg}$ < 20 ºC), LRT exhibits enhanced heating performance due to its moisture adsorption and latent heat retention.

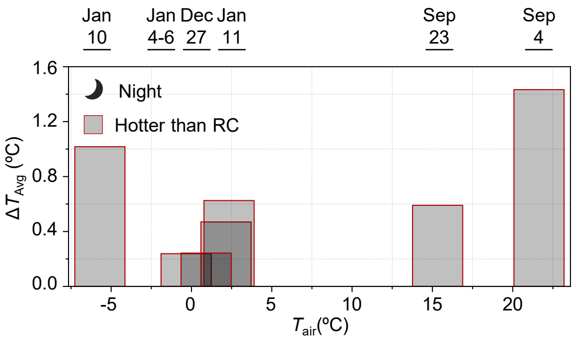

Figure S22. Nighttime surface temperature comparison between LRT and RC. Bar graph showing the average temperature difference (ΔT_{avg}) between LRT and RC during nighttime outdoor measurements. In all cases, LRT maintains a higher temperature than RC, attributed to latent heat release from atmospheric moisture adsorption.

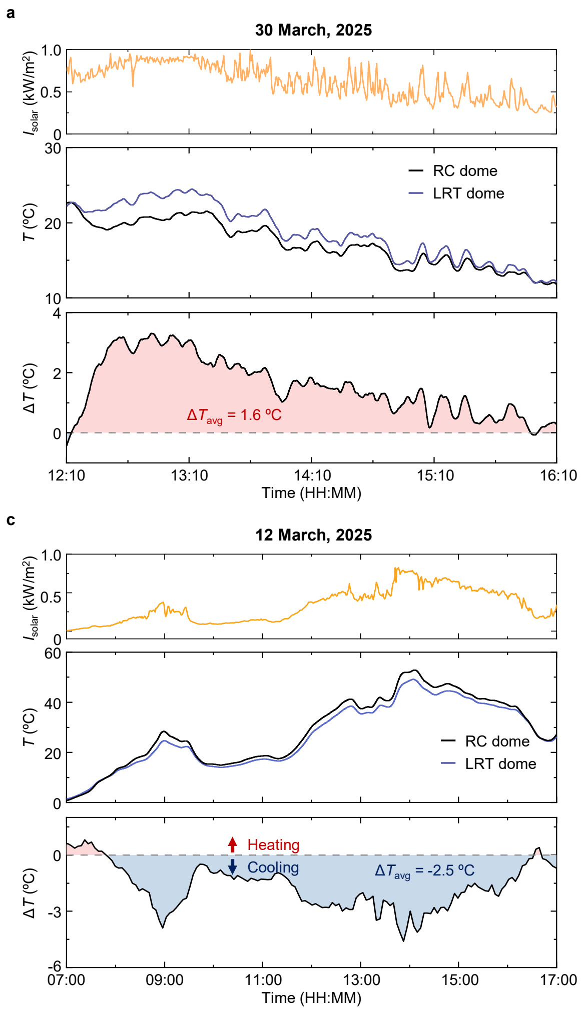

Figure S23. Outdoor thermal regulation performance of LRT dome under different substrate conditions. a-d) Daytime surface temperature measurements over four consecutive days for RC and LRT domes mounted on two types of substrates: (a,b) ESR film and (c,d) black emitter. LRT consistently shows enhanced heating on ESR (low-temperature substrate) and superior cooling on black emitter (high-temperature substrate), confirming adaptive thermal regulation based on internal heat source conditions.

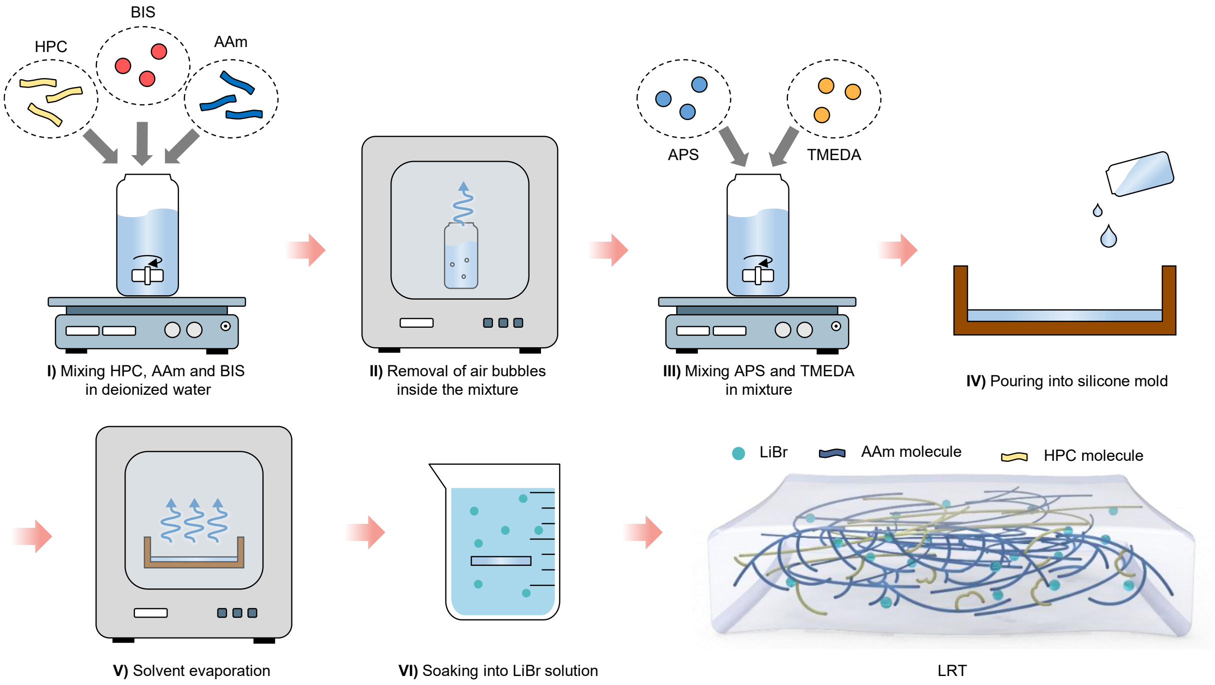

Figure S24. Schematic of fabrication process of LRT. I) Hydroxypropyl cellulose (HPC; thermoresponsive material), acrylamide (AAm; backbone polymer), and N,N′-methylenbisacrylamide (BIS; crosslinking agent) were mixed with 10 mL of deionized water and stirred at 350 rpm for 4 hours. II) The mixture was loaded into a vacuum chamber and kept overnight to eliminate air bubbles inside the mixture. III) Ammonium persulfate (APS; initiator) and N,N,N′,N′-tetramethylethylenediamine (TMEDA; accelerator) were added into the mixture and stirred at 250 rpm for 1 min before gelation. IV) The mixture was poured into pre-prepared silicone mold. V) Solvent inside the hydrogel evaporated at 70ºC overnight to form porous structure of backbone polymer. VI) The dehydrated hydrogel was soaked into LiBr solution (35%) until it was completely swollen.

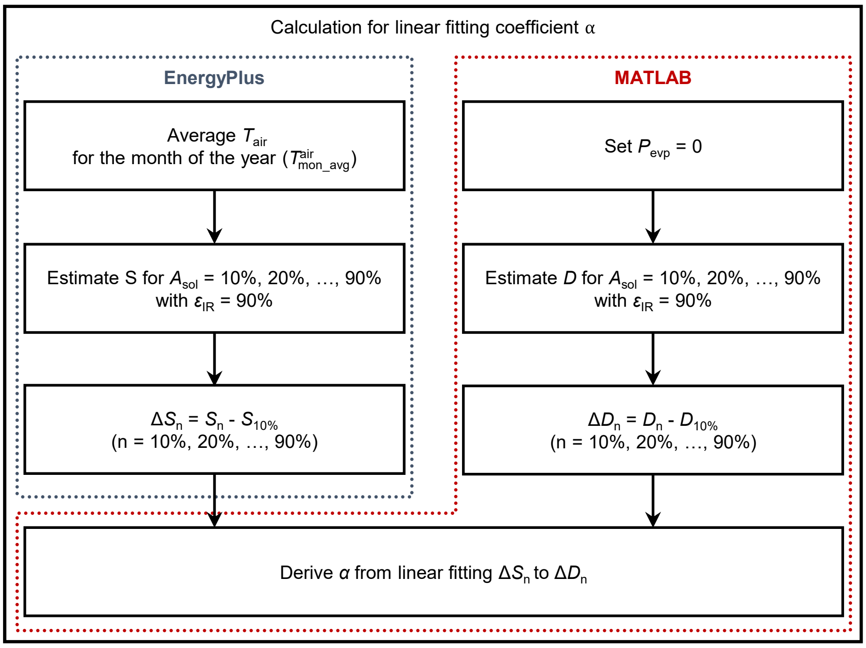

Figure S25. Flowchart for estimating linear fitting coefficients (α_h and α_c) used in LRT energy savings analysis. Schematic representation of the procedure for deriving the heating (α_h) and cooling (α_c) energy conversion coefficients through parametric simulations of static roof materials with varying solar absorptance (A_{sol}) and fixed infrared emissivity (ε_{IR} = 90%). The resulting coefficients are used to convert annual heating and cooling degree reductions into source energy savings in the energy simulation framework.

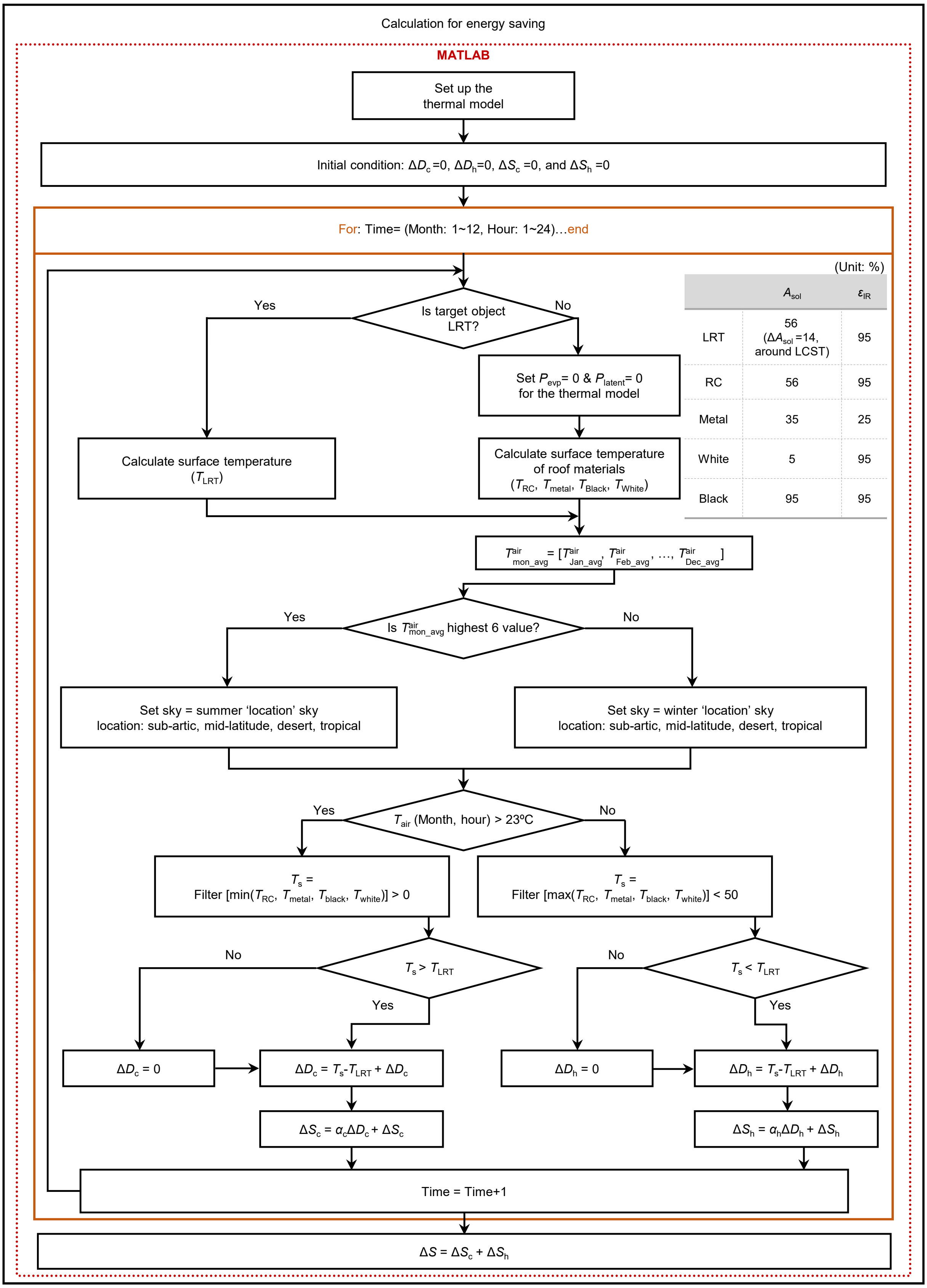

Figure S26. Flowchart of calculation process for evaluating energy saving of LRT. Step-by-step schematic of the computational framework used to evaluate the space-conditioning source energy savings (SCSES) of LRT. The process includes hourly surface temperature simulation, calculation of heating and cooling degree hours,

application of linear conversion factors (α_h and α_c), and comparison with baseline roofing materials to derive SCSES and $SCSES_{min}$ values for global performance mapping.

Table S1. Recently-developed thermal regulation technologies. (continued)

Types	Methods (materials)	R_{vis} (%)	T_{vis} (%)	T_{sol} (%)	ε_{LWIR} (%)	Transition temperature (ºC)	Evaporation rate (mg cm^{-2} h^{-1})	Regeneration Time (h)	Cooling/Heating temperature (ºC) or power (W/m^2)	Ref
Passive thermochromism	Solar modulation by thermochromic material (HPC)	-	31.2-90.1	36.5 - 84.0	-	10 – 44 (@ PH 1 – 6)	-	-	9.1 ºC/ - (of sample on the Styrofoam box with sample roof, compare to glass roof @700 W IR lamp for 1 h)	[9]
Passive thermochromism	Solar modulation by thermochromic material (HPC)	-	-	0.4 – 87.2	-	21 – 30 (with $CaCl_2$ 2.2 – 1.1 g)	-	-	10 ºC/ - (of sample on the Styrofoam box with sample roof, compare to glass roof @125 W halogen lamp for 1 h)	[10]
Passive thermochromism	Solar modulation by thermochromic material (PNIPAM)	-	~1.2-~79.1	-	-	27.6 – 33.2 (adjustable with HEMA content)	-	-	22.0 ºC/ 12.5 ºC (of sample on the insulated box with sample, compare to black substrate and PVDF-HFP film)	[11]
Active evaporative cooling	Spontaneous water supply (PNIPAM)	-	-	-	-	35.7	~60	-	5.9/ 5.0 ºC (of 36.5 ºC normal body @ T_{air}= 50/ 5 ºC)	[12]
Passive evaporative cooling	Spontaneous water supply (LiBr)	-	-	-	-	-	9.7 @ 25 ºC, 60%	~2.5 @ 25 ºC, 60%	14 ºC/ - (of sample coated silicon(Si) solar cell, compare to bare Si cell @ RH 28%, T_{air}= 28 ºC, I_{solar}=0.7 kW/m^2)	[13]
Passive radiative cooling	IR modulation by phase transition material (VO_2, HfO_2)	-	-	-	21 - 82	27.5	-	-	196/ 50 W/m^2 (approximate value measured by IR camera @ Hot plate temp.= 60/ 0 ºC)	[14]
Passive radiative cooling	IR modulation by phase transition material (VO_2, Al_2O_3)	11 - 17	-	-	25 - 75	63.7 (cooling loop), 67.9 (heating loop)	-	-	20 / ~170 ºC (of sample compared to T_{air} @ A vacuum chamber with ZnS window)	[15]
Passive thermochromism	Solar modulation by thermochromic material (HPC)	10 - 19	8.1 - 70.3	7.7 - 58.6		31	-	-	5.4/ 0 ºC (of air inside the Styrofoam box with sample roof, compare to box with glass roof @ T_{air}= -7 ºC)	[16]
Active radiative cooling	IR modulation by flipping				10 - 95 (2.5 - 25 μm)					
Passive radiative cooling		> 90	-	-	76	-	46	-	9 ºC/ - (of ethylene glycol in a sample coated stainless steel container, compare to bare container @ T_{air}= 35-42 ºC, I_{solar}= 0.6-0.7 kW/m^2)	[17]
Active evaporative cooling	External water supply (P(VDF-TrFE), PAN)									

Table S1. Recently-developed thermal regulation technologies.

Types	Methods (materials)	R_{vis} (%)	T_{vis} (%)	T_{sol} (%)	ε_{LWIR} (%)	Transition temperature (ºC)	Evaporation rate (mg cm^{-2} h^{-1})	Regeneration Time (h)	Cooling/Heating temperature (ºC) or power (W/m^2)	Ref
Passive thermochromism	Solar modulation by thermochromic material (CM)	87.9 - 94.7	-	-		25	-	-	7.8 ºC/ - (of sample below T_{air} @ RH ~64%, T_{air}= ~33 ºC, I_{solar}=0.88 kW/m^2)	[18]
Passive radiative cooling					94					
Passive thermochromism	Solar, IR modulation using programmable structure (RC tape, Nano-Cr Al tape)	9 - 85	-	-		23 - 24	-	-	126 / 859 W/m^2 (of sample below T_{air} @ I_{solar} > 0.85 kW/m^2)	[19]
Passive radiative cooling					96					
Passive thermochromism	Solar modulation by thermochromic material (PNIPAM)	12 - 82	3.9 - 78.9	3.7 - 63.6		31	-		3.7/ 5.8 ºC (of sample compared to T_{air} @ RH ~38/ ~41%, T_{air} ~20 / ~32 ºC, I_{solar}=0.59/ 0.47 kW/m^2)	[20]
Passive radiative cooling					96					
Passive thermochromism	Solar modulation by refractive index difference (Porous SiO_2)	11.1 - 92.8	12.9 - 93.3	-		15	-	-	Maximally 5/ 10 ºC (of sample compared to T_{air} @ T_{air} ~34 / ~10 ºC, I_{solar}=0.9/ 0.4 kW/m^2)	[21]
Passive radiative cooling					93.6					
Passive thermochromism	Solar modulation by thermochromic material (PNIPAM)	5.9 – 91.6	-	-		32	-	-	Average -/19 ºC (of sample compared to T_{air} @ T_{air} ~10 ºC, I_{solar}= ~0.5 kW/m^2)	[22]
Passive radiative cooling					~95 (4 - 18 μm)					
Passive thermochromism	Solar modulation by thermochromic material (PNIPAM)	-	28.6 – 97.5	7.1 - 65.5		31	-	-	15 ºC/ - (of sample on the Styrofoam box with sample roof, compare to glass roof @ T_{air} ~28 ºC, 1 k W/m^2 solar simulator 40 min.)	[23]
Passive radiative cooling	IR mouldation by AgNW				35 - 92					
Passive thermochromism	Solar modulation by thermochromic material (W-VO_2)	-	-	20.12		32	-	-	3.8 ºC/ - (of sample on the Styrofoam box with sample roof, compare to glass roof @ RH ~38/ ~41%, T_{air} ~20 / ~32 ºC, I_{solar}=0.5 kW/m^2)	[24]
Passive radiative cooling	IR modulation by phase transition (PNIPAM)				35 - 68	27.5				
Passive radiative cooling	-	93	-	-	94				8 ºC/ - (of sample below conventional RC @ RH ~24%, T_{air}= ~35 ºC, I_{solar}=~0.7 kW/m^2)	[1]
Passive evaporative cooling	Spontaneous water supply ($CaCl_2$, CA)					Dew point	~80 @ 25 ºC, RH= 60%	~8 @ 25 ºC, RH= 98%		
Passive thermochromism	Solar modulation by TC material (HPC)	6 - 17.8	54.8 - 78.6	53.4 - 71.7		50			5.8/ 4.2 ºC (of sample compared to conventional RC @ RH ~60/ ~61%, T_{air} ~48 / ~16 ºC, I_{solar}=0.77/ 0.47 kW/m^2)	This work
Passive radiative cooling					96	-				
Passive evaporative cooling	Spontaneous water supply by absorbent (LiBr)					Dew point	68 @ 25 ºC, RH= 70%	~0.66 @ 25 ºC, RH= 70%		

Table S2. Estimated S_h and S_c of LRT in seven representative cities, compared to RC and conventional roof types. Simulated annual S_h and S_c of LRT across seven climate zones defined by ASHRAE. Representative cities include locations ranging from hot-humid to cold-dry climates.

ASHRAE climate zone	Sky	Location (latitude, longitude)	Average annual T_{air} (ºC) (T^{air}_{min}/T^{air}_{max})	α_c (MJ/(m^2·y·K))	α_h (MJ/(m^2·y·K))	S_c (MJ/(m^2·y))	S_h (MJ/(m^2·y))	S_{c+h} (MJ/(m^2·y))
1B	Desert	Honolulu, USA (21.3289, -157.9372)	25.25 (23.01/27.31)	0.83	0	0.49	0	0.49
2B	Desert	Cairo, Egypt (30.1256, 31.41)	23.19 (14.58/30)	0.94	0.07	3.62	1.81	5.43
3A	Mid-latitude	Jeju, Republic of Korea (33.5236, 126.5383)	16.71 (6.65/27.75)	0.64	1.22	0.15	28.15	28.30
4B	Mid-latitude	Albuquerque, USA (35.0408, -106.6253)	14.13 (1.91/26.25)	0.54	1.33	3.45	36.87	40.31
5A	Mid-latitude	Manchester, England (53.3567, -2.2681)	10.07 (4.55/16.18)	0.15	2.10	0	38.20	38.20
6A	Mid-latitude	Stockholm, Sweden (59.6339, 17.9567)	7.43 (-2.2/17.85)	0.2	2.61	0	52.41	52.41
7	Sub-arctic	Whitehorse, Canada (60.7158, -135.0703)	0.25 (-14.5/14.18)	0.09	2.95	0	76.50	76.50

Table S3. Global estimation of S_h and S_c of LRT across ASHRAE climate zones. Annual S_h and S_c of LRT compared to RC and conventional roofing systems, aggregated by ASHRAE-defined climate zones. The table summarizes the minimum and maximum energy savings achieved by LRT in each zone, reflecting its climate-dependent efficiency in both heating- and cooling-dominant regions.

ASHRAE climate zone	Sky	Location (latitude, longitude)	Average annual T_{air} (°C) (T^{air}_{min}/T^{air}_{max})	α_c (MJ/(m^2·y·K))	α_h (MJ/(m^2·y·K))	S_c (MJ/(m^2·y))	S_h (MJ/(m^2·y))	S_{c+h} (MJ/(m^2·y))
0A	Tropical	Bangkok, Thailand (13.9217, 100.6117)	29.08 (27.39/30.96)	0.92	0	1.48	0	1.48
0A	Tropical	Singapore, Singapore (1.3686, 103.9933)	27.86 (26.91/28.59)	0.75	0	0.30	0	0.30
0A	Tropical	Manaus, Brazil (-3.0428, -60.05)	26.514 (25.77/27.36)	0.92	0	0.70	0	0.70
0B	Desert	Abu Dhabi, U.A.E. (24.4439, 54.6517)	28.6 (19.51/36.27)	1.06	0.001	2.97	0	2.97
0B	Desert	Karachi, Pakistan (24.9117, 67.1683)	27 (19.26/31.45)	1.01	0.001	1.02	0.02	1.04
0B	Desert	Port Hedland, Australia (-20.3769, 118.6192)	26.394 (20.48/30.88)	1.01	0.002	1.18	0.04	1.22
2A	Tropical	Tegucigalpa, Honduras (14.0683, -87.2172)	22.06 (20.03/24.12)	0.89	0	1.72	0.01	1.73
2A	Mid-latitude	Nairobi, Kenya (-1.3206, 36.9356)	19.7 (17.62/21.24)	0.92	0.004	3.28	0.09	3.37
2B	Desert	Tamanrasset, Algeria (22.8033, 5.4494)	23.145 (13.86/30.65)	1.11	0.10	11.80	2.43	14.23
3A	Mid-latitude	Atlanta, USA (33.6189, -84.4478)	17.21 (5.82/26.64)	0.59	0.95	0.51	22.29	22.80
3A	Mid-latitude	Auckland, New Zealand (-37.0147, 174.8119)	15.73 (11.4/20.74)	0.51	0.45	0	9.86	9.86
3B	Desert	Adelaide, Australia (-34.9542, 138.5228)	17.02 (11.49/22.71)	0.62	0.47	0	10.55	10.55
3C	Mid-latitude	Cape Town, South Africa (-33.9525, 18.6033)	17.14 (12.91/21.66)	0.70	0.25	0	6.05	6.05
4A	Mid-latitude	Gwangju, Republic of Korea (35.1667, 126.8833)	14.15 (0.77/26.97)	0.60	1.75	0.24	41.99	42.23
4A	Mid-latitude	Lyon, France (45.7325, 4.9408)	13.15 (3.67/22.97)	0.43	1.53	0.99	31.45	32.44
4A	Mid-latitude	Seoul, Republic of Korea (37.5744, 126.97639)	13.08 (-14.5/14.18)	0.59	1.79	0.26	42.99	43.25
4A	Mid-latitude	Beijing, China (40.0689, 126.9764)	13.06 (-3.7/27.43)	0.60	1.80	1.20	45.09	46.29
4A	Mid-latitude	Salt Lake City, USA (40.7856, -111.9706)	12.13 (-1.1/27.61)	0.48	1.80	2.90	46.81	49.71
5B	Mid-latitude	Denver, USA (39.8439, -104.6633)	10.53 (-2.2/24.02)	0.41	1.71	1.43	48.03	49.46
6A	Mid-latitude	Ottawa, Canada (45.3175, -75.6786)	7.05 (-8.4/21.34)	0.34	2.14	0	43.26	43.26
6A	Mid-latitude	Helsinki, Finland (60.2508, 25.0494)	6.26 (-8/17.88)	0.21	2.57	0	54.31	54.31
6A	Sub-artic	Ushuaia, Argentina (-54.8444, -68.3006)	4.59 (1.38/8.86)	0.003	3.23	0	93.02	93.02
7	Sub-artic	Tampere, Finland (61.4228, 23.6181)	5.5 (-6.2/16.59)	0.18	2.96	0	64.58	64.58
7	Mid-latitude	Calgary, Canada (51.1231, -114.0228)	4.53 (-7.6/16.98)	0.20	2.36	0	53.90	53.90
7	Mid-latitude	Ekaterinburg, Russia (56.7494, 60.805)	3.63 (-14.5/19.11)	0.28	2.67	0	52.65	52.65
7	Sub-arctic	Novosibirsk, Russia (55.0217, 82.6517)	2.49 (-17.8/19.31)	0.29	2.76	0	64.59	64.59
7	Sub-arctic	Qaqortoq, Greenland (60.7172, -46.05)	1.29 (-4.8/8.3)	0	4.08	0	0	0
8	Sub-arctic	Yakutsk, Russia (60.0172, 129.7172)	-6.77 (-35.8/20.77)	0.48	6.57	0	153.94	153.94
8	Sub-arctic	Resolute, Canada (74.7169, -94.9711)	-14.1 (-31.4/4.41)	0	3.85	0	0	0

References in Supplementary Information

1. J. Li, X. Wang, D. Liang, et al., “A tandem radiative/evaporative cooler for weather-insensitive and high-performance daytime passive cooling,” *Science Advances* 8, no. 32 (2022): eabq0411, https://doi.org/10.1126/sciadv.abq0411
2. Psycometric Calculator, “Psycometric Calculator,” (2020), https://www.kwangu.com/work/psychrometric.htm
3. H. Esmaeilzadeh, M. Rivard, E. Arzi, F. Légaré, and A Hassani, “Smart textile plasmonic fiber dew sensors,” *Optics Express* 23, no. 11 (2015): 14981-14992, https://doi.org/10.1364/OE.23.014981
4. C. Wang, L. Hua, H. Yan, B. Li, Y. Tu, and R. Wang, “A thermal management strategy for electronic devices based on moisture sorption-desorption processes,” *Joule* 4, no. 2 (2020): 435-447, https://doi.org/10.1016/j.joule.2019.12.005
5. M. Dong, Z. Zhang, Y. Shi, X. Zhao, S, Fan, and Z. Chen, “Fundamental limits of the dew-harvesting technology,” *Nanoscale and Microscale Thermophysical Engineering* 24, no. 1 (2020): 43-52, https://doi.org/10.1080/15567265.2020.1722300
6. S. Wang, T. Jiang, Y. Meng, R. Yang, G. Tan, and Y. Long, “Scalable thermochromic smart windows with passive radiative cooling regulatio,.” *Science* 374, no. 6574 (2021): 1501-1504, https://doi.org/10.1126/science.abg0291
7. S.-K. Kim, G.-H. Lee, C. Jeon, et al., “Bimetallic nanocatalysts immobilized in nanoporous hydrogels for long-term robust continuous glucose monitoring of smart contact lens,” *Advanced Materials* 34, no. 18 (2022): 2110536, https://doi.org/10.1002/adma.202110536
8. Y. Guo, W. Guan, C. Lei, H. Lu, W. Shi, and G. Yu, “Scalable super hygroscopic polymer films for sustainable moisture harvesting in arid environments,” *Nature Communications* 13 (2022): 2761, https://doi.org/10.1038/s41467-022-30505-2
9. L. Zhang, H. Xia, F. Xia, Y. Du, Y. Wu, and Y. Gao, “Energy-saving smart windows with HPC/PAA hybrid hydrogels as thermochromic materials,” *ACS Applied Energy Materials* 4, no. 9 (2021): 9783-9791, https://doi.org/10.1021/acsaem.1c01854
10. A. Nakamura, R. Ogai, and K. Murakami, “Development of smart window using an hydroxypropyl cellulose-acrylamide hydrogel and evaluation of weathering resistance and heat shielding effect,” *Solar Energy Materials and Solar Cells* 232 (2021): 111348, https://doi.org/10.1016/j.solmat.2021.111348

11. S. Dou, X. Hu, J. Dong, et al., “Passive heating into designable temperatures with thermal responsive hydrogel for smart thermal managements,” *Science China Materials* 68 (2025): 2014-2023, https://doi.org/10.1007/s40843-025-3312-9
12. G. Park, H. Park, J. Seo, et al., “Bidirectional thermo-regulating hydrogel composite for autonomic thermal homeostasis,” *Nature Communications* 14 (2023): 3049, https://doi.org/10.1038/s41467-023-38779-w
13. S. Pu, J. Pu, Y. Liao, et al., “Promoting energy efficiency via a self-adaptive evaporative cooling hydrogel,” *Advanced Materials* 32, no. 17 (2020):1907307, https://doi.org/10.1002/adma.201907307
14. J. Gu, H. Wei, F. Ren, et al., “VO_2-based infrared radiation regulator with excellent dynamic thermal management performance,” *ACS Applied Materials and Interfaces* 14, no. 2 (2022): 2683-2690, https://doi.org/10.1021/acsami.1c17914
15. X. Ao, B. Li, B. Zhao, et al., “Self-adaptive integration of photothermal and radiative cooling for continuous energy harvesting from the sun and outer space,” *Proceedings of the National Academy of Sciences* 119, no. 17 (2022): e2120557119, https://doi.org/10.1073/pnas.2120557119
16. S. Wang, Y. Zhou, T. Jiang, R. Yang, G. Tan, and Y. Long, “Thermochromic smart windows with highly regulated radiative cooling and solar transmission,” *Nano Energy* 89 (2021): 106440, https://doi.org/10.1016/j.nanoen.2021.106440
17. H. Yao, H. Cheng, Q. Liao, et al., “Integrated radiative and evaporative cooling beyond daytime passive cooling power limit,” *Nano Research Energy* 2, no. 2 (2023): e9120060-e9120060, https://doi.org/10.26599/NRE.2023.9120060
18. T. Wang, Y. Zhang, M. Chen, M. Gu, and L. Wu, “Scalable and waterborne titanium-dioxide-free thermochromic coatings for self-adaptive passive radiative cooling and heating,” *Cell Reports Physical Science* 3, no. 3 (2022): 100782, https://doi.org/10.1016/j.xcrp.2022.100782
19. Q. Zhang, Y. Lv, Y. Wang, et al., “Temperature-dependent dual-mode thermal management device with net zero energy for year-round energy saving,” *Nature Communications* 13 (2022): 4874, https://doi.org/10.1038/s41467-022-32528-1
20. X. Mei, T. Wang, M. Chen, and L. Wu, “A self-adaptive film for passive radiative cooling and solar heating regulation,” *Journal of Materials Chemistry A* 10 (2022): 11092-11100, https://doi.org/10.1039/D2TA01291J

21. C. Zhang, J. Yang, Y Li, et al., "Vapor–liquid transition-based broadband light modulation for self-adaptive thermal management," *Advanced Functional Materials* 32, no. 48 (2022): 2208144, https://doi.org/10.1002/adfm.202208144
22. D. Xie, W. Li, C. A. Richards, et al., "Thermally responsive hydrogels for passive temperature regulation under direct sunlight," *Advanced Photonics Research* 4, no. 4 (2023): 2200253, https://doi.org/10.1002/adpr.202200253
23. C. Lin, J. Hur, C. Y. H. Chao, et al., "All-weather thermochromic windows for synchronous solar and thermal radiation regulation," *Science Advances* 8, no. 17 (2022): eabn7359, https://doi.org/10.1126/sciadv.abn7359
24. R. Zhang, R. Li, P. Xu, et al., "Thermochromic smart window utilizing passive radiative cooling for self-adaptive thermoregulation," *Chemical Engineering Journal* 471 (2023): 144527, https://doi.org/10.1016/j.cej.2023.144527
